# Physiological and Transcriptome Analysis of Drought Tolerance in Barley

**DOI:** 10.3390/plants15203087

**Published:** 2026-10-09

**Authors:** Farzaneh Karamzadeh, Ahmad Arzani, Ghodratollah Saeidi, Narges Gharaghanipor, Fabrizio Araniti

**Affiliations:** 1 Department of Agronomy and Plant Breeding, College of Agriculture, Isfahan University of Technology, Isfahan 8415683111, Iran; farzaneh.karamzadeh69@gmail.com (F.K.); gsaeidi@iut.ac.ir (G.S.); n.gharaghanipor@gmail.com (N.G.); 2 Dipartimento di Scienze Veterinarie, University of Messina, Viale G. Palatucci sn, 98168 Messina, Italy

**Keywords:** HPLC, gene expression, mRNA transcript, RNA-seq, water-deficit stress

## Abstract

Drought stress is the major environmental constraint in crop production. As climate change increases drought frequency, enhancing drought tolerance has become a major goal in barley breeding. *Hordeum vulgare* ssp. *spontaneum*, the wild and closest relative of cultivated barley, is a valuable genetic resource for breeding and developing drought-tolerant cultivars. This study aimed to compare physiological and transcriptomic responses of a drought-tolerant wild genotype (*H. vulgare* ssp. *spontaneum*) and a drought-sensitive cultivated barley genotype (‘Mona’ cultivar) under control and drought stress conditions. The wild genotype exhibited less physiological and growth impairment than the ‘Mona’ cultivar under drought stress. Transcriptome profiling identified 2564 differentially expressed genes (DEGs) in the drought-tolerant wild genotype and 2163 DEGs in the drought-sensitive cultivar. Notably, in the tolerant genotype, most DEGs were up-regulated, whereas in the sensitive cultivar, down-regulation was more frequently induced by drought. GO enrichment analysis revealed that the DEGs were predominantly associated with key stress-related biological processes, including cellular component organization, signaling pathways, ion transport, regulatory protein activity, reactive oxygen species (ROS) scavenging, hormone biosynthesis, and osmotic homeostasis. Comparative analysis showed that the drought-tolerant genotype harbored more functionally effective drought-responsive genes and had higher transcript abundance than the sensitive genotype. Overall, the tolerant genotype consistently exhibited superior physiological and functional performance under drought stress. These findings deepened our understanding of the evolutionary basis of drought tolerance in wild barley, while the shared functional mechanisms between the two subspecies identified physiological and transcriptional traits that could be exploited to improve barley under water-limited environments.

## 1. Introduction

Due to global climate anomalies, drought stress poses an increasing threat to plant growth worldwide and leads to yield loss in major crops and consequent socioeconomic crises [1]. Since 2000, the number and duration of droughts have increased by 29% compared to the last two decades [2] (WMO, 2021). Water constitutes roughly 80–95% of a plant’s fresh biomass and is essential for numerous physiological processes, underpinning plant growth, development, and metabolic activities [3]. Plants have evolved a series of adaptive and tolerant strategies to cope with drought stress. Some plants can maintain water balance under water deficit by increasing root water uptake from the soil, reducing water loss through stomatal closure, and adjusting osmotic pressure by accumulating osmolytes in cell tissues [4]. Drought impairs mineral nutrient movement from roots to shoots by lowering transpiration rates and altering membrane transporter activity. Therefore, understanding how drought stress affects plant mineral nutrition can help identify effective strategies to mitigate drought damage and resulting nutrient deficiencies. The role of mineral nutrients in increasing or decreasing drought stress tolerance of plants has been studied by several researchers; however, current knowledge remains limited and partly unclear [5]. Gaining deeper insight into the complex mechanisms of plant acclimation to water deficit has great importance to scientists, breeders, and agronomists seeking to secure crop yields under unpredictable water-limited conditions [1].

In recent years, rapid advances and reduced costs in next-generation sequencing (NGS) have led to its widespread application in crop genetics and breeding to improve stress tolerance [4]. RNA-seq is widely regarded as the most powerful and versatile approach for genome-wide analysis of gene expression and transcriptional activity, and its analytical complexity has driven extensive research into specialized algorithms and methods. The RNA-seq shows strong agreement with gold-standard techniques like RT-qPCR for measuring both absolute and relative gene expression, making it the preferred method for transcriptomic analysis [6]. Over the past decade, studies have used RNA-seq to compare the transcriptome changes induced by drought stress in the tolerant and sensitive genotypes in barley [7], wheat [8], soybean [9], sweet potato [10], and rice [11]. Despite these advances, most studies have employed single-layer approaches that offer only a partial view of drought adaptation. ICP-MS-based ionomics quantifies stress-induced changes in elemental composition but cannot resolve the upstream regulatory networks governing nutrient transport, vacuolar sequestration, or membrane transporter activity. Conversely, transcriptomics identifies shifts in gene expression but does not indicate whether these regulatory changes translate into functional nutrient homeostasis. Integrating ionomics with transcriptomics overcomes these limitations by linking elemental phenotypes to underlying regulatory networks, enabling a more comprehensive, mechanistic understanding of drought responses. This combined approach is particularly valuable in barley, where domestication may have weakened key regulatory pathways needed to maintain ion homeostasis under water-deficit conditions.

Barley (*Hordeum vulgare* L.) has emerged as a model crop in molecular biology and genetics research, especially for studies on abiotic stress tolerance [12]. Wild barley (*H. vulgare* L. ssp. *spontaneum*) is the direct ancestor of cultivated barley (*H. vulgare* L. ssp. *vulgare*), and the two subspecies are fully reproductively compatible [7,13,14]. Consequently, wild barley has long been recognized as a valuable source of genetic resources for introgression into modern cultivars to enhance their resilience and tolerance to environmental stresses [7]. Recent studies on Iranian wild barley have provided important insights into its drought-adaptive mechanisms. Iranian *H. spontaneum* populations, despite their unique ecological adaptation to arid and semi-arid environments, exhibit coordinated morphological, physiological, and biochemical strategies that enhance drought resilience. Shirvani et al. [15] demonstrated that drought-tolerant wild genotypes maintained higher chlorophyll content, relative water content, antioxidant activity, and expression levels of stress-responsive genes under water deficit. Karamzadeh et al. [16] further showed that increased phenolic accumulation and ABA-mediated antioxidant pathways helped mitigate oxidative damage in wild barley. Additionally, Barati et al. [17] highlighted the superior root development and physiological performance of wild barley relatives across contrasting water environments. Collectively, these findings indicate that wild barley employs integrated drought-adaptive mechanisms; however, comprehensive comparisons with cultivated barley remain limited, underscoring the need for studies that clarify how these mechanisms interact during early drought exposure.

Improving drought tolerance in barley has become increasingly important; however, breeding progress remains slow because modern cultivars face two major constraints: an extremely narrow genetic background resulting from decades of intensive selection for yield and agronomic traits, and a limited availability of truly drought-tolerant germplasm within cultivated barley. As a result, many elite cultivars lack the regulatory variants required to maintain ion homeostasis, activate antioxidant defenses, and coordinate hormone-mediated signaling under water deficit. In addition to these breeding limitations, a critical conceptual question remains insufficiently addressed: Has domestication weakened key regulatory pathways essential for effective drought adaptation? Emerging evidence suggests that cultivated barley may have lost part of its ancestral capacity to maintain ion homeostasis under water deficit, particularly the ability to efficiently sequester and compartmentalize toxic ions through vacuolar transport systems and membrane transporter regulation. Such domestication-driven attenuation of regulatory strength could fundamentally constrain drought resilience in elite cultivars. Therefore, a more mechanistic hypothesis posits that the superior drought tolerance of *H. spontaneum* arises not only from stronger activation of antioxidant, hormonal, and transcriptional pathways, but also from the preservation of regulatory mechanisms that have been diminished or lost during domestication—especially those governing ion transport, vacuolar sequestration, and upstream transcriptional control. Studying drought tolerance in barley can reveal the genetic factors behind stress responses, helping develop climate-resilient cultivars with stable yields in drought-prone regions. Transcriptome analyses can help identify the genes and mechanisms underlying the varied early responses of different barley genotypes to water deficit [12]. Understanding drought tolerance requires integrating physiological, biochemical, nutritional, and molecular responses, as drought adaptation is governed by complex, interconnected regulatory networks. The remarkable genetic diversity of wild barley populations, which enables their survival in low-input and marginal environments, represents a valuable resource for uncovering adaptive mechanisms that may have been lost during domestication [18]. Although researchers have extensively studied drought-responsive genes in cultivated barley, much less is known about how physiological, nutritional, and transcriptomic responses are coordinated in wild barley genotypes naturally adapted to water-limited environments. Despite numerous studies on drought responses in barley, most existing research suffers from methodological limitations that restrict their ability to fully explain drought-induced regulatory mechanisms. Previous transcriptomic studies have provided a relatively limited view of drought-induced molecular changes, overlooking the broader coordinated responses occurring across physiological, biochemical, and nutritional dimensions. Comparative analyses between wild and cultivated barley have also been limited and lack the systematic evaluation of how domestication has shaped stress-responsive regulatory pathways. Furthermore, few studies have integrated mineral nutrient distribution (ionomics) with physiological traits, antioxidant activity, osmotic adjustment, and transcriptomic reprogramming within a unified multi-dimensional framework. As a result, the mechanisms through which wild barley maintains nutrient homeostasis and activates stress-related transcriptional networks under water deficit remain insufficiently characterized. These gaps highlight the need for comprehensive studies that combine physiological, biochemical, nutritional, and transcriptomic analyses to reveal the adaptive strategies that distinguish wild barley from cultivated forms. This study addresses these limitations by applying an integrated multi-omics approach to dissect early drought responses in wild and cultivated barley, providing novel insights into the regulatory networks underlying superior drought tolerance in *H. spontaneum*. In particular, the contribution of nutrient homeostasis and stress-related transcriptional reprogramming to drought adaptation remains insufficiently characterized. We hypothesized that the superior drought tolerance of *H. spontaneum* results from the coordinated regulation of antioxidant defense, nutrient homeostasis, hormone signaling, and stress-responsive transcriptional networks under water-deficit conditions.

## 2. Results

### 2.1. Growth and Physiological Traits

Drought stress adversely affected plant height and fresh weight, and the sensitive cultivar was more susceptible to these negative effects. Drought stress caused a pronounced decline in several key physiological traits of barley plants, including relative water content (RWC), membrane stability index (MSI), chlorophyll a, chlorophyll b, carotenoid content, and gibberellin (GB). In contrast, levels of proline, peroxidase (POX), total phenolic content (TPC), total flavonoid content (TFC), phenolic compositions, 2,2-diphenyl-1-picrylhydrazyl (DPPH) activity, malondialdehyde (MDA), hydrogen peroxide (H_2_O_2_), and abscisic acid (ABA) were increased markedly in barley seedlings. The reduction in RWC was substantially greater in the cultivated genotype (63%) than in the wild genotype (38%). Similarly, MSI dropped by 58.52% in the cultivated barley, indicating more pronounced membrane injury. These quantitative differences demonstrated that the cultivated genotype experienced a sharper disruption in cellular water balance and membrane integrity under drought stress. In contrast, the smaller decline in RWC in the wild genotype reflected its superior ability to maintain cellular hydration and sustain turgor, which was essential for preserving metabolic activity during water deficit. Likewise, the more moderate reduction in MSI in wild barley indicated lower membrane damage, suggesting stronger structural stability and more effective protective mechanisms such as osmotic adjustment and antioxidant activity. Together, these patterns highlighted that the wild genotype underwent considerably less physiological impairment under drought, underscoring its inherently higher drought tolerance. The tolerant genotype showed a less pronounced decline in chlorophyll a and chlorophyll b than the susceptible genotypes. Wild barley also exhibited a dramatic accumulation of proline up to 220%, alongside stronger antioxidant responses, including greater increases in POX, TPC, TFC, and DPPH activity. Notably, even under control conditions, the cultivated genotype showed higher MDA and H_2_O_2_ levels, which rose even more sharply under drought, highlighting its greater oxidative susceptibility. These coordinated physiological responses indicated that tolerant genotypes maintained photosynthetic function more effectively and deployed stronger osmotic and antioxidant defenses under stress. In contrast, the elevated baseline and drought-induced oxidative markers in the cultivated genotype reflected weaker protective capacity and greater vulnerability to ROS-mediated damage.

**Figure 1:**
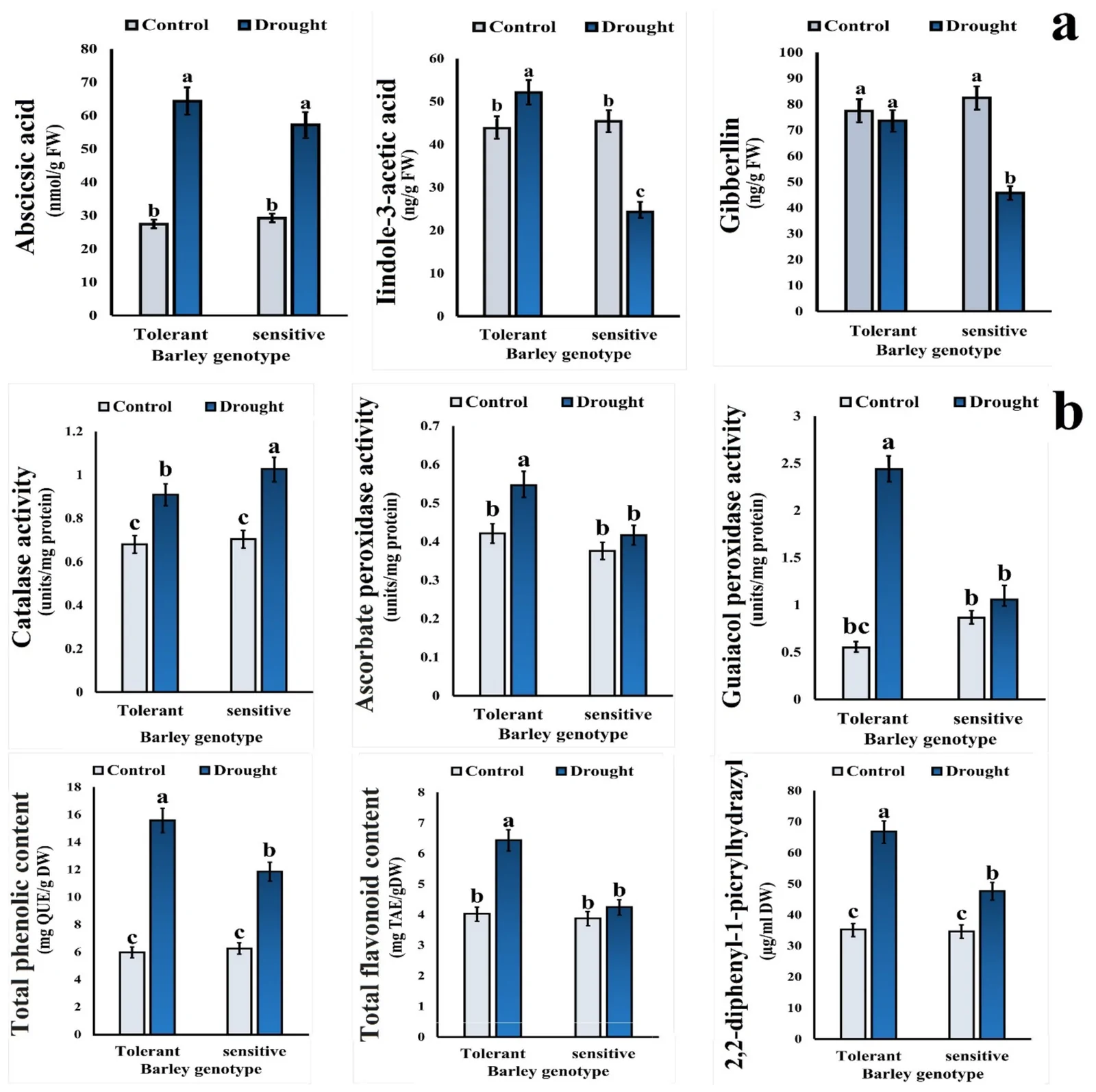

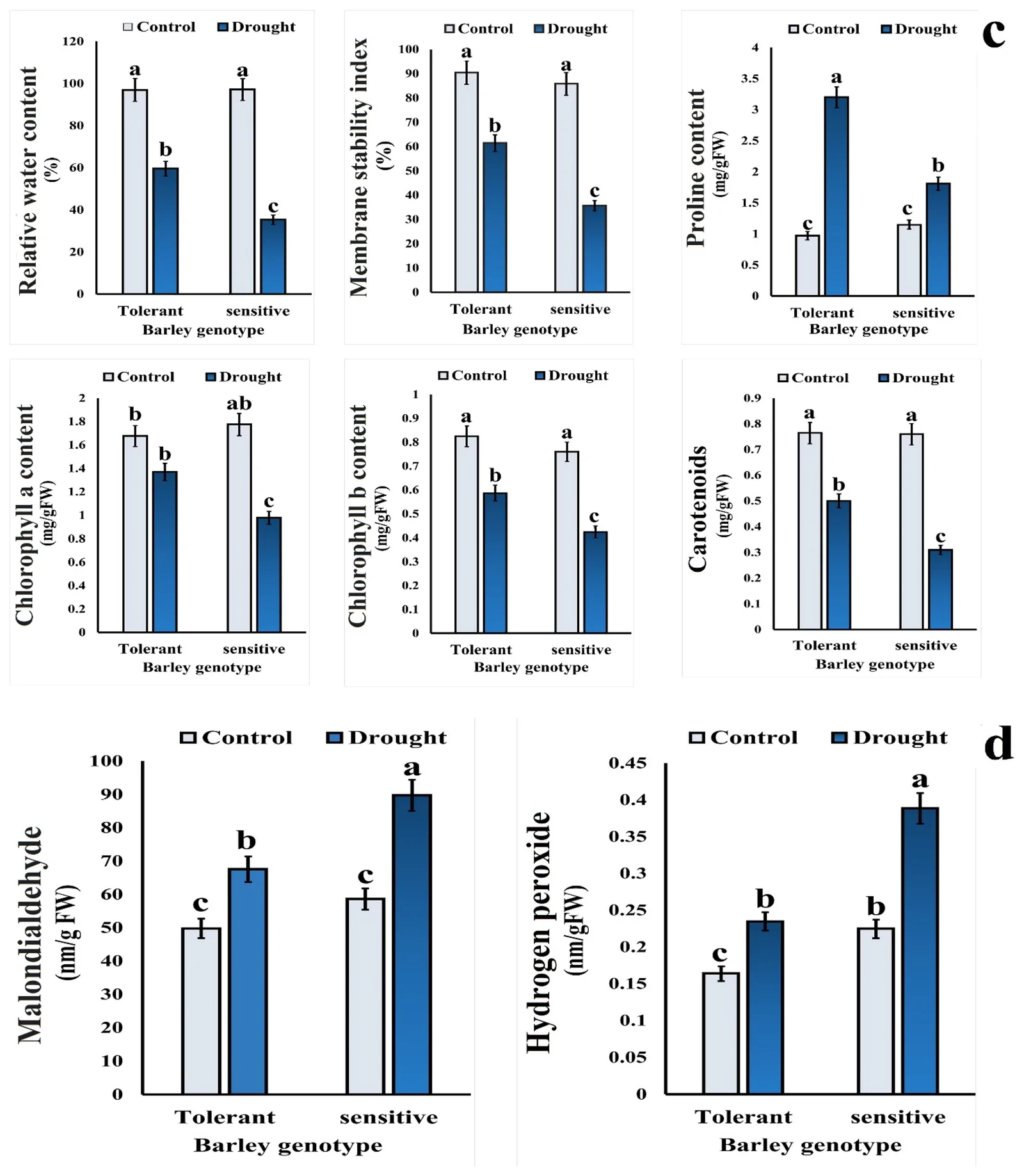

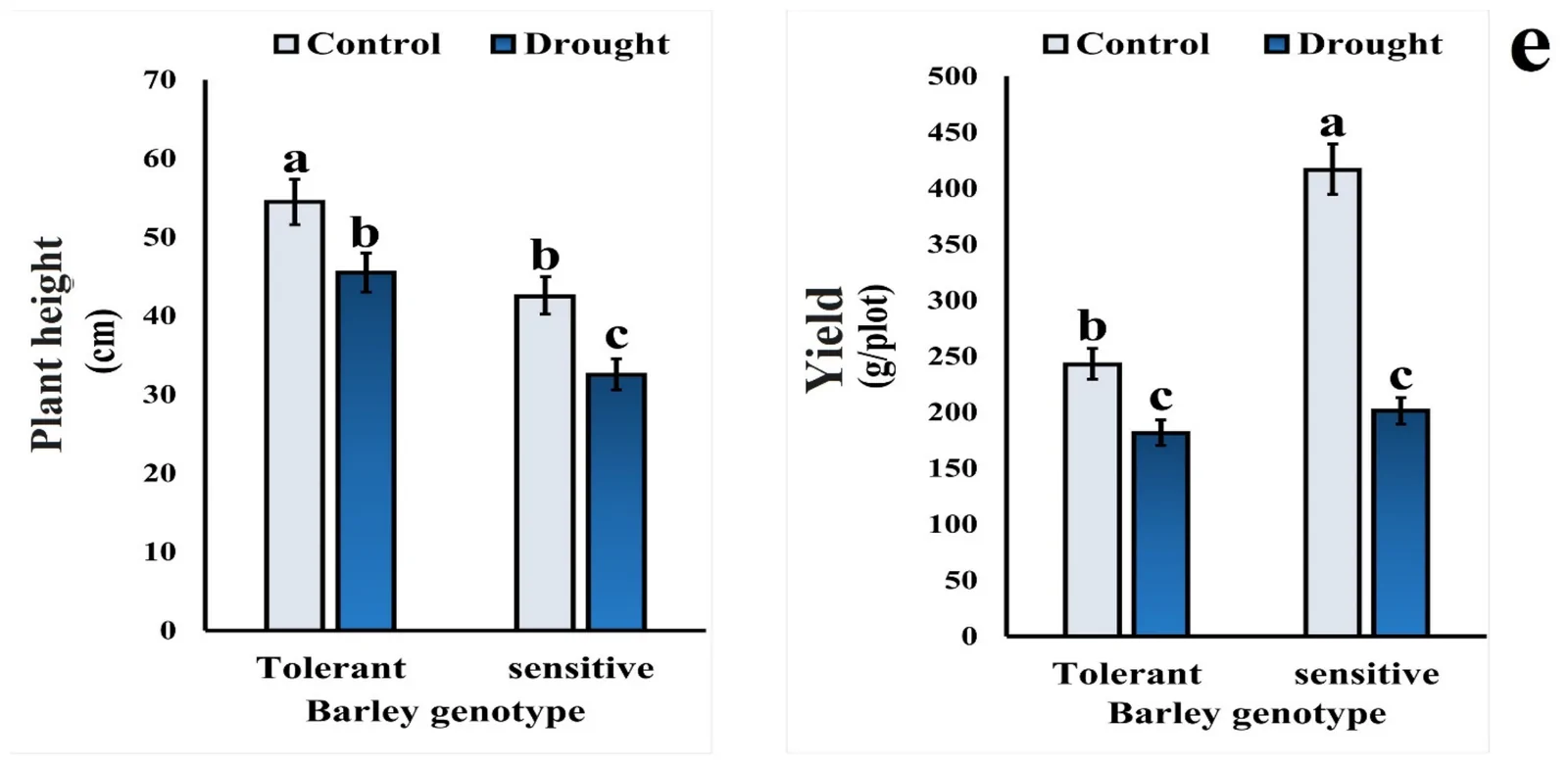
Mean comparisons of (**a**) plant hormones [abscisic acid (ABA), indole-3-acetic acid (IAA) and gibberellin (GB)], (**b**) enzymatic antioxidants [Guaiacol peroxidase activity (POX), Ascorbate peroxidase activity (APX) and Catalase activity (CAT)] and non-enzymatic antioxidants [total phenolic content (TPC), total flavonoid content (TFC) and 2,2-diphenyl-1-picrylhydrazyl (DPPH)], (**c**) Physiological parameters (membrane stability index (MSI), relative water content (RWC), chlorophyll a content, chlorophyll b content, carotenoids and proline content, (**d**) oxidative stress indicators [malondialdehyde (MDA) and hydrogen peroxide (H_2_O_2_)] (**e**) plant height and yield of wild drought-tolerant and ‘Mona’ drought-sensitive barleys genotypes under control and drought stress conditions. Bars represent means ± SE. For each trait, bars with the same letter are not significantly different at *p* < 0.05 according to the LSD test, across the genotypes grown under control and drought stress conditions.

Hormonal profiling further revealed that wild genotypes maintained higher baseline hormone levels. Under drought stress, ABA increased by 134% in wild genotypes compared to 95% in cultivated ones. Conversely, IAA and GB declined more sharply in cultivated genotypes (46% and 44%, respectively). In wild genotypes, IAA exhibited only a modest increase (19%), while GB showed no significant change (Figure 1). These quantitative differences indicate that wild genotypes activated a more effective hormonal adjustment under drought, with stronger ABA induction supporting stomatal regulation and stress signaling. In contrast, the pronounced reductions in IAA and GB in cultivated genotypes reflected impaired growth regulation and weaker osmoprotective capacity. Overall, the more stable hormonal profile of wild barley under drought highlighted its superior regulatory resilience and contributed to its higher stress tolerance.

#### Elemental Analysis Using ICP-MS

In this study, drought stress triggered contrasting patterns of mineral element allocation between drought-sensitive and drought-tolerant genotypes. In the sensitive genotype, aluminium accumulation increased in grains, whereas the tolerant genotype showed reduced aluminium levels in both grains and leaves. Barium concentrations declined in both genotypes, with a stronger reduction in leaves. Copper levels remained unchanged in the sensitive genotype but increased threefold (200%) in grains of the tolerant genotype (Table 1).

Calcium also differed markedly between genotypes, displaying a contrasting pattern: grain calcium rose in the sensitive genotype but remained stable in the tolerant one. In leaves, calcium decreased under drought in the sensitive genotype, yet it increased in the tolerant genotype. Potassium and phosphorus levels declined in both grains and leaves of the sensitive genotype, while both elements increased in the tolerant one. Iron concentrations increased in both genotypes, with greater accumulation in leaves. Manganese increased significantly in the tolerant genotype but remained unchanged in the sensitive one.

Magnesium and strontium decreased in grains of the sensitive genotype, whereas the tolerant genotype maintained stable levels. Zinc declined in both grains and leaves of the sensitive genotype, while grain zinc increased in the tolerant genotype. Furthermore, molybdenum, sodium, and sulfur concentrations increased in grains of the tolerant genotype, whereas sulfur decreased in leaves of the sensitive genotype.

Overall, the drought-tolerant genotype maintained or enhanced levels of essential nutrients (Ca, K, P, Mn, Mg, Zn, Mo, S, Fe) while restricting the accumulation of toxic or non-essential elements (Al, Ba, Sr). This nutrient-regulation strategy supported ionic homeostasis, improved photosynthetic efficiency, and may have contributed to maintaining grain quality under drought stress. These results align with previous findings in wheat, barley, and other cereals, underscoring the central role of mineral nutrition in drought tolerance mechanisms. The maintenance of potassium and phosphorus accumulation in the tolerant genotype was particularly noteworthy because these nutrients played central roles in osmotic adjustment, stomatal regulation, energy metabolism, and membrane integrity. Likewise, enhanced accumulation of micronutrients such as Fe, Zn, and Mn may have contributed to sustaining antioxidant enzyme activity and photosynthetic performance under drought stress.

### 2.2. Data Quality Assessment

Enrichment analysis was performed to classify DEGs of barley subspecies (wild and cultivated barley) in response to drought stress using DEseq2. The PCA plot, based on the distance matrix, showed the samples in a 2D plane spanned by their first two principal components. The stressed (blue and on the right side of the plot) and normal (red and on the left side of the plot) samples were well separated (Figure S1a,b). For wild barley and Mona, 92 and 99 percent of the variance were explained, respectively.

A heatmap of the distance matrix provided an overview of the similarities and dissimilarities among samples. The heatmap clearly showed that the samples clustered according to condition, with drought-stressed and normal samples forming distinct groups and exhibiting similar gene expression patterns within each group (Figure S2a,b).

### 2.3. Transcriptome Analysis

A deep RNA-seq analysis was performed on the transcriptome profiles of wild and cultivated barley leaf samples under normal and drought stress conditions. We obtained an average of 49 million clean reads (39–71 million read pairs) from the paired-end reads of the eight libraries (Table S1). High-quality trimmed reads were aligned with the reference barley genome sequence. Over 83% of clean reads from wild genotype RNA-seq data were uniquely mapped to the reference barley sequence. A mapping rate of 82–91% was achieved from the transcriptome data of the ‘Mona’ cultivar (Table S1). Overall, the RNA-Seq data analysis pipeline revealed clear distinctions between the two environments (drought vs. normal) and between the two genotypes.

**Figure 2:**
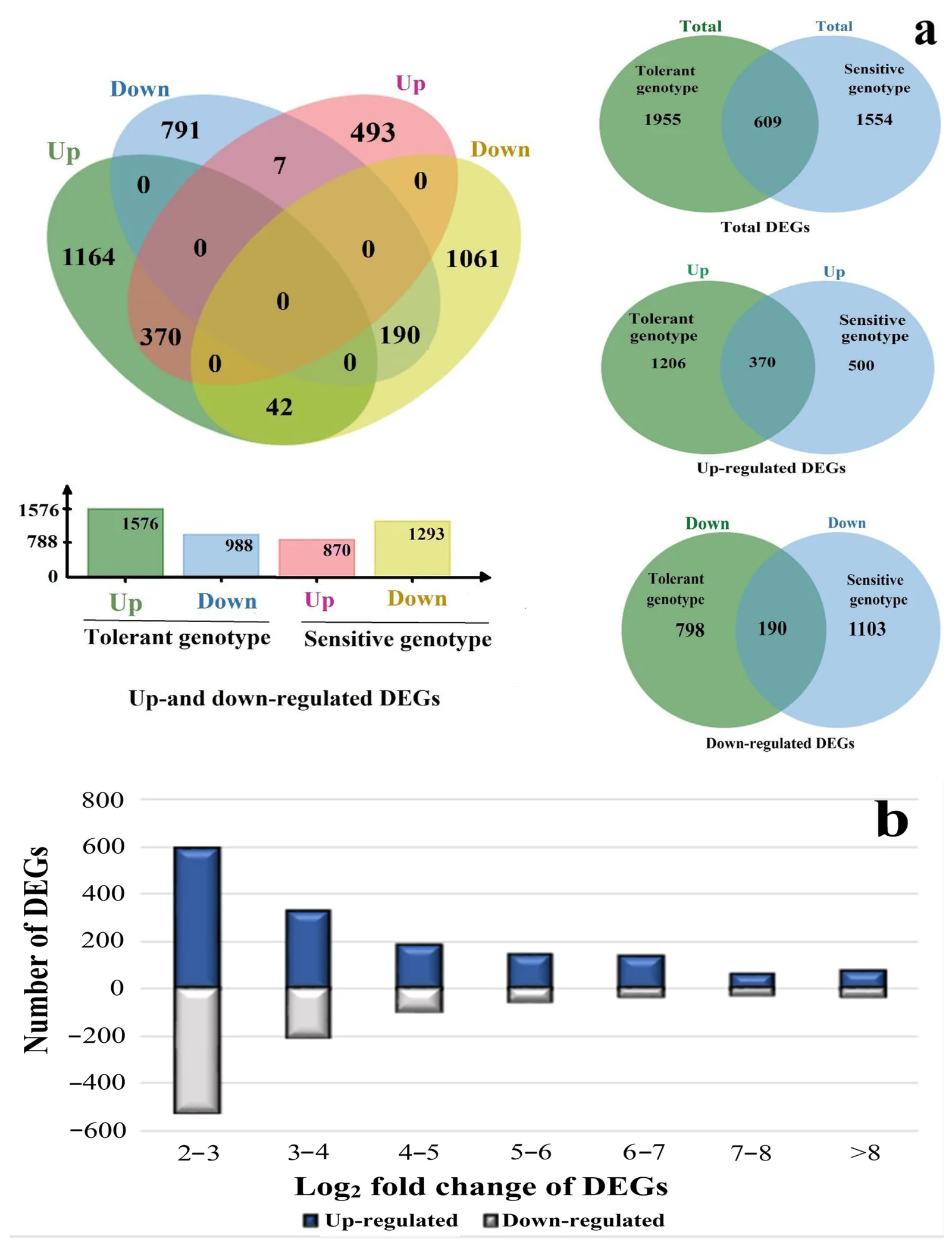

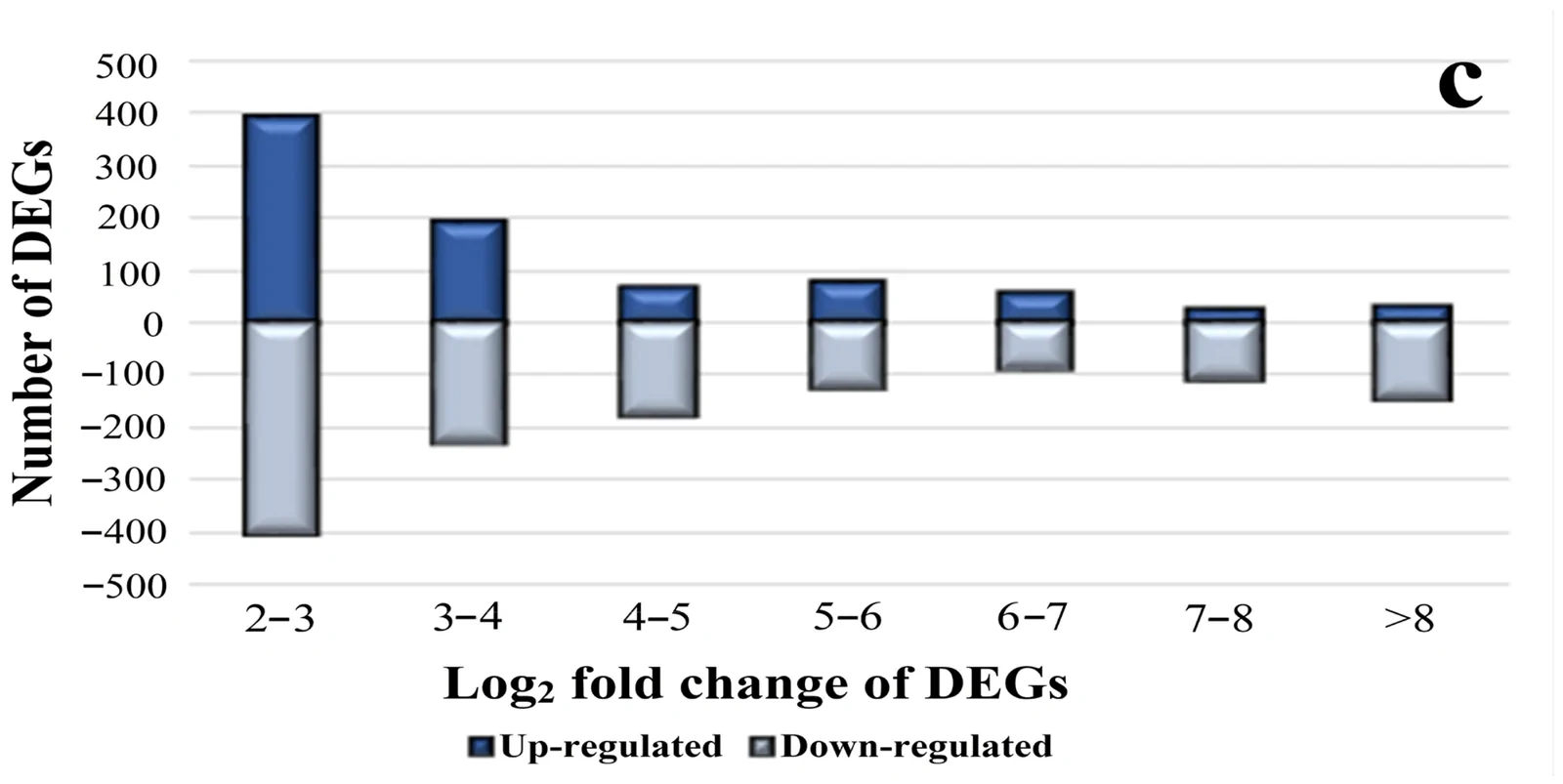
(**a**) Venn diagrams showing the number of distinct and common drought-responsive genes (DEGs) found in two barley subspecies (wild drought-tolerant genotype and ‘Mona’ drought-sensitive cultivar). DEGs were identified using standard thresholds (*p* value < 0.05, |log_2_FC| ≥ 2) and are presented as total, up-regulated, and down-regulated gene sets for each genotype. Histogram illustrating the distribution of log_2_ fold change (Log_2_ FC) values for drought-responsive differentially expressed genes (DEGs) of two barley subspecies: (**b**) wild genotype and (**c**) ‘Mona’ cultivar.

DEGs were analyzed to compare gene expression between the wild (tolerant) genotype and the sensitive cultivar ‘Mona’ under both normal and drought-stress treatments. In wild barley, 1576 genes were differentially expressed under drought stress, of which 988 were down-regulated and 1576 were up-regulated. Drought stress in the ‘Mona’ cultivar resulted in the up-regulation of 870 genes and down-regulation of 1293 genes (Figure 2a). A total of 609 DEGs were identified as common to the tolerant and sensitive genotypes in this study. However, the drought-tolerant genotype exhibited a markedly stronger transcriptional response, with a higher proportion of up-regulated genes and a larger number of genes displaying high log_2_ fold-change values (>8-log_2_ fold change). This suggested a more dynamic and coordinated activation of stress-responsive pathways compared with the sensitive cultivar. In both genotypes, most DEGs showed a log_2_ fold change values between 2 and 3. However, in drought-tolerant wild barley, 83 DEGs had a log_2_ fold change values greater than 8, compared to only 35 such DEGs in the drought-sensitive barley (Figure 2b,c).

### 2.4. Heatmap of the Most Significant DEGs

**Figure 3:**
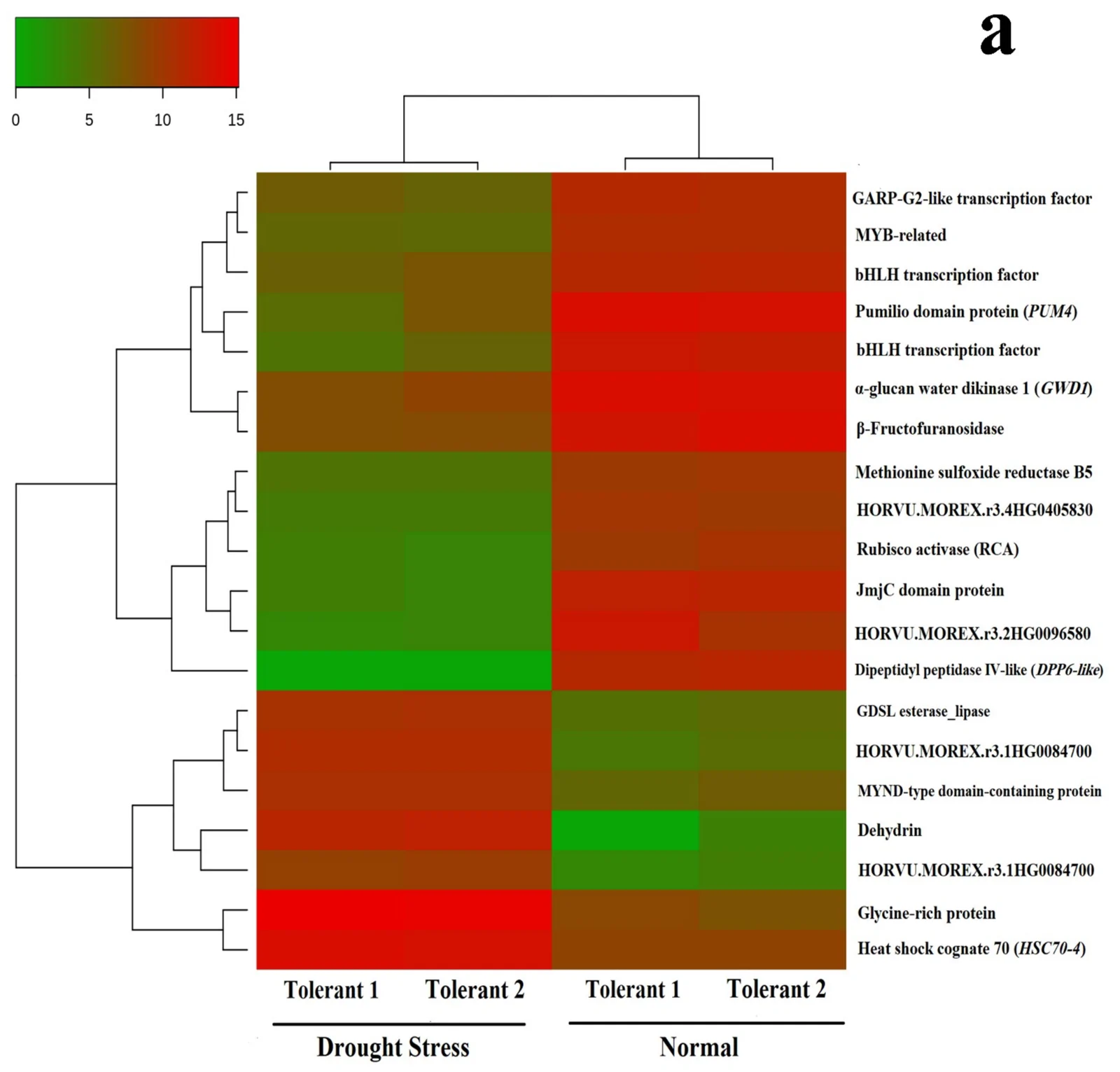

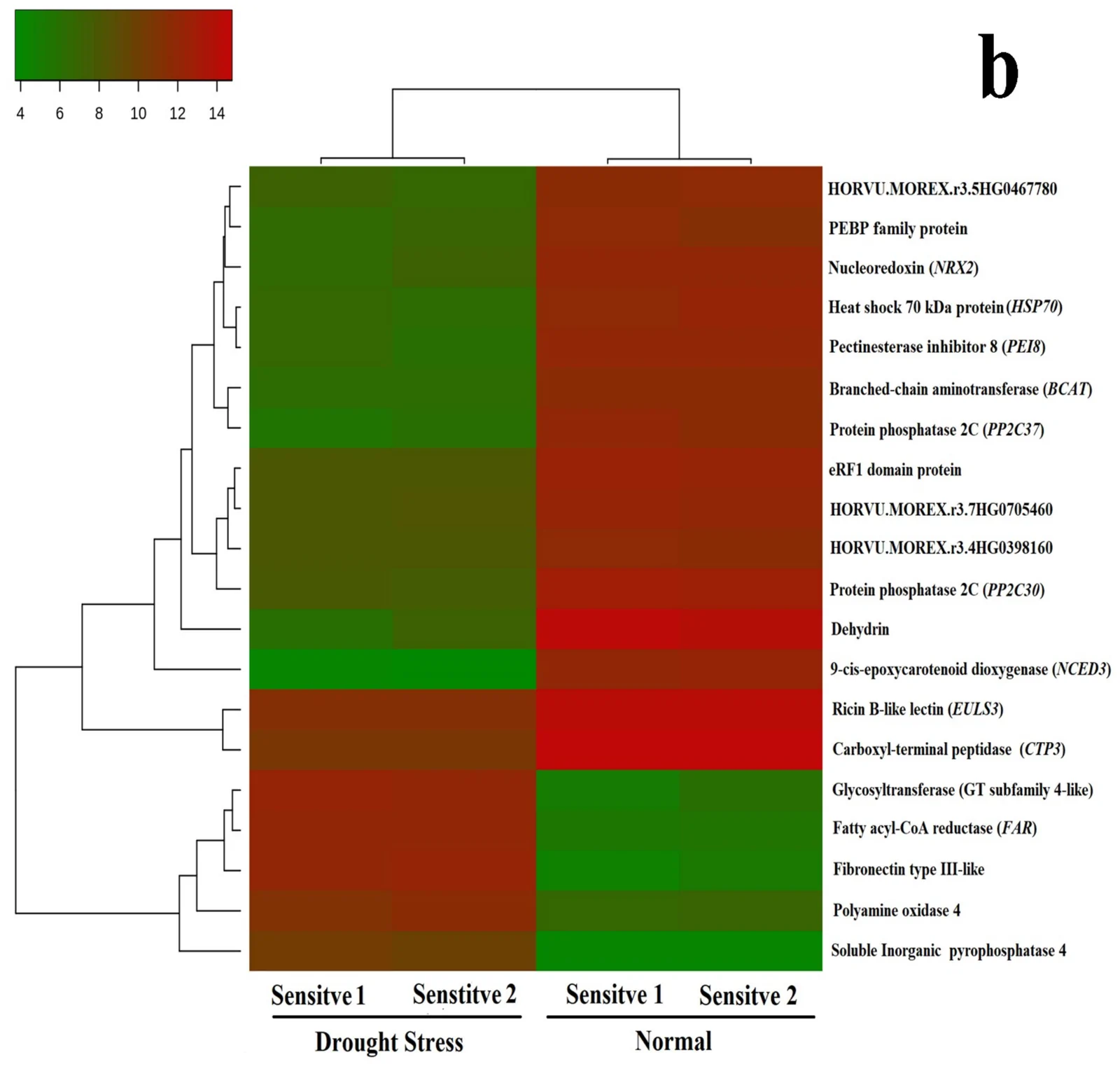
Heatmaps illustrating the expression patterns of the 20 most significantly differentially expressed genes (DEGs) under normal and drought stress conditions using DEseq2 related to (**a**) the drought-tolerant genotype vs. normal-tolerant and (**b**) drought-sensitive cultivar vs. normal sensitive (Mona).

Among the DEGs, the 20 genes with the smallest adjusted *p*-values were selected for heatmap analysis, and the Heatmap was generated using normalised DESeq2 counts. The expression profiles revealed clear transcriptional contrasts between the drought-tolerant and drought-sensitive genotypes. In the drought-tolerant genotype, most key genes exhibited increased expression under drought stress and formed relatively uniform clusters across treatments. Regulatory, antioxidant-related, membrane-associated and metabolic genes—including several transcription factors, dehydrins, and *HSC70*—displayed coordinated up-regulation, indicating a stable and organized transcriptional adjustment to drought. In contrast, the drought-sensitive genotype “Mona” exhibited more fragmented clustering patterns and stronger drought-induced shifts in ABA-related genes (such as *NCED3* and *PP2C* members), heat shock proteins, and dehydrins. A clear separation between control and drought samples was observed, and many of the key genes showed overall reduced expression, reflecting a less coordinated and more stress-driven transcriptional response. Overall, the clustering patterns demonstrate distinct transcriptional strategies between the two genotypes, with the tolerant genotype showing a more coherent adaptive response, whereas the sensitive genotype exhibited sharper but less stable changes under drought stress (Figure 3).

### 2.5. Genotype × Treatment Interaction Identifies Genes with Divergent Drought Responses

Classification of the significant genotype × treatment interaction genes revealed distinct biological response groups (Figure S3). A total of 955 genes showed a drought-tolerant genotype-specific drought response, whereas 554 genes exhibited a drought-sensitive cultivar-specific response. In addition, 39 genes showed a shared drought response that was stronger in the wild genotype, whereas 20 genes showed a stronger shared response in cv. Mona. A further 65 genes were oppositely regulated between the two genotypes. Finally, 1722 genes fell below the significance threshold within each genotype but showed divergent drought-induced changes, indicating directionally different responses despite sub-threshold effects. This distribution demonstrates that genotype-dependent drought responses arise from multiple biological patterns, including genotype-specific induction, shared but quantitatively different responses, and opposite regulation. These categories provide a structured overview of the interaction-significant gene set and form the basis for downstream functional interpretation.

The genotype × treatment interaction analysis identified a discrete set of genes whose drought-induced expression changes differed significantly between the wild genotype and cv. Mona. As shown in the volcano plot, most genes clustered near zero on the interaction axis, indicating broadly similar drought responses between the two genotypes (Figure S4 and Table S2). A subset of genes, however, showed large positive or negative interaction coefficients consistent with genotype-dependent differences in log_2_ fold change. Several genes exhibited stronger drought induction in the wild genotype. For example, *HORVU.MOREX.r3.1HG0084700* (log_2_FC_wild = 6.25; log_2_FC_mona = 1.60), *HORVU.MOREX.r3.2HG0167040* (4.85 vs. 0.90), and *HORVU.MOREX.r3.1HG0031210* (4.35 vs. −2.70) showed substantially higher expression under drought in the wild genotype compared with Mona cultivar. Additional genes such as *HORVU.MOREX.r3.7HG0728710* and *HORVU.MOREX.r3.6HG0605940* were induced in the wild genotype but strongly down-regulated in Mona. Conversely, several genes showed stronger or opposite drought responses in cv. Mona. *HORVU.MOREX.r3.5HG0531040* (log_2_FC_wild = −4.24; log_2_FC_mona = 0.59), *HORVU.MOREX.r3.2HG0158950* (−4.51 vs. 1.15), and *HORVU.MOREX.r3.7HG0679650* (−8.53 vs. −2.55) were markedly down-regulated in the wild genotype but induced or less strongly repressed in Mona. Similar patterns were observed for *HORVU.MOREX.r3.2HG0200390*, *HORVU.MOREX.r3.3HG0285310*, and *HORVU.MOREX.r3.2HG0169880*. Overall, the volcano plot demonstrates that while the majority of genes respond similarly to drought in both genotypes, a defined subset shows statistically significant genotype-dependent differences in drought-induced expression, reflected by both the magnitude and direction of log_2_ fold changes.

### 2.6. Gene Ontology and KEGG Enrichment Analysis of DEGs

GO enrichment analysis was carried out to classify the total DEGs that were significantly enriched (Benjamini–Hochberg *p* ≤ 0.05) in response to drought stress across biological processes, molecular functions, and cellular components (Table 2). In response to drought stress, a total of 2446 up-regulated genes in wild and cultivated barley were categorized into 183 and 60 GO terms, respectively. For down-regulated genes, 234 GO terms were significantly enriched in drought-tolerant barley, while 446 GO terms were significantly enriched in drought-sensitive barley.

**Figure 4:**
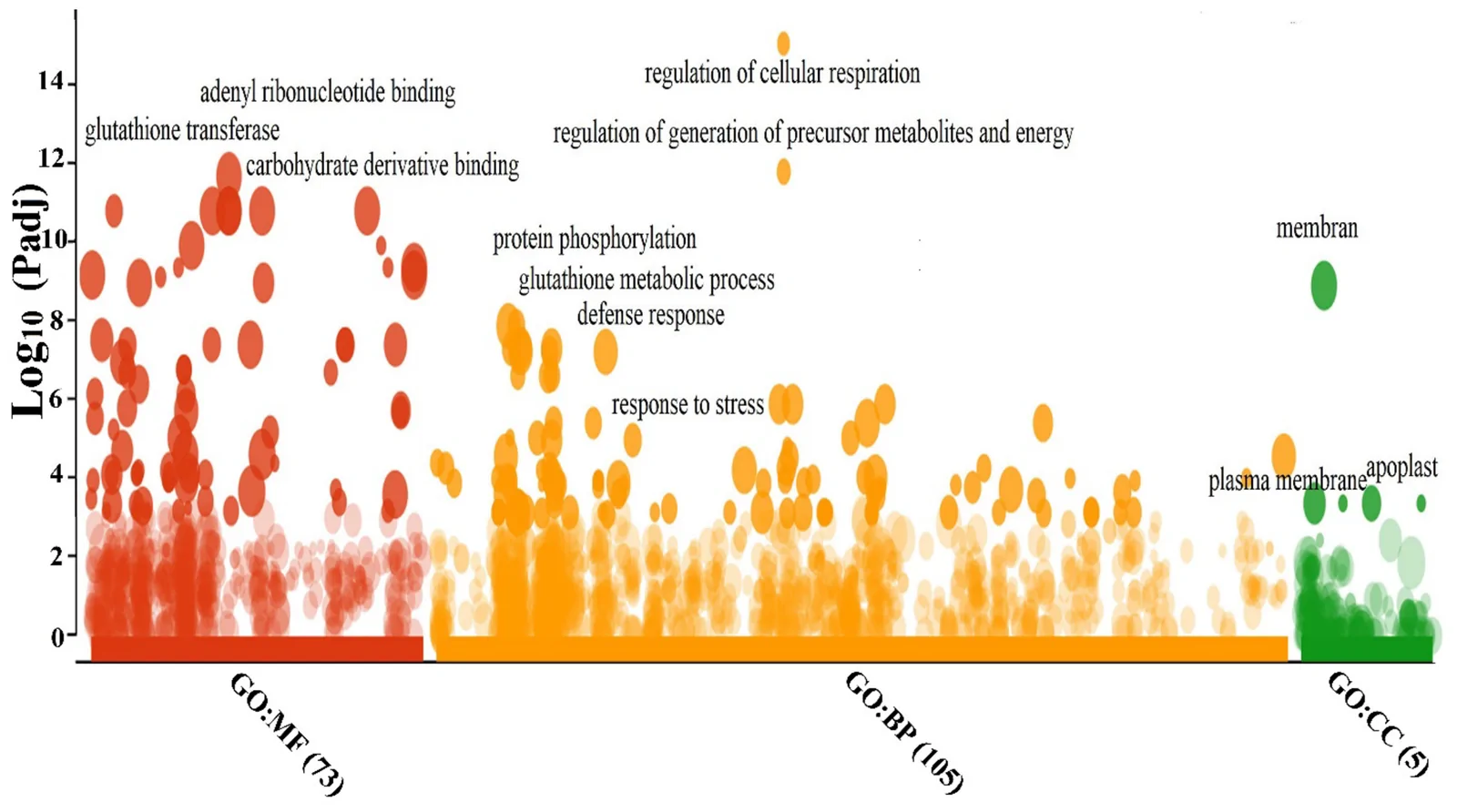
Gene Ontology (GO) enrichment analysis of up-regulated differentially expressed genes (DEGs) in the tolerant wild barley using the g: Profiler database Benjamini–Hochberg FDR test with a *p*-value ≤ 0.05 as a threshold. The GO enrichment bubble chart includes the three GO categories: Molecular Function (MF), Biological Process (BP), and Cellular Component (CC).

**Figure 5:**
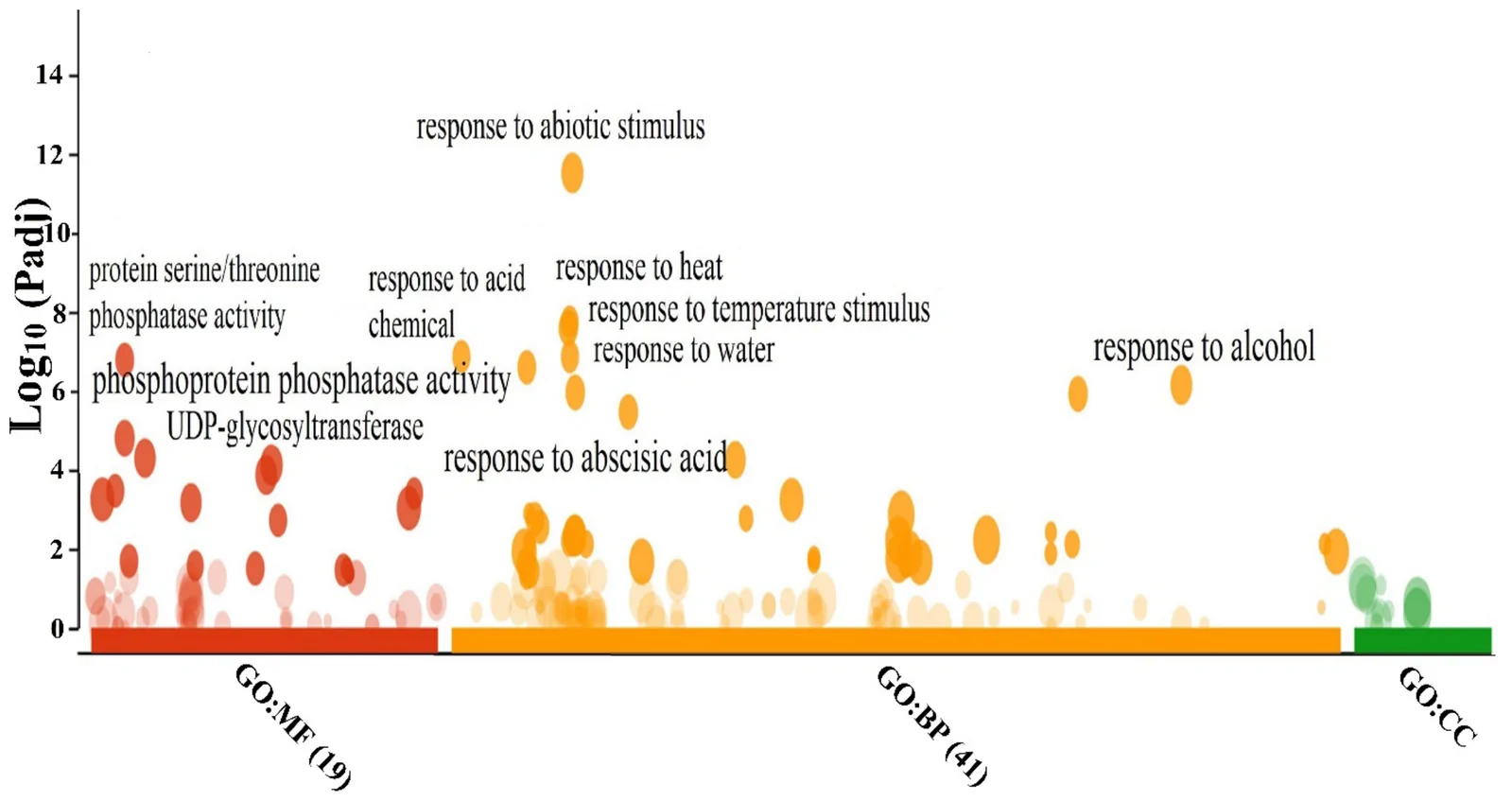
Gene Ontology (GO) enrichment analysis of up-regulated differentially expressed genes (DEGs) in the ‘Mona’ cultivar using the g: Profiler database Benjamini–Hochberg FDR test with a *p*-value ≤ 0.05 as a threshold. The GO enrichment bubble chart includes the three GO categories: Molecular Function (MF), Biological Process (BP), and Cellular Component (CC).

**Figure 6:**
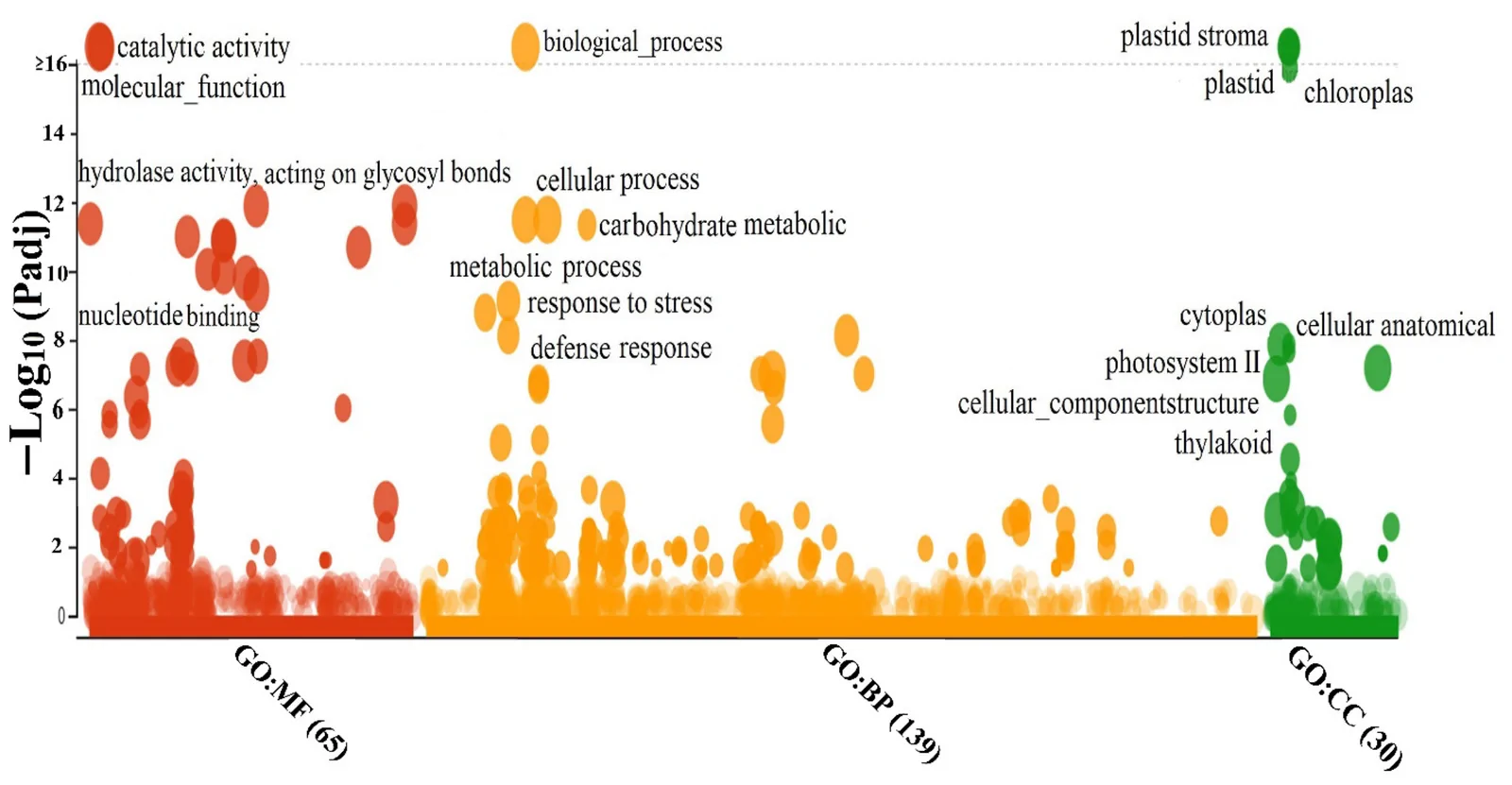
Gene Ontology (GO) enrichment analysis of down-regulated differentially expressed genes (DEGs) in wild barley using the g: Profiler database Benjamini–Hochberg FDR test, with a *p*-value ≤ 0.05 as a threshold. The GO enrichment bubble chart includes the three GO categories: Molecular Function (MF), Biological Process (BP), and Cellular Component (CC).

**Figure 7:**
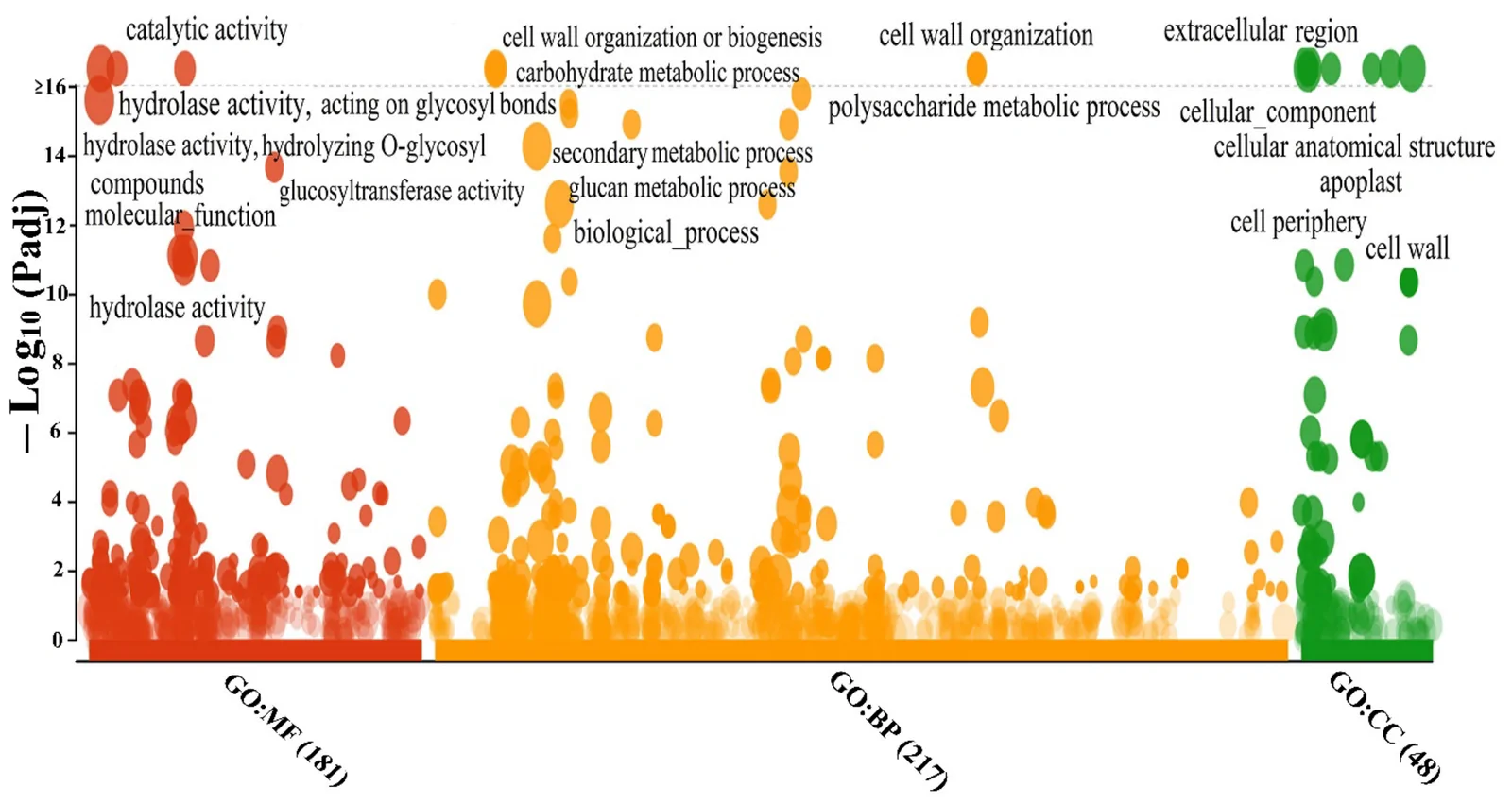
Gene Ontology (GO) enrichment analysis of down-regulated differentially expressed genes (DEGs) in the ‘Mona’ cultivar using the g: Profiler database Benjamini–Hochberg FDR test with a *p*-value ≤ 0.05 as a threshold. The GO enrichment bubble chart includes the three GO categories: Molecular Function (MF), Biological Process (BP), and Cellular Component (CC).

In response to drought, adenyl ribonucleotide binding (GO:0032559), carbohydrate derivative binding (GO:0097367) and glutathione transferase activity (GO:0004364) were the top molecular functions associated with genes exhibiting elevated expression in the drought-tolerant genotype (Figure 4). Moreover, the most significant biological processes identified in tolerant wild barley included regulation of cellular respiration (GO:0043457), regulation of generation of precursor metabolites and energy (GO:0043467), glutathione metabolic process (GO:0006749), protein phosphorylation (GO:0006468), response to stress (GO:0006950), and defense response (GO:0006952) (Figure 4). Protein serine/threonine phosphatase activity (GO:0004722), response to abiotic stimulus (GO:0009628), response to heat (GO:0009408), and response to temperature stimulus (GO:0009266) were the top two functional groups enriched among genes with increased transcript abundance in the sensitive genotype (Figure 5). Genes with reduced expression in the tolerant genotype were significantly enriched for catalytic activity (GO:0003824), biological process (GO:0008150), and plastid and chloroplast (GO:0009536 and GO:009507) in response to drought (Figure 6). Cell wall organization or biogenesis (GO:0071554), catalytic activity (GO:0003824), and extracellular region (GO:0005576) were the top three processes among genes with decreased expression levels in the ‘Mona’ cultivar (Figure 7). Collectively, these enriched GO categories could indicate that drought tolerance in wild barley is associated not with a single protective mechanism but rather with a coordinated reprogramming of cellular organization, signaling networks, ion transport, reactive oxygen species ROS detoxification, and stress-responsive gene regulation.

Kyoto Encyclopedia of Genes and Genomes (KEGG) analysis of the four DEG groups was carried out to determine their potential roles in drought adaptation. KEGG pathway enrichment analysis showed that 507 up-regulated DEGs of drought-tolerant genotype were assigned to 270 pathways, whereas 459 down-regulated DEGs were mapped to 265 pathways. Among the enriched pathways, eight major categories included metabolic pathways (KO 01100), biosynthesis of secondary metabolites (KO 01110), carbon metabolism (KO 01200), biosynthesis of amino acids (KO 01230), mitogen-activated protein kinase (MAPK) signaling pathway (KO 04016), plant hormone signal transduction (KO 04075), glycerophospholipid metabolism (00564), and phenylpropanoid biosynthesis (KO 00940) (Figure S5a,b).

In the drought-sensitive cultivar, we mapped 840 genes to the reference KEGG pathways. Among those, 273 up-regulated genes were assigned to 183 KEGG pathways, while 567 down-regulated genes were assigned to 272 KEGG pathways. The top enriched pathways for up-regulated genes in the sensitive cultivar in response to salt stress included metabolic (KO 1100), biosynthesis of secondary metabolites (KO 01110), starch and sucrose metabolism (KO 00500), galactose metabolism (00052) and biosynthesis of amino acids (KO 01230). The top five pathways of down-regulated genes in the sensitive cultivar were categorized as: metabolic pathways (KO 1100), biosynthesis of secondary metabolites (KO 01110), biosynthesis of amino acids (KO 01230), flavonoid biosynthesis (KO 00941), oxidative phosphorylation (KO 00190), and photosynthesis (KO 00195) (Figure S5c,d). Plant hormone signal transduction pathways and flavonoid biosynthesis are shown in Supplementary Figures S6 and S7, respectively. The simultaneous enrichment of ABA signaling, MAPK signaling, antioxidant metabolism, and osmotic adjustment pathways suggests the existence of a tightly interconnected stress-response network that enables the tolerant genotype to rapidly perceive drought stress and activate downstream adaptive responses [19,20].

### 2.7. Candidate Genes for Drought Tolerance

Further comparing the DEGs identified under control and drought stress conditions between wild and cultivated genotypes could provide additional insights into the drought resilience of wild barley. Tables S3–S9 highlight key functionally assigned candidate genes linked to drought tolerance in barley, emphasizing the statistical significance of their expression differences. Among the identified DEGs, the six most significant groups include signaling elements, ion transporters, regulatory proteins (transcription factors and kinases), genes involved in ROS scavenging, genes involved in phytohormone biosynthesis, and genes involved in osmotic homeostasis and accumulation of compatible solutes and are described in detail below.

#### 2.7.1. Abscisic Acid (ABA)-Independent Signaling

Calcium signaling components, including caleosins, calmodulins, and protein kinases, were significantly up-regulated in response to drought stress in the wild genotype. Notably, we detected expression of two caleosin transcripts exclusively in the wild-tolerant barley. Several calcium-binding protein genes (*HvCML12*, *HvCML21*, *HvCML22*, and *HvCML31*) were uniquely and strongly upregulated in the tolerant plants. Both barley genotypes showed significantly elevated expression of the calcium-binding protein genes (*HvCML15* and *HvCML27*) in response to drought. *HvCML10* was upregulated in the tolerant genotype, whereas it was downregulated in the sensitive genotype (Table S3). The transcript of the calmodulin-like protein was only increased in the tolerant genotype. The transcripts of calcium-transporting ATPase (PMCA) were reduced under drought stress in the drought-sensitive genotype, whereas a strong increase (log_2_FC = 5.9) was observed in the tolerant genotype. The expression of *HvAnn1*, another calcium-binding protein gene, was highly altered in the wild genotype. Moreover, the calcium-binding Copine C-terminal domain-containing protein showed unique and strong upregulation (log_2_FC = 9.46) in the tolerant genotype under drought stress.

**Figure 8:**
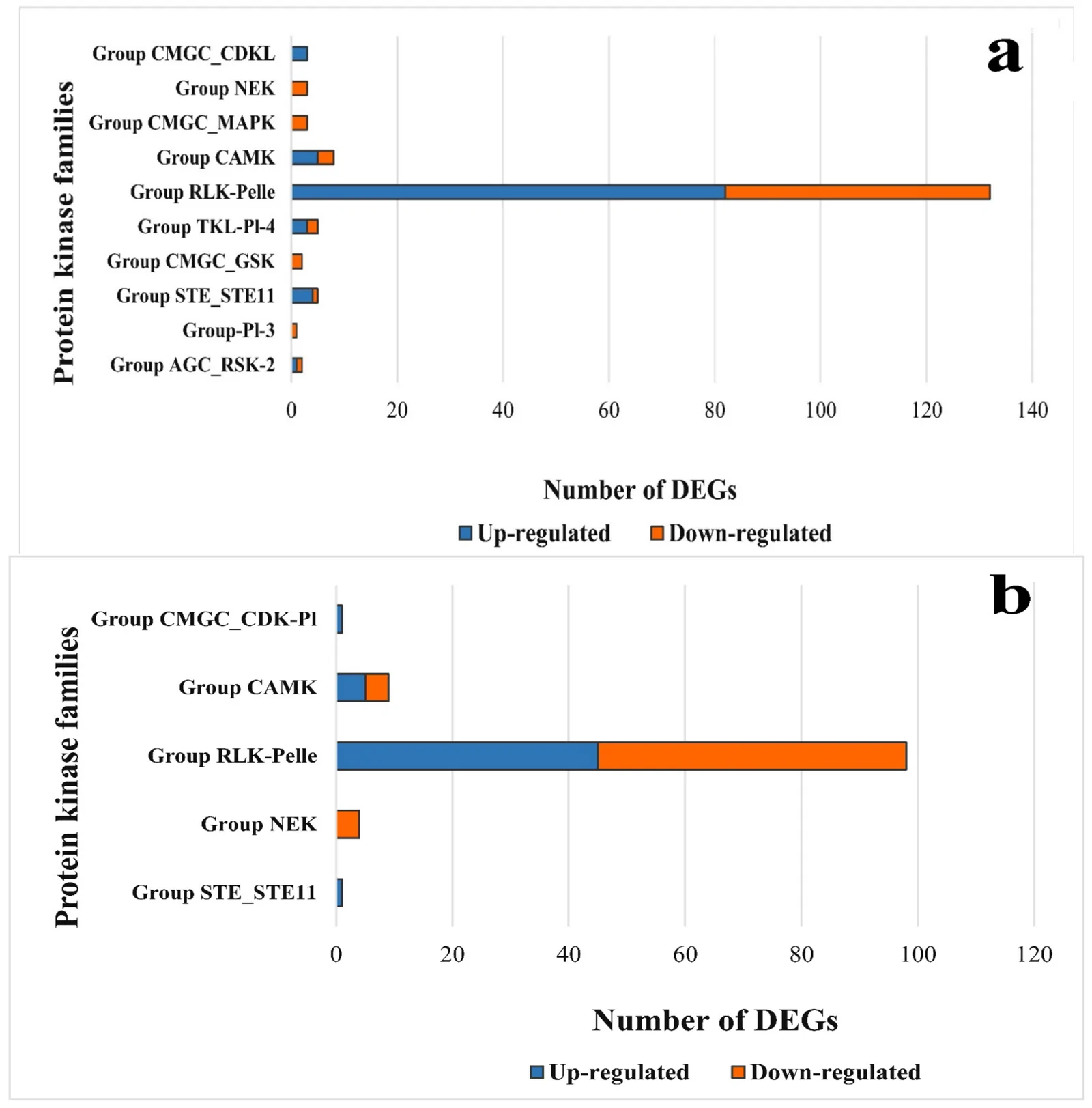
Differentially expressed kinase genes [protein kinases (PKs)] detected in the (**a**) drought-tolerant genotype and (**b**) drought-sensitive cultivar (“Mona”). Number of up-regulated (blue bar) or down-regulated (orange bar) PK-encoding genes across protein kinase families.

Analysis of protein kinases (PKs) in the tolerant wild barley revealed 98 upregulated and 66 downregulated PK-encoding genes under drought stress. These PKs were classified into the following families: AGC, STE, CRLK-Pelle, CMGC, TKL, PI3, NEK, and CAMK. Among the protein kinases RLK-Pelle family was the largest family, containing 82 up-regulated genes found in wild barley (Figure 8a). This family comprised 34 kinase types, including RLK-Pelle-DLSV, RLK-Pelle-L-LEC, RLK-Pelle-WAK, and RLK-Pelle-SD-2 b. In addition, 21 and 18 protein kinases belonged to RLK-Pelle-LRR and RLK-Pelle-RLCK, respectively. In contrast, 46 PKs belonging to the RLK-Pelle family and 7 PKs belonging to families (CAMK, CMGC, and STE) were differentially upregulated under drought stress in the ‘Mona’ cultivar (Figure 8b). Two categories of plant-specific *TLK* and *STE* genes were significantly upregulated only in the wild genotype in response to drought stress, whereas in ‘Mona’, only one *STE* gene was upregulated, and no *TLK* members were detected. Furthermore, a unique upregulation of AGC_RSK-2 (Ribosomal S6 Kinase 2) was observed exclusively in the wild barley (Table S4). The most abundant PK families were RLK-Pelle, and CAMK, which were common to both genotypes. In contrast, three MAPK genes belonging to the CMGC family were upregulated only in the wild barley. The expression of AGC_RSK-2, PI3, TLK, CMGC/MAPK, and CAMK_CDPK kinases was altered exclusively in the wild tolerant genotype under drought stress.

#### 2.7.2. Genes Involved in Phytohormone Biosynthesis

The *HvPYR/PYL* gene was down-regulated in the sensitive genotype but up-regulated in the tolerant one. Another gene in this group, *HvPP2C*, showed significantly higher expression in the sensitive cultivar. In the sensitive cultivar, genes associated with the auxin signaling pathway—including the auxin-responsive protein IAA, DELLA proteins, and phytochrome-interacting factor involved in gibberellin biosynthesis—exhibited markedly reduced expression under drought conditions, whereas no significant differential expression was observed in the tolerant genotype. In contrast, in the tolerant barley, *HvJAZ* genes were up-regulated in the jasmonic acid signal transduction pathway (Figure S6a,b and Table S5).

#### 2.7.3. Transcription Factors

**Figure 9:**
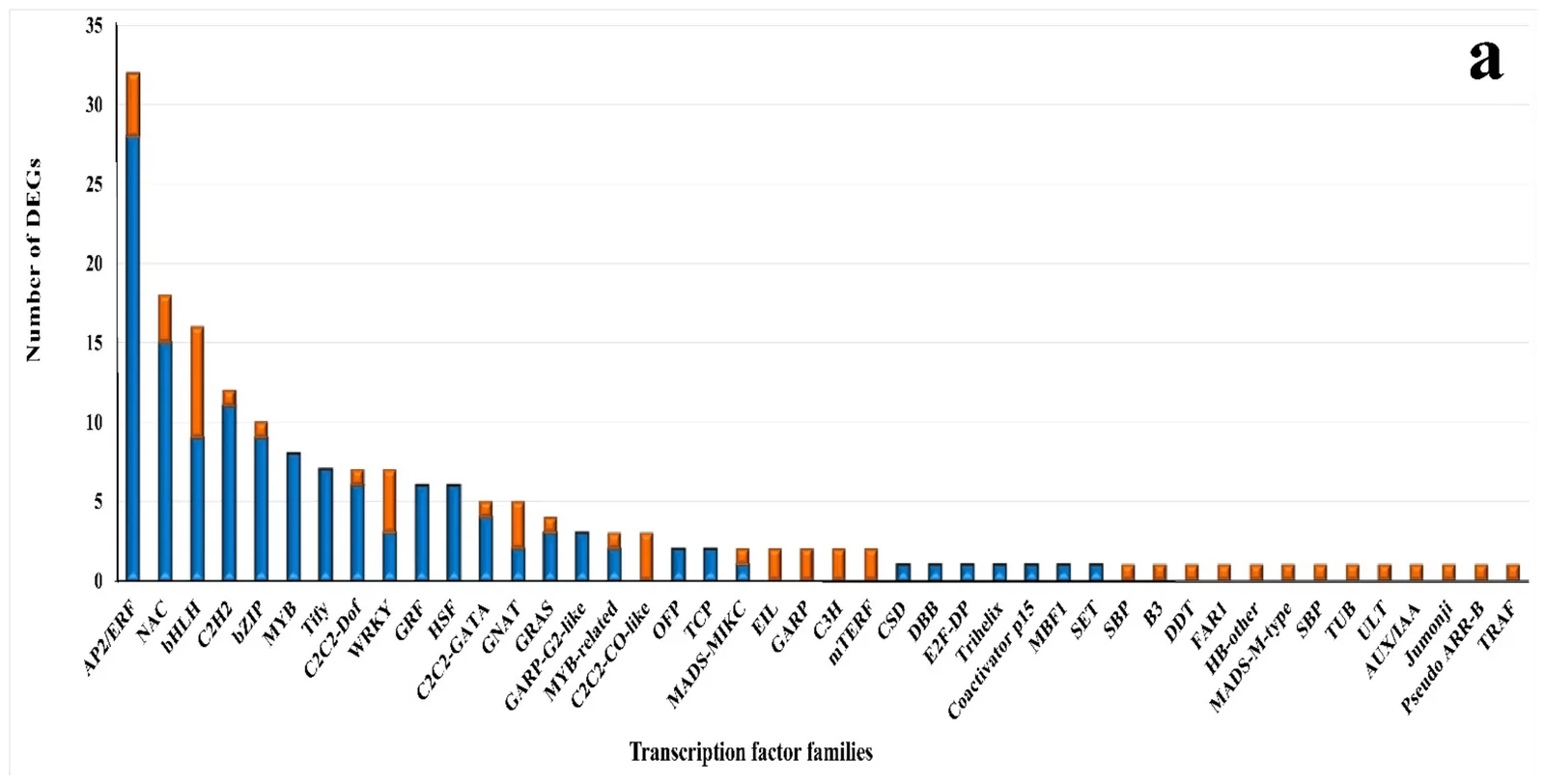

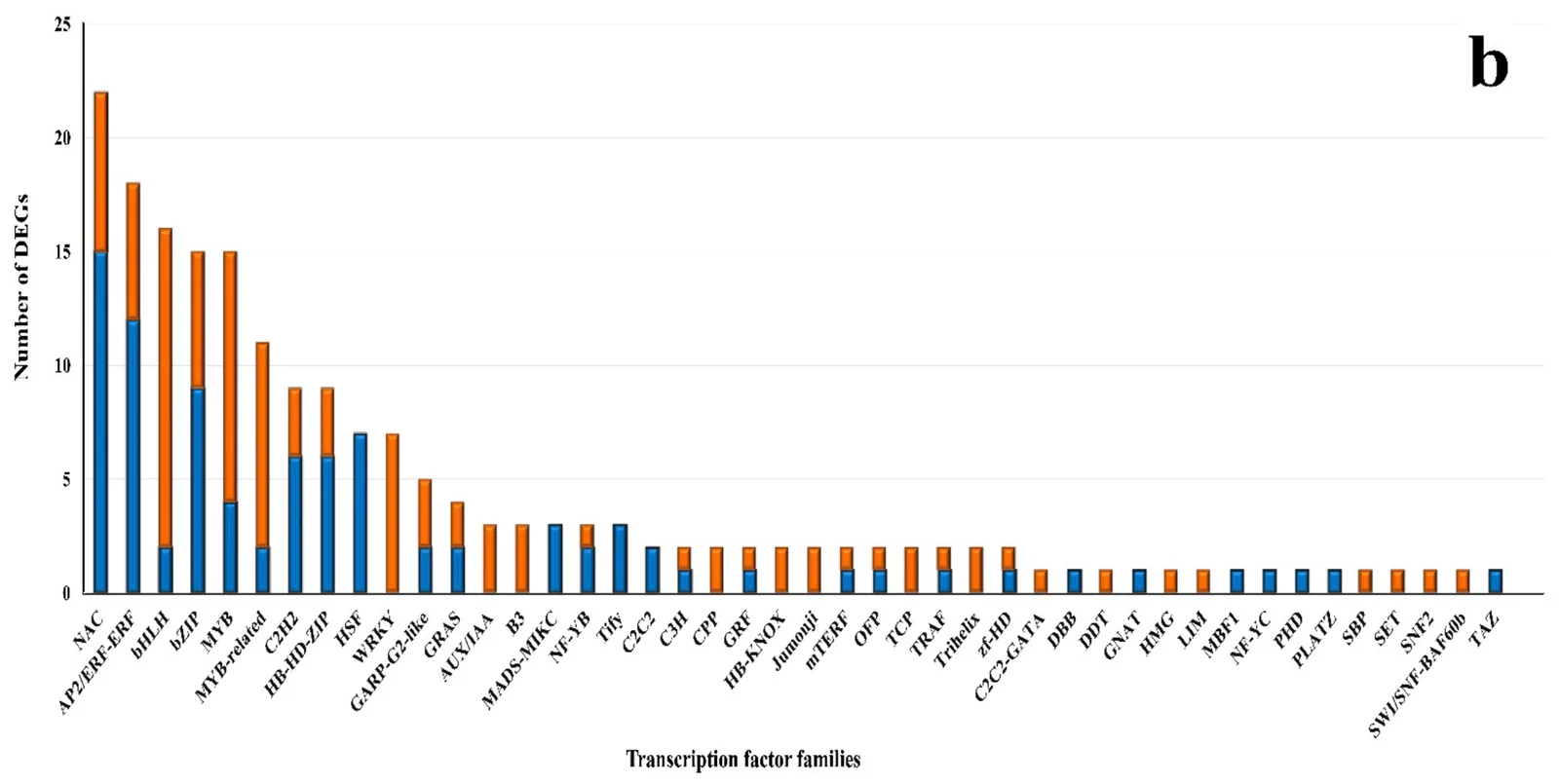
Differentially expressed Transcription factors (TFs) identified in the (**a**) drought-tolerant genotype and (**b**) drought-sensitive cultivar (“Mona”). Number of up-regulated (blue bar) or down-regulated (orange bar) TF-encoding genes across transcription factor families.

In the drought-tolerant genotype, a total of 44 transcription factor (TF) families comprising 189 genes were identified using the iTAK pipeline. Among these, 136 genes were upregulated and 53 were downregulated in response to drought stress. In this study, AP2/ERF, NAC, C2H2, bHLH, bZIP, MYB, TIFY, C2C2-Dof, GRF, HSF, and WRKY families were highly expressed under drought stress compared with control conditions. In contrast, the TF families bHLH, AP2/ERF, WRKY, C2C2-CO-like, GNAT, and NAC were downregulated under drought conditions (Figure 9a).

In the ‘Mona’ cultivar, a greater number of differentially expressed TFs showed a trend toward decreased expression (125 TFs in total: 24 upregulated and 101 downregulated, belonging to 45 TF families). NAC, AP2-ERF, bZIP, C2H2, HSF, and HB-HD-ZIP families were mainly upregulated, while bHLH, AP2/ERF, MYB, WRKY, AUX/IAA, B3, and GARP-G2-LiKe followed a down-regulation pattern in this cultivar (Figure 9b and Table S6). In the present study, drought stress-induced genes encoding Trihelix, WRKY, TCP, Coactivator p15, CDS, and E2F-DP proteins exhibited increased expression in the tolerant genotype, whereas no expression changes were observed in the sensitive cultivar. *AP2/ERF* TF-related genes showed more than double the expression increase in the wild-tolerant genotype compared with the sensitive ‘Mona’ cultivar. The *HvNAC* family was the common TF family induced by drought stress in the two barley genotypes. Conversely, the reduction in expression of the bHLH TFs family under drought stress in the sensitive cultivar was twice that of the tolerant genotype. Additionally, drought stress decreased expression of genes encoding MYP, GARP-G2-Like, OFP, TCP, Trihelix, and SET proteins.

#### 2.7.4. Ion Transporters and Channels

Comparative transcriptome analysis of ion transporters and ion channels in wild (tolerant) and cultivated (sensitive) barleys under control and drought-stress conditions revealed several key genes involved in the regulation of ion transport and ion channels. The ATP-binding cassette (ABC) transporters, a large family of membrane-localized proteins, exhibited distinct expression patterns between the two genotypes. *HvABCG32* and *MDR-like ABC* transporter genes were uniquely up-regulated in the tolerant genotype under drought stress (Table S6). The MDR-like ABC transporter (HORVU.MOREX.r3.6HG0558970) showed the highest induction level with a log_2_FC > 7.2 in wild-tolerant barley. Similarly, *HvABCG32* expression increased 3.5-fold in the tolerant genotype, while no significant induction was detected in the cultivated barley. In contrast, *HvABCB19* and *HvABCG16* were specifically downregulated in the sensitive cultivar. The expression of *HvABCC3*, *HvABCI17*, *HvABCC18*, and *HvABCG44* genes was significantly altered in both barley genotypes (Table S7).

A total of 10 Major Facilitator Superfamily (MFS)-related genes exhibited differential expression, with six specifically upregulated in the tolerant genotype, with four upregulated in both genotypes. However, the magnitude of upregulation in the tolerant genotype was significantly higher than in the sensitive cultivar.

The sulfate transporter gene HvSultr3;4 (*HvSPDT*) showed an approximately 2.8-fold higher expression in the wild-tolerant barley compared to the cultivated one under drought stress. Similarly, the calcium-transporting ATPase transcripts were induced 5.9-fold in the tolerant barley, while no significant changes were observed in the ‘Mona’ cultivar. The potassium channel SKOR was upregulated in both genotypes under drought conditions. Furthermore, the potassium transporter showed a strong induction (log_2_FC = 5.7) in the tolerant barley but remained unchanged in the sensitive cultivar. The potassium transporter 16-related gene was upregulated in both genotypes.

Genes encoding Na^+^/Ca^2+^ exchangers (log_2_FC = 10.34 and 4.01), arsenical pump-driving ATPase (log_2_FC = 7.1), auxin efflux carrier component 5B-related protein, and plasma membrane ATPase were significantly upregulated by drought stress only in the tolerant barley. Additionally, dirigent proteins were induced under drought stress in the tolerant genotype, whereas they showed downregulation in the sensitive cultivar (Table S7). Together, these observations revealed that genes encoding ion transporters and channel proteins are predominantly expressed in drought-tolerant barley, implicating them in the maintenance of ion homeostasis and the augmentation of drought tolerance.

#### 2.7.5. Genes Encoding Reactive Oxygen Species Scavenging Proteins

Comparative transcriptome profiling of ROS inactivators in wild (tolerant) and cultivated (sensitive) barley genotypes under control and drought-stress conditions revealed multiple key genes associated with ROS detoxification mechanisms. Under drought stress, genes associated with the flavonoid biosynthesis and flavone/flavonol biosynthesis pathways—including Flavonoid 3′-monooxygenase (CYP75B1), Flavonoid 3′-monooxygenase (CYP75A), Shikimate O-hydroxycinnamoyl transferase (HCT, EC:2.3.1.133), and Flavonol-3-O-glucoside L-rhamnosyltransferase (EC:2.4.1.159)—were significantly upregulated in the wild barley (Figure S7a).

A marked reduction in expression of flavonoid biosynthesis pathway genes, including chalcone synthase (EC:2.3.1.74), chalcone-flavonone isomerase (E5.5.1.6), CYP75B4, CYP75B3, anthocyanidin synthase (EC:1.14.20.4), anthocyanidin reductase (EC:1.3.1.77), and bifunctional dihydroflavonol 4-reductase/flavanone 4-reductase (EC:1.1.1.219 1.1.1.234), was observed in the Mona cultivar (Figure S7b). The greatest reduction was detected in the anthocyanidin synthase gene (Log_2_FC = −23). The flavonol synthase (EC:1.14.20.6) gene involved in this pathway exhibited increased expression in the wild barley, while its expression was reduced in the Mona cultivar (Table S8). Further comparative transcriptome analysis of antioxidant-related genes between the two genotypes under control and drought conditions identified several transcripts contributing to drought tolerance. In response to drought stress, the expression of 17 glutathione S-transferase genes and duplicate ferredoxin (*HvFdx3*) genes was significantly induced in wild barley (Table S8). Moreover, genes encoding enzymatic antioxidants, including *HvPOD* were strongly up-regulated in response to drought stress in the wild barley genotype. Furthermore, the tolerant genotype exhibited significant up-regulation of many glycosyl transferase genes, while only a few such genes were up-regulated in the sensitive cultivar. Taken together, these findings indicated that increased activation of flavonoid biosynthesis, antioxidant enzymes, and glycosyl transferases drove efficient ROS detoxification and contributed to superior drought tolerance in the wild barley genotype.

#### 2.7.6. Regulation of Genes Involved in Osmotic Homeostasis and Accumulation of Compatible Solutes

Under drought stress conditions, six genes encoding LEA (late embryogenesis abundant) proteins were activated in the wild barley. This up-regulation suggested a potential role for LEA proteins in enhancing drought tolerance mechanisms, possibly through cellular protection and water retention during stress exposure. In the Mona cultivar, transcriptome analysis under drought stress revealed differential expression patterns among LEA protein-related genes. Specifically, only two LEA-associated genes were up-regulated. In contrast, eight LEA-related genes exhibited down-regulation, suggesting a possible impairment or suppression of stress-responsive pathways in this sensitive cultivar. Both barley genotypes showed significant up-regulation of the *Dhn1*, *Dhn7*, *HvP5cr3*, and *HvP5CSB* genes under drought stress, while *Dhn10* (Log_2_FC = 7.6) was up-regulated exclusively in the tolerant genotype.

Protective protein genes were activated in both the wild and cultivated barley genotypes under drought stress, but they differed markedly in the number of genes induced and the magnitude of their expression. Under drought stress, this study found significantly higher expression of genes encoding small heat shock proteins (sHSPs), HSP70 and HSP90 family members, and chaperones such as peptidyl-prolyl isomerases, 17 kDa, 18 kDa, DnaK, DnaJ, and HtpG, compared to control conditions (Table S9).

In contrast, aquaporin genes were significantly downregulated in the drought-sensitive cultivar. Interestingly, none of the aquaporin-related genes showed differential expression in the drought-tolerant genotype. Within the aquaporin family, *HvTIP1;1* exhibited the most pronounced reduction (Log_2_FC = −9.8) in the Mona cultivar under drought stress. Following the PIP subfamily, NIP aquaporins showed the second highest level of downregulation (Log_2_FC = −4.4). These results suggested a potential role for *HvTIP1;1* in regulating water transport and highlighted its sensitivity to environmental stress in the drought-sensitive cultivar. Additionally, Peptidase S59 was expressed 3.5-fold higher in the wild-tolerant genotype compared with the sensitive cultivar (Table S9).

### 2.8. Validation of Candidate Genes Through Real-Time Expression Analysis

**Figure 10:**
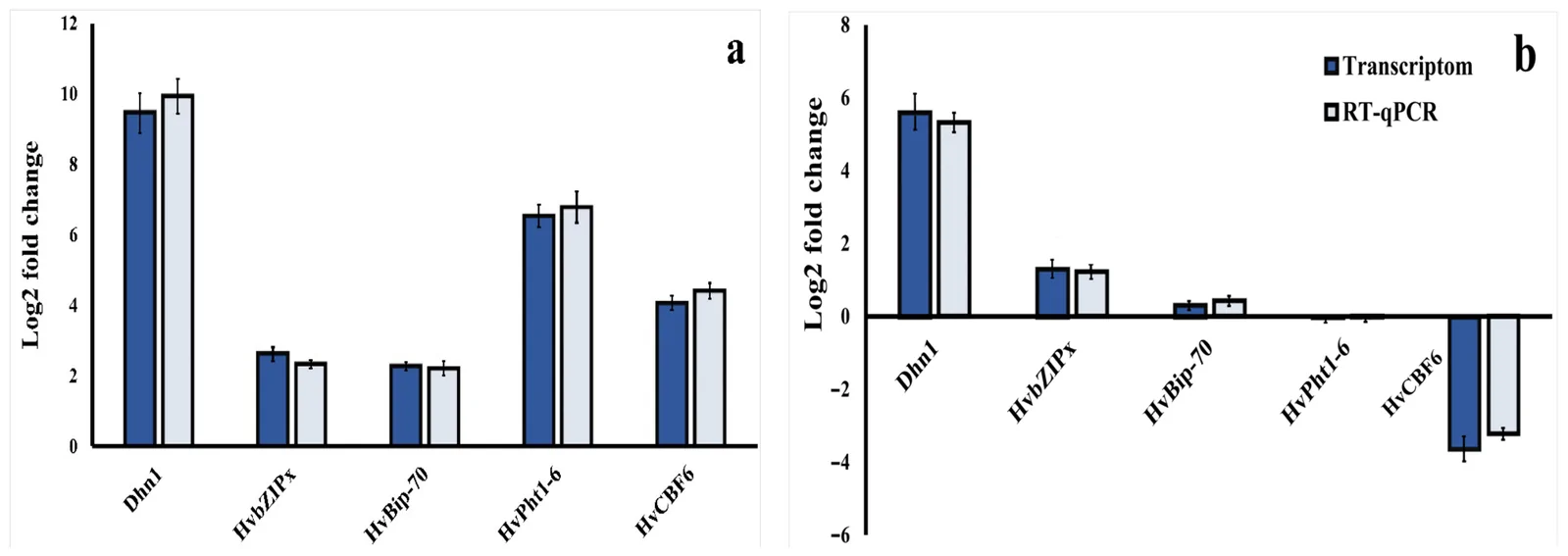
Comparison of RT-qPCR and RNAseq gene expression results using five genes (*Dhn1*, *HvbZIPx*, *HvBip-70*, *HvPht1-6* and *HvCBF6*): (**a**) drought-tolerant wild genotype and (**b**) drought-sensitive cultivar ‘Mona’. Black bars represent RNA-seq data, and grey bars represent RT-qPCR data. Bars represent means values ± standard error (SE) for transcriptome (RNA-seq) data and RT-qPCR validation.

Real-time PCR analysis was conducted to assess the expression patterns of five selected genes (including *HvDhn1*, *HvbZIPx*, *HvBip-70*, *HvPht1-6* and *HvCBF6*, detailed in Table 3) and to validate the accuracy of the sequencing-based expression results. The expression profiles obtained for these candidate genes showed a consistent trend between RT-qPCR data and transcriptome analysis under drought stress conditions (Figure 10). In the drought-tolerant wild genotype, five stress-responsive genes were up-regulated. By contrast, three of these five genes showed either very low induction or—as observed for *HvCBF6*—down-regulation in the drought-sensitive cultivar “Mona”.

## 3. Discussion

### 3.1. Plant Growth and Physiological Attributes

The contrasting responses of wild and cultivated barley genotypes indicated that drought tolerance was governed by an integrated network of regulatory, physiological, antioxidant, and hormonal mechanisms. Wild genotypes appeared to maintain cellular homeostasis more effectively under water deficit, suggesting that their drought adaptation involved more coordinated structural and biochemical adjustments. Similar patterns have also been reported in Iranian *Hordeum spontaneum*, where wild genotypes exhibited stronger structural resilience and more efficient biochemical coordination under drought. The greater stability of photosynthetic pigments in wild germplasm further implied that these genotypes preserved chloroplast integrity and sustained photosynthetic efficiency under stress. Previous observations on Iranian wild barley have described comparable pigment stability, but these results provide direct evidence for this trait under drought. This stability was consistent with their stronger antioxidant activation and enhanced ROS-scavenging capacity, which together mitigated oxidative damage before it affected the photosynthetic apparatus. The elevated accumulation of phenolics, flavonoids, and proline in tolerant lines reflected a multilayered defense system that reinforces cellular protection and contributed to sustained metabolic performance during drought. Although earlier studies have noted similar trends, the current findings specifically highlight these responses in the examined genotypes. Studies on Iranian wild barley confirmed that high levels of phenolics, flavonoids, and osmolytes were conserved physiological features of wild germplasm, contributing to their superior drought resilience. Such physiological consistency suggests that these traits may contribute to drought resilience [16,21,22]. Hormonal responses under drought revealed distinct regulatory strategies between tolerant and sensitive barley genotypes. Abscisic acid increased significantly in both genotypes, indicating drought perception and ABA-mediated signaling. The more stable integration of ABA changes in the tolerant genotype suggests a better-coordinated hormonal adjustment, whereas the sensitive genotype showed weaker downstream responses. Auxin dynamics further highlighted this divergence: the tolerant genotype maintained or slightly enhanced IAA levels under drought, whereas the sensitive genotype exhibited a marked decline, reflecting impaired growth regulation. Gibberellin levels remained stable in the tolerant line but decreased substantially in the sensitive cultivar, indicating disrupted hormonal homeostasis. These observations are consistent with previous studies reporting that drought-tolerant or wild barley genotypes typically exhibited stronger ABA signaling, better maintenance of auxin homeostasis, and more controlled reductions in gibberellins under water deficit. Studies on Iranian *Hordeum spontaneum* also emphasized this coordinated hormonal regulation—particularly balanced ABA, IAA, and GA dynamics—as a defining feature of wild barley. Earlier research similarly showed that sensitive cultivars often displayed sharp declines in IAA and GA and failed to integrate ABA induction into an effective downstream regulatory framework, resulting in impaired growth regulation and weaker stress adaptation. The convergence of these findings across multiple studies underscores that coordinated hormonal regulation—particularly balanced ABA, IAA, and GA dynamics—is a conserved feature of drought-tolerant barley germplasm [15,16]. The oxidative stress indicator further strengthened the physiological distinction between the genotypes. Under drought, the tolerant line exhibited markedly lower accumulation of MDA and H_2_O_2_, reflecting its superior capacity to restrict lipid peroxidation and prevent excessive ROS buildup. In contrast, the sensitive cultivar showed pronounced increases in both markers, indicating severe oxidative injury and insufficient activation of detoxification pathways. The substantial enhancement of DPPH activity in the tolerant genotype demonstrated a more efficient antioxidant machinery capable of rapidly neutralizing reactive oxygen species. Although the sensitive genotype displayed a moderate rise in DPPH activity, this response appeared insufficient relative to its higher oxidative load. Previous studies have described similar MDA, H_2_O_2_ and antioxidant trends in barley [23]. Comparative studies on Iranian wild barley confirmed that enhanced ROS detoxification and improved membrane stability were consistent physiological traits of wild germplasm [16].

Taken together, these hormonal, antioxidant, and physiological patterns demonstrated that drought tolerance in wild barley arose from a highly coordinated regulatory system in which ABA induction is complemented by maintaining auxin and gibberellin balance, enhanced ROS detoxification, and structural resilience. In contrast, the sensitive genotype—despite showing an ABA increase—exhibited sharp declines in IAA and GA, weak antioxidant activation, and substantial oxidative damage. Collectively, these findings indicated that drought tolerance in wild barley was governed by an integrated network involving hormonal regulation, osmotic adjustment, antioxidant defense, and structural stability. Previous studies have reported similar mechanisms in wild barley germplasm [16]. These patterns suggest the potential value of wild barley as a genetic resource for improving drought resilience.

#### Elemental Analysis Using ICP-MS

The present study revealed distinct differences in mineral accumulation and partitioning between drought-sensitive and drought-tolerant genotypes. In the sensitive genotype, aluminium concentration increased sharply in grains (+113%), reflecting substantially higher Al levels in reproductive tissues. In contrast, the tolerant genotype exhibited reduced aluminium concentrations in both grains and leaves, suggesting the more effective retention or exclusion of Al within vegetative tissues. These patterns were consistent with previous observation [22,24,25], which reported that drought-tolerant wheat and barley genotypes generally maintain lover tissue levels of harmful elements. The substantial Al accumulation in the sensitive genotype may reflect less effective detoxification or exclusion processes. This pattern may suggest that cultivated barley has weaker ion-exclusion capacity than the tolerant genotype. Barium concentrations declined in both grains and leaves of the two genotypes, with a more pronounced reduction in leaves. This suggests that foliar tissues played a major role in limiting Ba accumulation under drought. Nawaz et al. [26] and Quagliata et al. [25] reported similar trends, highlighting the importance of foliar tissues in restricting the accumulation of heavy or non-essential ions. The stronger decline in Ba in the tolerant genotype may indicate more effective regulation of non-essential elements.

Copper accumulation exhibited genotype-specific responses: the sensitive genotype showed no significant variation, whereas the tolerant genotype displayed a threefold increase in grain copper concentration. This indicates higher Cu levels in the reproductive tissues of the tolerant genotype, which may support grain quality and antioxidant capacity. These results align with Sallam et al. [27] and Quagliata et al. [25], who demonstrated that tolerant wheat and barley genotypes maintain higher micronutrient concentrations in reproductive organs under drought stress. The tolerant genotype’s ability to maintain elevated Cu concentrations under drought may reflect more effective regulation of micronutrient-related pathways, including those linked to flavonoid metabolism. Calcium exhibited contrasting trends between the two barley genotypes: grain calcium increased under drought in the sensitive genotype but remained stable in the tolerant one. In leaves, calcium decreased in the sensitive genotype but increased in the tolerant genotype. These differences suggest more stable Ca regulation in the tolerant line, which may support membrane stability under drought. These findings corroborate Nawaz et al. [26] and Quagliata et al. [25], who highlighted calcium as a key factor in drought tolerance. Given the central role of Ca^2+^ in stress signaling, the divergent Ca patterns may indicate differences in Ca^2+^-related signaling responses between the genotypes, potentially contributing to variation in hormonal and antioxidant regulation under drought. Potassium and phosphorus concentrations decreased in both grains and leaves of the sensitive genotype, whereas drought increased them in the tolerant genotype. The ability of the tolerant genotype to retain or enhance these nutrients, may support osmotic adjustment and metabolic activity during drought. Our results are in agreement with Nawaz et al. [26] and Quagliata et al. [25], who reported enhanced nutrient retention in drought-tolerant barley and other cereals. The severe depletion of K (−45%) and P (−60%) in leaves of the sensitive genotype may indicate greater disruption of nutrient regulation, potentially affecting stomatal behaviour and energy metabolism. In contrast, the tolerant genotype’s ability to increase K and P concentrations under drought may contribute to more stable turgor pressure and a coordinated physiological response. Iron concentrations increased in both genotypes, with more pronounced accumulation in leaf tissues. The stronger Fe induction in the tolerant genotype (+92%) suggests Fe-associated physiological processes under drought, which may support photosynthetic and antioxidant functions. Kosová et al. [22], Quagliata et al. [25], and Sallam et al. [27] similarly observed enhanced iron accumulation in tolerant wheat and barley genotypes. Elevated Fe content was accompanied by markedly enhanced POX and CAT activities, as iron served as a critical co-factor for chlorophyll biosynthesis, electron transport complexes, and ROS-scavenging enzymes. In contrast, the cultivated genotype exhibited only a modest increase in Fe, suggesting comparatively weaker activation of Fe-associated antioxidant and metabolic pathways.

Manganese levels increased significantly in the tolerant genotype but remained unchanged in the sensitive one. This supports its role in activating antioxidant enzymes, consistent with Liu et al. [28], who emphasized manganese as a critical factor in enzymatic defense under drought. The strong Mn induction in the tolerant genotype (+83% in grains and +53% in leaves) may reflect more effective activation of Mn-associated antioxidant responses, whereas the lack of Mn change in the sensitive genotype may indicate less responsive stress-related regulatory processes. Magnesium and strontium concentrations in grains of the sensitive genotype showed notable reductions under drought stress, while the tolerant genotype maintained stable levels of both elements, consistent with Kosová et al. [22] and Quagliata et al. [25], who emphasized the critical role of magnesium homeostasis in sustaining photosynthetic efficiency. Stable Mg levels in the tolerant genotype may help maintain chloroplast function and metabolic stability, whereas Mg depletion in the sensitive genotype (−37% in leaves) may contribute to pigment loss, ROS accumulation, and reduced antioxidant activity.

Zinc concentrations decreased in both leaves and grains of the sensitive genotype, while the tolerant genotype exhibited higher zinc accumulation in grains. Zn depletion in cultivated barley (−45% in leaves) may help explain reduced activity of stress-related regulatory and antioxidant processes, given Zn’s role in transcription factor function (MYB, bZIP). Our findings are consistent with Amini et al. [29] and Kumar et al. [30], who demonstrated that zinc enrichment enhances seed vigour and drought resilience in wheat and barley. Finally, molybdenum, sodium, and sulfur concentrations increased in the tolerant genotype’s grains. Mo supported nitrogen metabolism and nitrate reduction, Na supported osmotic adjustment, and S was essential for protein synthesis and defense compounds such as glutathione. These results align with Nawaz et al. [26] and Sallam et al. [27], who reported improved retention of these elements in drought-tolerant cereals. The coordinated increase in Mo (+43%), Na (+49%), and S (+14%) in the tolerant genotype may reflect more effective nutrient-related regulatory responses. Although direct ionomic measurements of Iranian wild barley under drought stress are not available, numerous physiological and molecular studies on *Hordeum spontaneum* suggest that wild germplasm retains stress-responsive regulatory features that contribute to more stable nutrient patterns under adverse conditions.

Overall, the tolerant genotype effectively maintained or enhanced essential mineral nutrients (Ca, K, P, Mn, Mg, Zn, Mo, S, Fe) while showing lower tissue level of toxic or non-essential elements (Al, Ba, Sr). This pattern may help support ionic balance, photosynthetic performance, and grain quality under drought stress. Our findings strongly agree with previous studies in wheat, barley, and other cereals, confirming that mineral nutrition plays a pivotal role in drought tolerance [25,26,27,28]. Importantly, these mineral patterns may help explain differences in hormonal and antioxidant responses between genotypes; the tolerant genotype appears capable of sustaining more effective ABA-related and flavonoid-associated stress responses. In cultivated barley, depletion of key nutrients (K, P, Ca, Mg, Zn) and higher levels of toxic ions (Al) may reflect less effective nutrient uptake and reduced coordination among stress-related pathways.

### 3.2. Transcriptome Analysis

The primary objective of this study was to evaluate transcriptional changes and identify key genes associated with drought tolerance in barley. Drought tolerance in plants involves complex interactions among multiple signaling and regulatory pathways. In this study, a high level of concordance between RNA-Seq and qRT-PCR analyses results was observed, confirming the reliability of the detected DEGs associated with drought adaptation and validating their association with it. The use of identical RNA samples for both assays minimized potential variation arising from biological sampling, further strengthening the reliability of these findings [31].

The number of DEGs identified under drought stress was significantly higher than under normal conditions in both barley genotypes. These findings are consistent with previous transcriptome studies in barley under drought [31], salt [13], and heat stress [32]. A key point is that both salinity [13] and heat stress [32] studies were conducted on Iranian wild barley germplasm, with each reporting extensive transcriptional reprogramming characterized by a high proportion of up-regulated stress-responsive genes. The similarity between our drought-induced transcriptomic patterns and those observed under salinity and heat in wild barley suggests that the strong transcriptional activation detected in the tolerant genotype reflects a broader, conserved regulatory behaviour of Iranian wild germplasm across multiple stress conditions. Notably, the drought-tolerant genotype in our study exhibited more DEGs than the drought-sensitive cultivar, indicating that the wild subspecies mounted a stronger transcriptional response to drought stress. Importantly, the tolerant genotype not only showed a higher total number of DEGs but also a markedly greater proportion of up-regulated genes, reflecting stronger activation of stress-responsive pathways under drought. While this extensive up-regulation may indicate more effective stress signaling, attributing it to ancestral regulatory retention would require broader evidence. In contrast, the cultivated genotype exhibited fewer up-regulated DEGs and weaker induction of key regulatory families, suggesting a less responsive transcriptional adjustment to drought accumulation, ion homeostasis, and flavonoid biosynthesis. Furthermore, our transcriptome results align closely with physiological observations, reinforcing the conclusion that drought-tolerant barley exhibits more robust activation of stress-responsive genes than its sensitive counterpart. Together, these findings demonstrate that the stronger transcriptional activation observed in wild barley is biologically meaningful and directly underpins its superior drought tolerance.

### 3.3. Genotype × Treatment Interaction Suggests Divergent Drought-Response Mechanisms

The genotype × treatment interaction analysis provides additional perspective on how drought responses may differ between barley genotypes. The distribution of interaction-significant genes suggests that drought-induced transcriptional changes may include genotype-specific induction, shared responses with varying intensities, and cases of opposite regulation. These patterns broadly align with observations by Mensonas et al. [33], who reported that Lithuanian barley accessions from contrasting climatic regions showed distinct physiological and transcriptional adjustments under drought. Shared responses with different magnitudes may reflect quantitative variation in stress signalling, while oppositely regulated or directionally divergent genes suggest that genotype-dependent differences may extend beyond statistically significant DEGs. Mensonas et al. [33] similarly noted subtle but consistent transcriptional shifts among barley accessions, suggesting that small coordinated changes across multiple genes can contribute to divergent drought behaviours. Overall, the interaction patterns observed here may imply that the tolerant genotype engages more coordinated transcriptional adjustments, whereas the sensitive cultivar activates regulatory modules less consistently. This interpretation aligns with the physiological and transcriptomic evidence reported by Mensonas et al. [33], supporting the idea that locally adapted barley genotypes may exhibit more flexible or responsive regulatory behavior under drought.

### 3.4. Signaling Elements

Plant cell membranes function as highly dynamic regulatory platforms that preserve cellular integrity, coordinate transmembrane transport, and integrate environmental and biotic cues into appropriate physiological responses [34]. Complex networks of proteins mediate signal transduction in plant cells, including sensors, transducers, and effectors. These networks encompass receptors, adaptor proteins, kinases, transcription factors, and secondary messengers such as calcium ions (Ca^2+^), hydrogen peroxide (H_2_O_2_), nitric oxide (NO), and abscisic acid (ABA) [35]. Under drought stress, the HvCaSR transcript displayed clear genotype-dependent regulation, with minimal induction in the drought-sensitive cultivar and markedly enhanced expression in the drought-tolerant genotype. The plasma membrane-localized G protein-coupled receptor (GPCR), known as the calcium-sensing receptor (CaSR), plays a central role in maintaining intracellular Ca^2+^ homeostasis and also acts as an extracellular signaling mediator. CaSR activation is not restricted to Ca^2+^; it can also be triggered by other divalent and trivalent cations, polyamines, amino acids, and various polycationic ligands [36]. Through coupling with heterotrimeric G proteins, CaSR initiates multiple intracellular signaling cascades that regulate apoptosis, proliferation, differentiation, chemotaxis, secretion, and ion channel activity, underscoring its pivotal role in plant stress adaptation and developmental regulation [34,36]. Drought stress activates complex and multilayered signaling networks in barley, involving calcium-binding proteins, annexins, protein kinases, and receptor-like kinases. Members of the calmodulin (CaM) and calmodulin-like (CML) families act as key Ca^2+^ sensors and relays [37]. Several HvCML and HvCaM isoforms were strongly up-regulated in tolerant genotypes under drought, whereas the sensitive genotype exhibited weaker induction or even down-regulation. This contrasting transcriptional pattern highlights the critical contribution of Ca^2+^-signaling capacity to drought tolerance [38].

Annexin proteins, particularly *HvAnn1*, were also upregulated under drought stress, with substantially higher expression in the tolerant genotype. Annexins are associated with Ca^2+^ flux regulation and ROS interactions, and their stronger induction in the tolerant genotype may contribute to enhance membrane stability under drought [15,37]. Calcium-dependent protein kinases (CDPKs) and SNF1-related kinases (SnRKs), which belong to the CAMK group, represent additional drought-induced regulators. In tolerant genotypes, these kinases participate in both ABA-independent and ABA-dependent signaling pathways and act through phosphorylation of ion channels and downstream transducers. This dual regulation enables simultaneous control of ion homeostasis and stress-responsive gene expression, thereby reinforcing drought adaptation [18,34]. Receptor-like kinases of the Pelle subfamily were enriched among stress-responsive genes, suggesting a prominent role in early stress perception and initiation of downstream phosphorylation cascades (Table S4) [18]. Importantly, the genotype-dependent differences observed across Ca^2+^ sensors, annexins, CDPKs, SnRKs, and RLKs provide mechanistic insight into genotype—specific differences in drought tolerance. The strong, coordinated induction of these signaling components in the tolerant genotype suggests more effective activation of upstream regulatory networks under drought, including Ca^2^ signaling, ABA-associated pathways, ROS detoxification, and secondary metabolism. In contrast, the attenuated activation of these pathways in the cultivated genotype may limit its ability to initiate efficient downstream responses. Crucially, these genotype-dependent differences are fully consistent with transcriptomic patterns previously reported for Iranian wild barley under heat stress [32] and salinity stress [13]. In both studies, wild germplasm exhibited markedly stronger induction of Ca^2+^-dependent signaling components, ROS-associated regulators, and ABA-linked transcriptional modules compared with cultivated barley. The convergence of these findings across drought, heat, and salinity may indicate that wild barley maintains a conserved regulatory framework that coordinates Ca^2+^ signaling, ABA biosynthesis, ROS detoxification, and secondary metabolism pathways. Taken together, these findings suggest that drought tolerance in barley is associated not only with the coordinated up-regulation of Ca^2+^ sensors (HvCML, HvCaM), annexins (*HvAnn1*), and kinase families (CDPKs, SnRKs, and RLK-Pelle), but also with more effective engagement of upstream regulatory modules in the tolerant genotype. This integrated signaling activity likely contributes to the physiological and biochemical adjustments required for survival under water-deficit conditions.

### 3.5. Phytohormone Biosynthesis

Phytohormones act as central regulatory molecules that coordinate plant growth, development, and stress adaption. In this study, the identification of multiple hormone-related DEGs highlights the critical role of hormonal signaling networks in mediating drought responses. These hormone-associated DEGs illustrated the complexity of hormonal crosstalk and emphasized the importance of integrated phytohormone pathways in shaping stress resilience mechanisms [16,39,40]. The contrasting expression of hormone-related genes between tolerant and sensitive barley genotypes reveals distinct regulatory adjustments underlying drought adaptation. In the tolerant genotype, strong upregulation of the ABA receptor PYL and the transcription factor ABF indicates enhanced activation of ABA signaling, enabling efficient stress perception and downstream transcriptional reprogramming (Figure S6 and Table S5). This pattern is consistent with the established role of PYR/PYL/RCAR receptors and ABFs as core regulators of drought-induced stomatal closure and stress-responsive gene expression [40,41,42]. In contrast, the sensitive cultivar exhibited reduced PYL expression together with elevated PP2C, a negative regulator of ABA signaling. This antagonistic configuration likely weakens ABA-mediated defense processes, despite increased SnRK2 and ABF expression (Table S5), suggesting impaired signaling efficiency and reduced stress tolerance [18]. The PP2C expression pattern reported by Gharaghanipor et al. [13] under salinity stress differs from the drought-induced pattern observed in the present study. In their work, PP2C was up-regulated in the wild genotype, whereas in the present study PP2C showed higher expression in the sensitive cultivar. This difference likely reflects the distinct nature of salinity versus drought stresses, as salinity imposes both osmotic and ionic stress, while drought primarily triggers osmotic stress without ionic toxicity, and the regulatory architecture of ABA signaling responds differently. In the present study, elevated PP2C in the sensitive genotype occurred together with reduced PYL expression, indicating weakened ABA perception rather than adaptive modulation. Meanwhile, the tolerant (wild) genotype showed strong induction of PYL, ABF, and SnRK2, reflecting efficient ABA signal transduction. Therefore, PP2C up-regulation in salinity-tolerant wild barley represents an adaptive regulatory adjustment, whereas PP2C elevation in drought-sensitive cultivated barley reflects impaired ABA signaling. These differences highlight that PP2C behaviour must be interpreted within the broader context of stress type and the overall configuration of ABA signaling networks. Additional hormonal pathways further reinforced these genotype-specific differences. Downregulation of PIF, PIL, and DELLA in the sensitive cultivar indicated disrupted light and gibberellin signaling, which may hinder growth regulation under drought (Table S5). Crosstalk between ABA and gibberellin pathways mediated by DELLA proteins is essential for balancing growth and stress responses, and disruption of this balance may contribute to reduced adaptive capacity in the cultivated genotype [40,43]. Within the auxin pathway, SAUR genes were upregulated in both genotypes, but tolerant plants exhibited substantially higher expression, suggesting stronger auxin responsiveness and better maintenance of growth under water deficit. Conversely, reduced expression of IAA genes in the sensitive genotype indicated auxin pathway dysregulation, consistent with reports that auxin signaling is often suppressed under drought stress to prioritize survival over growth [16]. The upregulation of JAZ genes in the tolerant genotype further suggests a more controlled jasmonate signaling response (Table S5), preventing excessive defense activation that could compromise growth [44]. These hormonal differences are fully consistent with transcriptomic patterns previously reported for Iranian wild barley under heat stress [32] and salinity stress [13]. In both studies, wild germplasm exhibited markedly stronger induction of ABA-linked regulators, Ca^2+^-dependent signaling components, and growth-modulating hormone pathways compared with cultivated barley. Integration of our drought-induced transcriptomic patterns with heat- and salinity profiles suggests that Iranian wild germplasm may maintain a stable regulatory backbone capable of activating upstream hormonal and stress-signaling hubs in a coordinated manner. Importantly, these hormonal differences provide mechanistic insight into genotype—specific regulatory strategies underlying drought tolerance. The coordinated activation of ABA, auxin, gibberellin, and jasmonate pathways in the tolerant (wild) genotype points to a more effectively integrated hormonal regulatory system that supports antioxidant defense and secondary metabolism during drought stress. In contrast, the sensitive (cultivated) genotype exhibited hormonal dysregulation—characterized by weakened ABA perception, disrupted GA signaling, and reduced auxin homeostasis—suggesting less effective engagement of key stress-responsive regulatory modules. The higher ABA accumulation observed physiologically in the tolerant genotype aligns with the transcriptomic activation of ABA-responsive genes, indicating that ABA-dependent signaling represents a major regulatory component of drought tolerance. The coordinated activation of this pathway likely contributes to stomatal closure, osmotic adjustment, antioxidant defense, and transcriptional reprogramming under water-deficit conditions [45,46,47].

### 3.6. Transcription Factors

Plants employ multilayered defense strategies at the molecular, cellular, physiological, and biochemical levels to withstand abiotic stress conditions. Among these, transcription factors (TFs) act as master regulators, orchestrating gene expression networks essential for stress perception and adaptive responses [39]. Major TF families such as AP2/ERF, WRKY, NAC, and MYB are widely recognized for their central roles in modulating drought-responsive pathways [48,49]. In response to drought stress, TFs are rapidly activated to initiate downstream regulatory cascades. In this study, strong induction of AP2/ERF-ERF, bHLH, MYB, Trihelix, WRKY, TCP, Coactivator p15, CDS and E2F-DP TFs occurred exclusively in the drought-tolerant genotype, indicating a more dynamic transcriptional reprogramming capacity. In contrast, TF families such as NAC, HSF, and bZIP exhibited shared activation patterns in both genotypes, reflecting their fundamental roles in core stress responses irrespective of tolerance levels (Table S6). Members of the NAC, WRKY, DREB/CBF, and bZIP families are well-known upstream regulators of abiotic stress responses and may control many of the physiological and biochemical adaptations observed in the tolerant genotype [50,51]. Their enhanced expression in wild barley aligns with previous transcriptomic reports in barley [52] and wheat [48], and with TF expression patterns reported in other crops, including rice [53] and maize [54].

The contrasting TF profiles visualized in the two genotypes further reinforce these regulatory differences. The drought-tolerant genotype showed broad activation of TF families, with AP2/ERF, bHLH, MYB, WRKY, Trihelix, TCP, and E2F-DP showing the highest numbers of DEGs. This wide transcriptional engagement suggests a more coordinated regulatory response, potentially supporting hormonal signaling, antioxidant defense, osmotic adjustment, and secondary metabolism. In contrast, the drought-sensitive genotype exhibited a markedly narrower TF activation profile, with fewer DEGs across most families and weaker induction-even within major TF groups such as AP2/ERF, NAC, bHLH, and bZIP. Such limited transcriptional engagement may indicate reduced regulatory flexibility under drought stress. Integration of drought-responsive TF profiles obtained in the present study with transcriptomic patterns reported for Iranian wild barley under heat stress [32], salinity stress [13], and drought stress [15], further supports the notion that wild germplasm maintains a highly responsive transcriptional framework. In all three studies, wild barley exhibited stronger and broader activation of key TF families—including AP2/ERF, bHLH, MYB, WRKY, NAC, HSF, and bZIP—compared with cultivated genotypes. The convergence of TF-activation patterns across drought, heat, and salinity suggests a robust regulatory architecture in wild barley under diverse environmental stresses. Importantly, the genotype-specific TF activation patterns provide insight into regulatory strategies underlying drought tolerance. The coordinated induction of ERF, MYB, WRKY, and bHLH TFs in the tolerant genotype suggests stronger engagement of regulatory modules associated with ABA signaling, flavonoid biosynthesis, ROS detoxification, and membrane stabilization. These TF families are known to activate ABA-responsive genes, phenylpropanoid pathway enzymes, and antioxidant defenses mechanisms that collectively support stress adaptation. In contrast, the cultivated genotype exhibited weaker or fragmented activation of key TF families, suggesting reduced engagement of upstream regulatory hubs. This diminished regulatory input likely restricts induction of ERF, MYB, and WRKY TFs, limiting downstream activation of ABA-dependent pathways and secondary metabolism and ultimately contributing to the suppressed hormonal and flavonoid responses observed in the sensitive cultivar. Taken together, these findings demonstrate that drought tolerance in wild barley is associated with stronger transcriptional activation of regulatory networks that integrate hormonal signaling, antioxidant defense, and secondary metabolism. In contrast, the sensitive genotype, shaped by domestication bottlenecks, exhibits reduced TF activation and lower regulatory coherence, ultimately leading to weaker stress adaptation.

### 3.7. Ion Transporters and Channels

Drought stress induces biophysical modifications in membrane fluidity that serve as early cues for environmental perception and gene regulation [37]. Membrane-associated proteins sense these alterations, detect phase transitions, and initiate phosphorylation cascades that ultimately shape stress-responsive gene expression [15]. In the present study, drought-tolerant barley genotypes exhibited similar shifts in membrane fluidity accompanied by the activation of phosphorylation-related signaling pathways, supporting a role for membrane dynamics in early stress perception. Wild barley (*H. spontaneum*) genotypes are known to upregulate ion channels and transporters under drought conditions, facilitating precise regulation of osmotic balance, hormonal signaling, and water retention [15,37,55]. Consistent with these observations, our study revealed increased expression of ion-transport-related genes in drought-tolerant genotypes, indicating enhanced capacity for ionic adjustment and hormonal regulation (Table S7). Among these transporters, ABC transporters play important roles in water regulation, hormone transport, and ROS detoxification, with ABCG subfamilies members frequently represented among drought-responsive genes [56]. The upregulation of ABCG genes in the tolerant genotype suggests their involvement in antioxidant transport and stress mitigation.

MFS transporters complement these mechanisms by using proton gradients to move ions (Na^+^, K^+^, H^+^), sugars, and metabolites, thereby maintaining osmotic pressure and supporting cellular energy balance under drought stress [57]. Both ABC and MFS transporters are central to ROS detoxification and the transport of key antioxidants, such as glutathione. In our study, MFS transporter genes showed strong induction, particularly those associated with sugar and ion transport, suggesting their potential roles in osmotic adjustment and metabolic stability (Table S7).

Sulfate transporters enhance glutathione biosynthesis and interact with proline and hormone pathways to alleviate oxidative damage [57]. Drought-tolerant genotypes exhibited higher expression of sulfate transporter genes, which may contribute to increased GSH accumulation and reduced oxidative stress. Potassium transporters regulate turgor pressure, stomatal conductance, and defense signaling, while Na^+^/Ca^2+^ exchangers maintain ionic homeostasis and modulate calcium signaling during drought [58,59]. Consistent with these functions our data showed significant upregulation of potassium and Na^+^/Ca^2+^ transporter genes, suggesting improved stomatal control and ionic balance in the tolerant genotype.

Plasma membrane H^+^-ATPases generate electrochemical gradients essential for ion uptake and water absorption. Their elevated expression in drought-tolerant genotypes has been associated with improved photosynthesis and stress signaling, particularly through ABA-mediated regulation of stomatal closure [60]. Consistent with these observations, our findings showed strong induction of H^+^-ATPase genes under drought stress, paralleling increases in photosynthetic activity and ABA-related responses.

Additional transporters, including arsenical pump ATPases, auxin efflux carriers, and members of the NRT1/PTR family, contribute to ion detoxification, hormone distribution, and nitrate/peptide signaling pathways. Notably, NRT1/PTR family 7.1 was upregulated in drought-tolerant genotypes (Table S6), which may support root growth and grain yield [61,62]. In line with previous results, our transcriptomic data revealed a substantial induction of auxin transporter genes under drought stress, reinforcing their potential involvement in adaptive growth and stress-response mechanisms.

The ion-transport and membrane-associated signaling patterns identified in the present study are consistent with previous transcriptomic evidence reported for Iranian wild barley under heat [32], salinity [13], and drought stress [15]. Across all three studies, wild genotypes exhibited markedly stronger induction of key ion transport systems—including K^+^ channels, Na^+^/Ca^2+^ exchangers, H^++^ATPases, ABC and MFS transporters—compared with cultivated barley. This convergence suggests a more responsive membrane transport architecture in wild barley. The enhanced activation of these transport modules in wild germplasm aligns with the increased accumulation of essential nutrients observed in the tolerant genotype in the present study, highlighting ion transport as a key component of drought-resilience strategies. Together, these transporter systems form a coordinated regulatory network that appears more robust in drought-tolerant genotypes. The extensive induction of transporter-related genes suggests integrated activation of membrane signaling, ion homeostasis, and antioxidant transport, enabling rapid physiological adjustment under water-deficit conditions in the tolerant genotype. In contrast, the drought-sensitive genotype exhibited weaker or more fragmented activation of these transport pathways, suggesting reduced regulatory flexibility and diminished capacity to stabilize cellular homeostasis during stress. The physiological relevance of these transcriptional responses is further supported by the ICP-MS analysis, which revealed enhanced accumulation of K, P, Fe, Zn, Mn and S in the drought-tolerant genotype. This coordinated increase in nutrient retention, together with strong transporter gene induction, suggests that maintaining ion homeostasis is a central component of the drought-resilience in wild barley.

### 3.8. Genes Encoding ROS Scavenging Proteins

Environmental stresses such as drought trigger electrolyte leakage, excessive ROS production, and free radical accumulation, thereby disrupting cellular homeostasis. These processes promote lipid peroxidation, membrane destabilization, and enzyme impairment, ultimately threatening cell viability [16,32]. In the present study, drought stress markedly increased ROS and free radical production; however, the drought-tolerant barley genotype strongly suppressed ROS accumulation, likely through coordinated activation of antioxidant pathways. To mitigate ROS toxicity, plants rely on an integrated antioxidant machinery comprising enzymatic components—such as glutathione S-transferases (GSTs), peroxidases, and ferredoxins—as well as diverse secondary metabolites. Wild barley genotypes exhibited strong induction of antioxidant-related genes, with GST showing the highest representation, suggesting an important role for GSTs in oxidative stress mitigation (Table S8) [32,63]. Previous studies have similarly highlighted GST as a key antioxidant enzyme. For example, Ashoub et al. [64] reported increased expression of *GST*, peroxidase, and thioredoxin genes in drought- and heat-tolerant *H. spontaneum* genotypes. Other studies have also shown higher expression of *SOD*, *APX*, and *GST* in drought-tolerant barley than in sensitive cultivars, supporting the link between antioxidant gene activation and stress resilience [15,63].

Consistent with earlier findings, our results show that drought substantially elevates ROS accumulation; however, tolerant barley genotypes counteract this via robust ROS-scavenging pathways. Notably, genes encoding peroxidase (*HvPOD*) and ferredoxin (*HvFdx3*) were strongly upregulated in wild barley. The drought-tolerant *H. spontaneum* genotype displayed significant increases in peroxidase gene expression, indicating that enhanced peroxidase activity may contribute to drought tolerance (Table S7). These results align with the reports by Karamzadeh et al. [16] on drought stress, Bahrami et al. [32] on heat stress, and Gharaghanipor et al. [13] on salinity stress, collectively emphasizing the cross-stress importance of peroxidase enzymes.

Beyond enzymatic antioxidants, flavonoid biosynthesis represents an additional protective mechanism under drought. In our study, genes associated with flavonoid biosynthesis were strongly upregulated in the tolerant genotype but downregulated in the sensitive one, indicating genotype-specific differences in secondary metabolism. Key enzymes, including Phenylalanine Ammonia Lyase (PAL), chalcone synthase (CHS) and chalcone-flavanone isomerase (CHI), showed marked upregulation in the tolerant genotype, suggesting their contribution to ROS scavenging and adaptive responses. These genes represent promising molecular targets for enhancing drought tolerance in barley through future breeding or biotechnological approaches.

Another key enzyme, Fe_2_OG dioxygenase domain-containing protein, was notably downregulated in the sensitive cultivar ‘Mona’ (Figure S7b and Table S8). These enzymes catalyze hydroxylation steps within the flavonoid biosynthesis pathway, converting flavanones into more reactive antioxidant molecules. Their expression is tightly regulated under drought stress, and tolerant genotypes consistently show elevated transcript levels—particularly in *PAL* and *CHS*—highlighting their central role in adaptive responses. Previous studies, [65], have demonstrated the involvement of 2-oxoglutarate/Fe(II)-dependent dioxygenases in stress tolerance. Moreover, Wang et al. [66] have confirmed the positive regulatory role of these enzymes in abiotic stress resilience through modulation of flavonoid accumulation. Likewise, Shirvani et al. [15] also reported increased expression of *PAL*, *CHS*, and *CHI* in drought-tolerant barley genotypes, and Gharaghanipor et al. [13] showed similar upregulation of flavonoid pathway genes in wild *H. spontaneum* under salinity stress, further emphasizing the pathway’s importance in stress tolerance. Transcriptomic studies confirm that glycosyltransferase genes are upregulated in drought-tolerant barley and contribute an additional regulatory layer by stabilizing flavonoids through glycosylation of compounds such as quercetin and naringenin, thereby reducing ROS-induced degradation. Glycosylation also detoxifies potentially harmful free phenolics and increases solubility, thereby contributing to osmotic balance. These glycosylated forms can be stored in vacuoles and activated when needed under stress conditions. Transcriptomic evidence indicated that glycosyltransferase genes—particularly members of the UDP-glycosyltransferase (UGT) family—are strongly upregulated in drought-tolerant barley genotypes, leading to greater accumulation of glycosylated flavonoids and reduced oxidative damage [21]. Our results align with these findings, showing robust induction of UGT genes in the tolerant genotype, underscoring their importance in ROS detoxification and drought adaptation. These results are further supported by Feng et al. [67], who identified *HvUGT1* as a drought-responsive UGT gene contributing to enhanced antioxidant capacity in wild barley, as well as by Shirvani et al. [15] and He et al. [68], who confirmed the pivotal role of UGT-related genes in drought tolerance through comparative transcriptomic analyses.

Collectively, the transcriptional activation of genes involved in ROS scavenging, redox homeostasis, flavonoid biosynthesis, and antioxidant metabolism suggests that the tolerant genotype possesses a more effective oxidative stress management system. These transcriptomic responses were consistent with physiological evidence, which showed lower H_2_O_2_ and MDA accumulation and enhanced antioxidant activity in the drought-tolerant genotype. The convergence of physiological and transcriptomic evidence highlights oxidative stress mitigation as a component of drought adaptation in *H. spontaneum*. A cross-study comparison further demonstrates that Iranian wild barley has exhibited a consistent oxidative-stress regulatory framework across multiple abiotic stresses. Transcriptomic analyses under heat [32], salinity [13], and drought [15] consistently show that wild genotypes activate broader and more integrated redox regulatory networks than cultivated barley. While these patterns suggest an evolutionarily preserved feature of *H. spontaneum*, additional comparative studies could help confirm the extent of this conservation. Overall, oxidative stress mitigation appears to form a foundational component of its superior stress-adaptive capacity in wild barley.

### 3.9. Genes Involved in Osmotic Homeostasis and Accumulation of Compatible Solutes

Previous studies have demonstrated that LEA (Late Embryogenesis Abundant) genes play a pivotal role in plant responses to a range of abiotic stresses, including drought [69,70,71], high temperature [32], and salinity [13]. LEA proteins, characterized by their high hydrophilicity and exceptional stability under stress conditions, function as membrane stabilizers and protect essential cellular components from dehydration-induced damage [69,70,71]. According to Qin et al. [12] and Mohanty et al. [71], the greater accumulation of LEA proteins in drought-tolerant genotypes represents a major protective mechanism, enabling plants to withstand water-deficit conditions. Their role in barley drought tolerance has also been confirmed by Zaidi et al. [72].

In this study, the elevated abundance of LEA proteins in the wild drought-tolerant genotype suggests their contribution to early protective response under drought (Table S9). Dehydrins, a well-known subclass of the LEA protein family, contribute to drought resilience by stabilizing cellular membranes, safeguarding nucleic acids, and preventing denaturation of sensitive proteins. The strong induction of LEA and dehydrin genes in the tolerant genotype suggests enhanced cellular protection. LEA proteins also help scavenge reactive oxygen species (ROS), enhancing oxidative stress resistance [73]. Their elevated expression in the tolerant genotype is consistent with improved stress tolerance.

The present study also underscores the role of heat shock proteins (HSPs) in mediating drought stress tolerance in barley. The pronounced up-regulation of HSP20, HSP70, HSP90, HSP110, and small HSPs (sHSPs) in both wild barley and the Mona cultivar suggests that these molecular chaperones support membrane stability and protein homeostasis under drought (Table S9). These observations align with previous reports indicating that HSPs maintain protein structure, prevent aggregation, and support refolding of denatured proteins during abiotic stress [15,74].

The functional significance of HSP20 genes in plant growth, development, and drought tolerance has been well documented. Genome-wide analyses in barley [75], wheat [76], and maize [77], consistently demonstrated strong induction of HSP20 members under drought stress. Beyond stress associated roles, HSP20 proteins also participate in developmental processes, acting as protective chaperones under both optimal and adverse conditions [78]. The coordinated induction of multiple HSP families observed here suggests their integration into broader regulatory networks that support both stress adaptation and developmental stability.

Another notable finding is that the genes HvP5Cr3 and HvP5CSB, which encode key enzymes in proline biosynthesis, were commonly expressed in both subspecies (Table S9). This conserved regulatory pattern underscores proline’s central role in osmotic adjustment, membrane stabilisation, and ROS detoxification under drought stress. Numerous studies have reported that the up-regulation of proline biosynthesis genes (*P5CS* family) is a common response to drought stress across different plant species. For instance, Shrestha et al. [79] demonstrated that natural allelic variation in *HvP5CS1* contributes significantly to drought adaptation and yield stability in barley. Likewise, Frimpong et al. [80] showed that proline-mediated drought tolerance in barley is associated with enhanced lateral root development during early growth stages. In *Arabidopsis*, Aleksza et al. [81] reported strong induction of *P5CS1* under drought and phosphate starvation, leading to increased proline accumulation; while Yan et al. [82] highlighted proline as a multifunctional amino acid central to osmotic regulation and oxidative stress mitigation during water deficit. The shared induction of proline biosynthesis genes in both genotypes suggests that proline accumulation represents a conserved mechanism of drought tolerance in barley.

Aquaporins, membrane channel proteins that facilitate water and small-solute transport, exhibited genotype-specific expression under drought stress. In the sensitive cultivar, *HvPIP2;5* was downregulated, likely to reduce water loss, whereas *HvTIP1;1* was upregulated to enhance intracellular water retention. In contrast, aquaporin gene expression remained relatively stable, in the tolerant genotype, suggesting more balanced water regulation (Table S9). Severe downregulation of TIPs in sensitive barley has been associated with reduced turgor pressure, leaf wilting, and oxidative damage [83,84]. Numerous studies have reported that aquaporin genes (PIPs and TIPs) are frequently upregulated under drought stress to maintain water homeostasis. For example, Alam et al. [84] demonstrated induction of PIP aquaporins in *Arabidopsis* under water deficit, improving hydraulic conductivity. Ost et al. [85] showed that aquaporin regulation in barley roots is strongly linked to ABA signaling, while Afzal et al. [86] reported that PIP2 isoforms in cereals facilitate efficient water transport under drought. Similar induction patterns have been observed in rice [87]. The stable expression patterns in the tolerant genotypes may contribute to improved hydraulic balance.

In addition to aquaporin regulation, detoxification-related genes such as Peptidase S59 and Agmatine Coumaroyl Transferase-2 were exclusively upregulated in the tolerant genotype (Table S9). Peptidase S59, a serine protease domain, participates in selective protein degradation, helping remove damaged proteins and maintain metabolic balance under stress [88]. Agmatine Coumaroyl Transferase-2 contributes phenolic and alkaloid biosynthesis, reinforcing cell wall integrity under drought stress [89,90]. Their exclusive induction in the tolerant genotype suggests that enhanced detoxification capacity is an important component of its drought-resilience strategy. Cross-study comparisons further highlight that Iranian wild barley exhibits a consistent regulatory architecture across heat [32], salinity [13], and drought [15]. Wild barley consistently upregulates protein-stabilising modules, osmotic regulatory pathways, and membrane-protective systems, enabling sustained cellular function under water deficit.

Together, our results indicate that drought tolerance in wild barley does not rely on a single protective mechanism but emerges from coordinated interactions among hormonal regulation, antioxidant defence, nutrient homeostasis, osmotic adjustment, and transcriptional reprogramming. Although these responses function as an interconnected network, stepwise examination clarifies their mechanistic roles. In the tolerant (wild) genotype, physiological, ionomic and transcriptomic data collectively reveal that drought-induced Ca^2+^ influx—reflected by altered expression of calcium sensors—may act as an upstream trigger for MAPK activation. Increased expression of specific MAPKs suggests engagement of these cascades, which subsequently modulate key transcription factor families. Consistent with this, the tolerant genotype showed significant upregulation of bZIP, DREB/CBF, bHLH and NAC transcription factors, indicating potential MAPK-linked activation under drought. These TFs then drive the expression of downstream protective genes. Elevated LEA and dehydrin expression reflect activation of protein-stabilizing pathways, while marked induction of P5CS indicates enhanced proline biosynthesis and osmotic adjustment. Increased expression of aquaporins (PIP/TIP) and ion-transport regulators demonstrates integration of water balance and ionic homeostasis. Furthermore, upregulation of antioxidant genes suggests that TF activation extends to ROS-detoxification pathways. Together, these coordinated upstream–downstream interactions—Ca^2+^ perception, MAPK activation, TF regulation, and induction of LEA, dehydrin, P5CS, aquaporins, ion-transport genes, and ROS-detoxifying enzymes—suggest that drought tolerance in wild barley arises from a multilayered, synergistic regulatory network. This integrated architecture enables wild barley to maintain cellular function, metabolic stability, and growth under water-limited conditions and provides a mechanistic framework for understanding drought adaptation in *H. spontaneum*, highlighting valuable targets for future barley improvement programs.

#### Future Perspectives

Building on these findings, future research could substantially deepen mechanistic understanding of drought tolerance in barley and enhance the translational value of these results. Although our integrated physiological, elemental and transcriptomic analyses identified key regulatory nodes within the drought-responsive network, determining the causal contribution of these candidate genes requires functional validation through approaches such as transient transformation or CRISPR/Cas-based genome editing. Establishing which genes play direct, essential roles in drought tolerance would clarify the hierarchy of regulatory interactions, distinguish primary drivers from secondary stress-associated responses, and refine the conceptual framework of drought adaptation in barley. Future transcriptomic studies with expanded experimental designs could incorporate genotype × treatment interaction models to more precisely dissect genotype-specific regulatory differences under drought stress. Such mechanistic resolution is critical for developing reliable molecular markers and selecting robust breeding targets. Functionally validated genes represent powerful tools for crop improvement; Introducing these genes into high-potential commercial cultivars—whether through marker-assisted selection, genomic selection or targeted gene transfer—could enhance yield stability under water-limited conditions and strengthen the resilience of barley production systems in drought-prone environments. This strategy not only improves performance under stress but also supports long-term agricultural sustainability by reducing vulnerability to climate-induced water scarcity. Expanding future analyses to additional developmental stages, varying drought intensities, and root or grain tissues will further clarify how these regulatory pathways operate across the whole plant and help translate molecular insights into practical breeding programs to develop drought-resilient barley cultivars.

## 4. Materials and Methods

### 4.1. Plant Materials

This study used two barley genotypes with contrasting drought tolerance. The wild genotype *H. spontaneum* no. 11BP395 was selected from germplasm collected in western Iran. This accession originated from the Urmia Lake region (37°11′34.0″ N, 45°21′43.4″ E; 1292 m). ‘Mona’ an early-maturing barley cultivar of Swedish origin, was used as the drought-sensitive genotype. Individual plants were taken from each collection site. The collected seeds from each plant (pure line; hereafter termed “genotype”) were subsequently planted in separate rows to increase seed production under field conditions. Our previous field study [16] showed that the wild genotype 11BP395 had strong drought tolerance, including lower yield reduction, enhanced antioxidant activity, increased phenolic accumulation, and favourable ABA regulation. In contrast, ‘Mona’ experienced greater yield loss, higher MDA and H_2_O_2_ accumulation, and lower antioxidant activity, phenolic content, and drought-related hormonal regulation under water-deficit conditions. These contrasting genotypes also exhibited significant differences in grain yield and drought-induced yield reduction, providing the basis for their selection in the present mechanistic study.

### 4.2. Experimental Design and Drought Stress Assay

The seedling stage was selected for this study because it provides a controlled system for linking physiological responses with transcriptomic changes during early drought stress. The specific RNA-seq sampling time point within this stage was determined based on preliminary morphological, physiological, and biochemical assessments that revealed clear genotype-specific differences under drought stress. Drought progression was monitored through visible leaf wilting, reductions in relative water content (RWC) and chlorophyll levels, and increases in oxidative stress markers (MDA and H_2_O_2_). The chosen time point corresponded to the stage at which these indicators showed the strongest divergence between the drought-tolerant wild genotype and the sensitive cultivar, ensuring that the transcriptomic profiles captured biologically meaningful drought-response signatures. Seed germination of the genotypes was performed on moist filter paper at 4 °C. The seedlings were moved to plastic pots (30 cm in diameter) filled with mixed clay and sand (2:1) in greenhouse conditions at a photoperiod of 12 h daytime/12 h nighttime with a photosynthetic photon flux density (PPFD) of ∼350 µmolm^−2^ s^−1^, day–night temperatures 25 ± 3–17 ± 3 °C, and relative humidity ∼50%.

Plants under both experimental conditions (control and drought stress) were initially irrigated at sowing, then irrigated every 3 days until they reached the two-leaf stage. Half of the pots were watered normally (control), and the other half were subjected to drought stress for 3 weeks. Upon initiating the stress treatments, soil moisture in the root zone (0–20 cm depth) was measured and the irrigation volume required to replenish the deficit up to field capacity was determined. The irrigation depth (Id), expressed in centimeters, was calculated using the formula:

I_d_ = (θFC − θirri) × D × pb/pw

In this equation, θFC represents the soil gravimetric moisture percentage at field capacity, θirri is the soil gravimetric moisture percentage at the time of irrigation, D denotes the root zone depth, pb is the soil bulk density at the root zone (1.4 g cm^−3^), and pw is the water density.

To track the volume of each water application, a Parshall flume was utilized (where Id = I × p, with p representing the fraction of I that could be depleted from the root zone). Additionally, the gross irrigation requirement (Ig) was calculated using the equation Ig = (Id/Ea) × 100, where Ea denotes irrigation efficiency (%), and is assumed to average 85%. For the RNA-seq analysis, the fresh leaves were collected from 10 plants in each entry, immediately frozen in liquid nitrogen, and kept at −80 °C until the extraction of RNA. The RNA-seq study used two biological replicates per treatment (control and drought stress) and per genotype (tolerant and sensitive).

### 4.3. Morpho-Physiological Study

Plant height was measured at the grain-filling stage from the soil surface to the tip of the main spike. A comprehensive set of physiological traits was also measured using four biological replicates per treatment during the seedling stage. Concise methodological principles are provided in the main text, while additional details are presented in Supplementary Methods. Analysis of variance of the data was performed using the GLM procedure of SAS software (SAS 9.4, SAS Institute, Cary, NC, USA). Furthermore, mean comparisons were conducted using Fisher’s least significant difference (LSD) at a significance level of *p* ≤ 0.05.

#### 4.3.1. Membrane Stability Index (MSI) & Relative Water Content (RWC)

Leaf membrane stability was assessed through electrolyte leakage following Sairam [91] Leaf tissues (0.2 g) were incubated in double-distilled water at 40 °C, and the initial conductivity (C1) was recorded. Samples were then boiled at 100 °C to obtain the final conductivity (C2). The membrane stability index (MSI) was calculated as MSI (%) = [1 − (C1/C2)] × 100. Relative water content (RWC) was determined according to Barrs and Weatherley [92]. Fresh leaf discs (0.2 g) were weighed (FW), fully hydrated to obtain turgid weight (TW), and then oven-dried to determine dry weight (DW). Relative water content was calculated as RWC (%) = [(FW − DW)/(TW − DW)] × 100.

#### 4.3.2. Chlorophylls (Chl) and Carotenoids (Car)

Chlorophylls and carotenoids were quantified following the standard acetone extraction method of Lichtenthaler [93]. Fresh leaf samples were extracted in 80% acetone, and absorbance was measured at 663, 646, and 470 nm using a UV–visible spectrophotometer. Concentrations of chlorophyll a, chlorophyll b, and total carotenoids were calculated using Lichtenthaler’s equations and expressed on a fresh mass basis (mg/g fresh weight).

#### 4.3.3. Proline Content

Proline content was quantified using the ninhydrin-based colorimetric method of Bates et al. [94]. Fresh leaf tissues were extracted in 3% sulfosalicylic acid, and the resulting supernatant was reacted with acid ninhydrin and glacial acetic acid. After incubation in a boiling water bath, the chromophore was extracted with toluene, and its absorbance was measured at 520 nm. Proline concentration was determined from a standard curve and expressed on a fresh mass basis.

#### 4.3.4. Abscisic Acid Extraction and Quantification

Abscisic acid (ABA) was extracted using 80% methanol containing PVP, followed by centrifugation, pH adjustment, solvent evaporation, and ethyl acetate purification [95]. The final extract was filtered and analyzed by HPLC on a C18 column using a methanol–acetic acid gradient; ABA quantification was performed against authenticated standards and expressed on a fresh weight basis. Indole-3-acetic acid (IAA) and gibberellins (GA) were extracted in cold 80% methanol, purified through sequential solvent washes and C18 solid-phase extraction, and finally analyzed by HPLC equipped with a photodiode array detector [96]. Chromatographic separation was performed on a reversed-phase C18 column using methanol–formic acid gradients, and hormone concentrations were determined using standard calibration curves and reported on a fresh weight basis [97,98].

#### 4.3.5. Antioxidant Defense Parameters

Enzymatic antioxidant activities (POX, APX, and CAT) were determined using crude protein extracts prepared from fresh leaf tissues. Total soluble proteins were extracted in phosphate buffer and quantified using the Bradford [99] assay, and enzyme activities were normalized to protein content (U mg^−1^ protein) [100,101]. All enzymatic measurements were performed on fresh leaf tissues, and activities were expressed on a fresh weight basis. For POX, the increase in absorbance at 470 nm due to tetraguaiacol formation was measured following Hemeda and Klein [102]. APX activity was determined by monitoring the decrease in absorbance at 290 nm corresponding to ascorbate oxidation, following Nakano and Asada [103]. CAT activity was measured according to Aebi [104] by recording the decline in absorbance at 240 nm as hydrogen peroxide was decomposed.

#### 4.3.6. Non-Enzymatic Antioxidants

Total phenolic content (TPC) was measured using the Folin–Ciocalteu colorimetric method [105], where methanolic extracts of leaf tissue were reacted with Folin reagent and sodium carbonate, and absorbance was recorded at 765 nm. TPC was quantified using tannic acid as the calibration standard and expressed on a dry weight basis. Total flavonoid content (TFC) was determined using the aluminium chloride method, in which extracts were sequentially reacted with NaNO_2_, AlCl_3_, and NaOH, and absorbance was measured at 510 nm. TFC was expressed as quercetin equivalents on a dry weight basis. Antioxidant capacity was assessed using the DPPH radical scavenging assay as described by Brand-Williams et al. [106]. Plant extracts were mixed with DPPH solution, incubated, and absorbance was measured at 517 nm. Radical scavenging percentage was calculated relative to the control, with BHT used as the positive standard, and results were expressed on a dry weight basis.

#### 4.3.7. Oxidative Stress Indicators

Lipid peroxidation products (TBARS), expressed as MDA equivalents, were quantified using the thiobarbituric acid (TBA) assay following Taulavuori et al. [107]. Because the TBA assay detects a mixture of aldehydic lipid peroxidation products rather than MDA specifically, the values were reported as MDA equivalents in accordance with Johnston et al. [108]. Leaf extracts were reacted with TBA, heated, cooled, and absorbance was measured at 532 and 600 nm to correct for nonspecific turbidity. MDA concentration was expressed as nmol g^−1^ fresh tissue. Hydrogen peroxide (H_2_O_2_) content was determined according to Velikova et al. [109]. Leaf extracts prepared in TCA were reacted with phosphate buffer and potassium iodide, incubated in the dark, and absorbance was recorded at 410 nm. H_2_O_2_ concentration was expressed as nmol g^−1^ fresh tissue.

#### 4.3.8. Element Measurement Using ICP-MS

The elemental concentrations in the plant samples were measured using Inductively Coupled Plasma Mass Spectrometry (ICP-MS). Leaf ionomic analysis was performed on samples collected at the seedling stage, whereas grain elemental concentrations were determined using mature grains obtained at the grain filling stage. For this purpose, dried and ground plant tissues were digested in a closed-vessel microwave digestion system using a mixture of concentrated nitric acid (HNO_3_) and hydrogen peroxide (H_2_O_2_). Following digestion, the solutions were filtered and diluted to a specific volume with deionized water. The final extracts were analysed using Inductively Coupled Plasma Mass Spectrometry (ICP-MS) following the standard operating procedures of ZarAzma Analytical Laboratory (Tehran, Iran) [110] according to the manufacturer’s instructions. Calibration curves were prepared using multi-element standard solutions, and quality control was done through blank samples, certified reference materials (CRMs), and replicated measurements. Finally, elemental concentrations were expressed on a dry weight basis.

### 4.4. RNA Sequencing

Total RNA was extracted from leaf samples collected at the seedling stage. Sampling was performed on fully expanded young leaves, which were immediately frozen in liquid nitrogen after collection to preserve RNA integrity. RNA extraction was carried out using the RNA Isolation Kit (Roche, Diagnostics, Mannheim, Germany) following the manufacturer’s protocol. The concentration and purity of the extracted RNA were assessed using a NanoDrop 8000 spectrophotometer (Thermo Fisher Scientific, Waltham, MA, USA). To eliminate residual genomic DNA, the RNA samples were treated with DNase I. The purified RNA samples were then sent to Novogene Co., Ltd. (Cambridge, UK) for transcriptome sequencing. RNA-Seq libraries were prepared using the TruSeq Stranded mRNA LT Sample Prep Kit (Illumina, San Diego, CA, USA) according to the manufacturer’s instructions (Illumina Part #15031047 Rev. E). Sequencing was performed on the Illumina NovoSeq 6000 platform, generating 151 bp paired-end reads. Trimmomatic v0.39 [111] was used to remove adapter sequences, reads containing undetermined nucleotides, and low-quality bases. The quality of the cleaned sequencing data was evaluated using FastQC v0.12.0 before proceeding with downstream bioinformatics analyses. FastQC provided metrics including total read count, read length distribution, K-mer content, presence of ambiguous bases, and levels of read duplication.

### 4.5. Differential Expression Genes

Four RNA-Seq sample groups (tolerant control, tolerant stress, sensitive control, and sensitive stress), each with two biological replicates, were used for the analyses described below. RNA sequencing generated an average of 7.1 Gb of data per sample and approximately 55 million paired-end reads. In total, 453 million clean reads, ranging from 40 to 71 million read pairs per sample, across the eight datasets. FastQC was used to assess read count, read length, K-mer content, ambiguous bases, and duplication levels. Raw reads were first processed with Trimmomatic v0.39 [111] to remove adapter sequences, filter out low-quality reads (Phred score ≥ 30), and discard reads shorter than 50 bp. Clean reads from each sample were then aligned to the barley cultivar ‘Morex’ reference genome (Ensembl database; Hordeum_vulgare.IBSC_v3.dna.toplevel.fa) using STAR [112]. Gene and isoform expression levels were quantified using RSEM v1.3.2 [113]. Expression values were subsequently estimated with Cufflinks v2.2.1 based on fragments per kilobase of transcript per million mapped reads (FPKM). Statistical analysis of the transcriptome data was performed using the DESeq2 v1.52.0 (Bioconductor 3.23) package in R v4.6.1 [114]. Differentially expressed genes were identified using an FDR-adjusted *p*-value ≤ 0.05 and an absolute log_2_ fold change (|log_2_FC|) ≥ 2 for downstream analyses.

### 4.6. Genotype × Treatment Interaction Analysis

Differential response between genotypes was tested using an integrated DESeq2 model (~genotype + treatment + genotype:treatment) fitted to all eight samples, with cv. Mona and Control were set as reference levels. The Wald test was applied to the interaction coefficient (FDR ≤ 0.05, Benjamini–Hochberg). Genotype-specific drought effects were obtained from the same model by linear contrasts (Mona: treatment coefficient; wild: treatment + interaction coefficients). Sensitivity was assessed with a likelihood-ratio test against the reduced model (~ genotype + treatment) and a Wald test with |Δlog2FC| ≥ 1. Genes with ≥10 counts in ≥2 samples were retained before analysis.

### 4.7. Functional Annotation, Gene Ontology, and Pathway Enrichment Analyses

To enhance functional annotation of the differentially expressed genes (DEGs), the Ensembl Plants database (https://plants.ensembl.org/index.html; accessed on 21 April 2025) was used. Gene Ontology (GO) enrichment analysis was performed for the four DEG groups using the g: Profiler platform (https://biit.cs.ut.ee/gprofiler/; accessed on 6 July 2025), applying the Benjamini–Hochberg FDR correction with a significance threshold of *p* ≤ 0.05 to identify enriched biological processes, molecular functions, and cellular components [115]. Annotated genes were further mapped to metabolic and signaling pathways through the Kyoto Encyclopedia of Genes and Genomes (KEGG) using the KEGG Automatic Annotation Server (KAAS; https://www.genome.jp/kegg/kaas; accessed on 2 September 2025).

### 4.8. Expression Validation Using Quantitative Real-Time PCR

To validate the RNA-Seq results, the relative expression levels of five candidate genes [*Dhn1*, *HvbZIPx*, *HvBip-70*, *HvPht1-6* and *HvCBF6*], which were up-regulated in the drought-tolerant wild genotype under drought stress, were quantified. Gene-specific primer pairs were designed using Primer3 software (http://primer3.ut.ee) and are listed in Table 3. Total RNA was extracted from leaf tissues of wild and cultivated barley grown under control and drought-stress conditions, using the RNA Isolation Kit (Qiagen, Hilden, Germany) according to the manufacturer’s protocol. For each sample, cDNA synthesis was performed using the RevertAid™ First Strand cDNA Synthesis Kit (Thermo Fisher Scientific, cat. no. K1612) according to the manufacturer’s instructions. RT-qPCR analysis was conducted using the RealQ Plus 2× Master Mix Green (Ampliqon, Odense, Denmark) to measure the expression of the five target genes identified as showing differential expression in the transcriptomic DEG analysis. Table 3 presents the primer pairs used for amplification. The β-tubulin gene served as the internal reference for normalization of expression levels. Relative gene expression was calculated using the 2^−ΔΔCT^ method [116]. Statistical differences in transcript abundance between wild and cultivated barley genotypes, relative to their respective controls, were evaluated using the Mann–Whitney U test.

## 5. Conclusions

In our study, growth, physiological, and transcriptomic data consistently indicated drought resilience in wild barley, which reflects genotype-specific differences between 11BP395 and ‘Mona’. Specifically, the wild genotype showed better growth preservation, greater stability of chlorophyll and carotenoids, lower oxidative stress markers, and upregulation of key stress-responsive genes than the sensitive cultivars. Thus, wild barley represents a valuable source of tolerance traits that could be harnessed to enhance drought resilience in elite germplasm. These enhanced molecular responses involved the activation of hormonal signaling pathways and protein kinase cascades, calcium-dependent networks, elevated osmoprotectant production, polyphenol biosynthesis, and dynamic regulation of aquaporin isoforms, though interpreting them as fundamental genetic or epigenetic strategies of wild barley would require additional comparative evidence. These mechanisms underpin drought tolerance and improved physiological performance, and the transcriptomic insights offer resources for understanding barley adaptation and potential trait transfer between genotypes, though broader genotypic validation is needed to confirm the relevance of the identified genes. Beyond these biological insights, our findings also provide clear opportunities for practical crop improvement. The drought-responsive genes identified in this study provide a foundation for developing functional molecular markers to efficiently screen tolerant germplasm. In addition, key resistance loci from wild barley can be introgressed into cultivated backgrounds through hybridisation, although determining their broader relevance to drought resilience requires evaluation across additional genotypes. Establishing doubled haploid (DH) lines carrying favourable alleles would further accelerate the fixation and deployment of these traits in breeding pipelines. Together, these strategies highlight the translational value of tolerant genotypes as a potential genetic resource for developing high-performing cultivars adapted to water-limited environments.

## Figures and Tables

**Table 1:** Element concentrations in grains and leaves of drought-tolerant and drought-sensitive barley genotypes under normal and drought stress conditions.

	Elements	Elements	Sensitive		Rate of Change	Tolerant		Rate of Change
	(mg kg^−1^ DW)	(mg kg^−1^ DW)	Control	Drought		Control	Drought	
Grains	K	Potassium	5285 ^c^	5038 ^d^	−4.67	6365 ^b^	6661 ^a^	4.65
Leaves			27,385 ^c^	15,050 ^d^	−45.04	29,990 ^b^	38,023 ^a^	26.78
Grain	P	Phosphorus	3840 ^a^	3516 ^b^	−8.43	3114 ^c^	3562 ^c^	14.38
Leaves			3097 ^a^	1220 ^d^	−60.6	2440^c^	2731 ^b^	11.92
Grains	S	Sulfur	1250 ^b^	1297 ^b^	3.76	1276 ^b^	1453 ^a^	13.87
Leaves			3100 ^b^	2225 ^c^	−28.22	3082 ^b^	3313 ^a^	7.49
Grains	Ca	Calcium	817 ^c^	1039 ^b^	27.17	1226 ^a^	1159 ^ab^	−5.46
Leaves			7502 ^a^	6995 ^b^	−6.75	6005 ^d^	6509 ^c^	8.39
Grains	Mn	Manganese	25 ^b^	29 ^b^	16	30 ^b^	55 ^a^	83.33
Leaves			82 ^bc^	89^b^	8.53	70 ^c^	107 ^a^	52.85
Grains	Mg	Magnesium	1479 ^a^	1312 ^d^	−11.29	1346 ^c^	1455 ^b^	8.09
Leaves			1801 ^a^	1125 ^b^	−37.53	1703 ^a^	1803 ^a^	5.87
Grains	Mo	Molybdenum	0.8 ^b^	1.6 ^a^	100	0.7 ^b^	1 ^b^	42.85
Leaves			1.9 ^b^	1.5 ^b^	−21.05	1.9 ^b^	3.1 ^a^	63.15
Grains	Fe	Iron	1233 ^d^	2253 ^c^	82.72	3421 ^b^	3649 ^a^	13.28
Leaves			4437 ^c^	6939 ^b^	56.38	5255 ^c^	10,089 ^a^	91.98
Grains	Zn	Zinc	29 ^a^	26 ^b^	−10.349	21 ^c^	25 ^b^	19.04
Leaves			20 ^a^	11 ^b^	−45	18 ^a^	19 ^a^	5.55
Grains	Al	Aluminum	139 ^c^	296 ^a^	112.94	306 ^a^	230 ^b^	−24.83
Leaves			454 ^a^	423 ^a^	−6.82	442 ^a^	362 ^b^	−18.09
Grains	Ba	Barium	6^a^	4 ^c^	−33.33	5 ^b^	3 ^d^	−40
Leaves			29 ^a^	19 ^b^	−34.48	14 ^bc^	11 ^c^	−21.42
Grains	Cu	Copper	4 ^b^	4 ^b^	0	3 ^b^	9 ^a^	200
Leaves			14 ^a^	8 ^c^	−42.85	13 ^ab^	12 ^b^	−7.69
Grains	Na	Sodium	481 ^c^	535 ^c^	11.22	697 ^ab^	1041 ^a^	49.35
Leaves			7868 ^b^	7409 ^b^	−5.83	10,099 ^a^	11,134 ^a^	10.24
Grains	Sr	Strontium	10 ^c^	8.8 ^b^	−12	13.2 ^a^	10.8 ^b^	−18.18
Leaves			128 ^a^	107 ^b^	−16.4	86.4 ^c^	89.6 ^c^	3.7

In each column, means followed by the same letters are not significantly different according to the LSD test at 0.05 level of probability. Color legend: Green = increase under drought stress; Red = decrease under drought stress; Blue = no significant change under drought stress.

**Table 2:** Unique enriched functional categories (annotated by GO term) of DEGs in wild and cultivated barley under drought stress conditions.

GO	Term Name	Term ID	Term Size	*p*-Value (−Log10)	Intersection Size
Wild barley: uniquely up-regulated DEGs					
MF	Adenyl ribonucleotide binding	GO:0032559	3212	4.05 × 10^−12^	63
BP	Regulation of cellular respiration	GO:0043457	9	2.20 × 10^−13^	5
CC	Membrane	GO:0016020	41,234	4.48 × 10^−7^	237
Wild barley: uniquely down-regulated DEGs					
MF	Catalytic activity	GO:0003824	10,556	1.63 × 10^−29^	456
BP	Biological process	GO:0008150	15,228	4.30 × 10^−21^	576
CC	Plastid	GO:0009536	924	1.29 × 10^−23^	91
Cultivated barley: uniquely up-regulated DEGs					
MF	protein serine/threonine phosphatase activity	GO:0004722	105	1.67 × 10^−7^	10
BP	response to abiotic stimulus	GO:0009628	435	3.06 × 10^−12^	32
CC	-	-	-	-	-
Cultivated barley: uniquely down-regulated DEGs					
MF	Catalytic activity	GO:0003824	10,556	3.62 × 10^−27^	427
BP	Cell wall organization or biogenesis	GO:0071554	326	2.09 × 10^−28^	64
CC	Extracellular region	GO:0005576	676	2.48 × 10^−32^	101

**Table 3:** Primer sequences employed for RT-qPCR amplification in barley.

Gene Candidate	Gene Symbol	Gene Name	Forward Primer Sequence (5′–3′)
			Reverse Primer Sequence (5′–3′)
HORVU.MOREX.r3.5HG0507410	*Dhn1*	Dehydrin 1	TCAGCACACTTCCACAGCCA
			GCCGTACTCCTCCACCTTGT
HORVU.MOREX.r3.6HG0545620	*HvBip-70*	Heat shock protein 70kD, peptide-binding domain superfamily	TACCACCTACTCATGTGTCG
			TCCTTGTCCTCAAACTTTCTTC
HORVU.MOREX.r3.1HG0075860	*HvbZIPx*	bZIP domain-containing transcription factor	AGTCCGCAAGCTCTGTTGTT
			GAGCCACAATGACAGCAAAA
HORVU.MOREX.r3.5HG0523050	*HvPht1-6*	PHT1 phosphate transporters	TTCGGGCCAAACTCCACCAC
			TACCCGTGGTCCACCTTGCT
HORVU.MOREX.r3.5HG0497680	*HvCBF6*	AP2/ERF transcription factors CBF/DREB	CCTCAACTTCGCCGACTCCG
			GGTGCAAGAGACCGAGAGCG
Reference gene	*β-tubulin*	Constitutive proteins of microtubules	GTCCACCCACTCCCTCCTTG
			CGGCGGCAGATGTCATAGATG

## Data Availability

The RNA-seq datasets generated during the current study are publicly available and have been deposited in the NCBI Sequence Read Archive (SRA). The transcriptome raw data can be accessed via the BioProject accession number PRJNA1479468 (including accession numbers: SRR39211783, SRR39211784, SRR39211785, SRR39211786, SRR39211787, SRR39211788, SRR39211789, and SRR39211790). Data is provided within the manuscript or Supplementary Materials.

## Supplementary Materials

The following supporting information can be downloaded at: https://www.mdpi.com/article/10.3390/plants15203087/s1, Detailed descriptions of the protocols used for physiological trait measurements are provided as Supplementary Materials (Methods S1–S8). Figure S1. Principal component analysis (PCA) plot of DEseq2- processed samples for (a) the drought tolerant wild genotype and (b) the drought sensitive cultivar ‘Mona’. Figure S2. Heatmap of the sample-to-sample distances generated from the DEseq2 output for (a) drought tolerant wild genotype and (b) drought sensitive cultivar ‘Mona’. Figure S3. Classification of significant genotype × treatment interaction genes into biological response categories. Figure S4. Volcano plot of genotype × treatment interaction effects. Interaction log_2_FC (wild minus Mona) is shown on the X axis and −log_10_ (FDR) on the Y axis. Green and orange points indicate genes with significant interaction effects (FDR ≤ 0.05), reflecting stronger drought responses in the wild genotype or in cv. Mona, respectively; grey points represent non significant genes. Selected genes with large interaction coefficients are labeled. Figure S5. KEGG pathway classification of the differentially expressed genes (DEGs) (a) up-regulated and (b) down-regulated genes in the drought-tolerant genotype; as well as (c) up-regulated and (d) down-regulated genes in the sensitive barley cultivar grown under normal and drought stress conditions. Figure S6. KEGG plant hormone signal transduction pathway under drought stress in barley. Green boxes represent up regulated DEGs in the drought tolerant genotype, while pink boxes indicate down regulated DEGs in the drought sensitive genotype and Blue boxes denote genes showing opposite regulation patterns between the two genotypes (up regulated in the tolerant and down regulated in the sensitive genotype). EC numbers of the corresponding enzymes are provided in each box. Figure S7. (a) KEGG flavonoid biosynthesis pathway in the drought tolerant barley genotype. Green boxes represent up regulated DEGs in the tolerant genotype within the flavonoid biosynthesis pathway. (b) KEGG flavonoid biosynthesis pathway in the drought sensitive genotype. Green boxes indicate up regulated DEGs, whereas pink boxes represent down regulated DEGs in the sensitive genotype. EC numbers of the corresponding enzymes are shown in each box. Table S1: RNA-seq data of two barley genotypes (wild and cultivated) under control and drought stress conditions. Table S2. Genes with significant genotype × treatment interaction effects identified from the volcano plot between wild and cv. Mona, selected based on large |interaction log_2_FC| values and FDR ≤ 0.05. Table S3. Functional annotation of some of the differentially expressed (up- and down-regulated) genes involved in Abscisic Acid (ABA) Signaling in wild (tolerant) and cultivated (sensitive) genotypes of barley grown under control and drought stress conditions. Table S4. Functional annotation of some of the differentially expressed (up- and down-regulated) protein kinases genes in wild (tolerant) and cultivated (sensitive) genotypes of barley grown under control and drought stress conditions. Table S5. Functional annotation of some of the differentially expressed (up- and down-regulated) genes involved in Phytohormone Biosynthesis in wild (tolerant) and cultivated (sensitive) genotypes of barley grown under control and drought stress conditions. Table S6. Functional annotation of some of the differentially expressed (up- and down-regulated) Transcription Factors genes in wild (tolerant) and cultivated (sensitive) genotypes of barley grown under control and drought stress conditions. Table S7. Functional annotation of some of the differentially expressed (up- and down-regulated) ion transporters and channels genes in wild (tolerant) and cultivated (sensitive) genotypes of barley grown under control and drought stress conditions. Table S8. Functional annotation of the differentially expressed (up- and down-regulated) genes encoding reactive oxygen species scavenging proteins in wild (tolerant) and cultivated (sensitive) genotypes of barley grown under control and drought stress conditions. Table S9. Functional annotation of the differentially expressed (up- and down-regulated) genes involved in osmotic homeostasis and accumulation of compatible solutes in wild (tolerant) and cultivated (sensitive) genotypes of barley grown under control and drought stress conditions.

## Abbreviations

The following abbreviations are used in this manuscript:

ABA	Abscisic acid
APX	Ascorbate peroxidase
Car	Carotenoids
CAT	Catalase
Chla	chlorophyll a
Chlb	chlorophyll b
DPPH	2,2-diphenyl-1-picrylhydrazyl
DEG	Differentially Expressed Gene
GB	Gibberellin
POX	Guaiacol peroxidase
H_2_O_2_	Hydrogen peroxide
HPLC	High-performance liquid chromatography
IAA	Indole-3-acetic acid
ICP-MS	Inductively Coupled Plasma Mass Spectrometry
KEGG	Kyoto Encyclopedia of Genes and Genomes
MDA	Malondialdehyde
MSI	Membrane stability index
Pro	Proline
ROS	Reactive oxygen species
RWC	Relative water content
TF	Transcription factor
TFC	total flavonoid content
TPC	total phenolic content
