# Supplementary material for: Physiological and Transcriptome Analysis of Drought Tolerance in Barley
Source: Plants (Basel). 2026 Oct 9;15(20):3087. doi: 10.3390/plants15203087 (PMC13708358; doi:10.3390/plants15203087)
Supplement: Supplementary file 1 [file plants-15-03087-s001.zip › plants-4525958-supplementary.pdf]

Supplementary materials

Include:

Supplementary Methods

Supplementary Figures

Supplementary Tables

Supplementary Methods

Physiological traits measurement protocols used in this study

Methods S1. Membrane stability index (MSI) & Relative water content (RWC)

Leaf electrolyte leakage was determined using the method described by Sairam (1). Leaf tissues of barley seedlings (0.2 g) were placed in test tubes containing 10 ml of double-distilled water and incubated in a water bath at 40 °C for 30 minutes. The electrical conductivity of the bathing solution (C1) was then recorded. The samples were subsequently boiled at 100 °C for 10 minutes, after which the final electrical conductivity (C2) was measured. The membrane stability index (MSI) was calculated using the following equation: MSI % = [1 – (C1/C2)] × 100.

Relative water content (RWC) of leaf tissues was determined following the classical method described by Barrs and Weatherley (2). Fresh leaf discs (approximately 0.2 g) were excised and immediately weighed to obtain the fresh weight (FW). The samples were then floated on distilled water in Petri dishes and kept under low light at room temperature for 4 hours to reach full turgidity. After gently blotting the surface moisture, the turgid weight (TW) was recorded. Subsequently, the samples were dried in an oven at 70 °C for 24 hours to obtain the dry weight (DW). The RWC was calculated using the following equation:

RWC (%)=[FW−DW/TW−DW]×100

Methods S2. Chlorophylls (Chl) and Carotenoids (Car)

Chlorophylls and carotenoids were extracted in 80% acetone following the standard procedure described by Lichtenthaler (3), using fresh leaves samples collected at the grain-filling stage. The absorbance of the extracts was recorded at 663, 646, and 470 nm with a UV–visible spectrophotometer (U-1800, Hitachi, Japan). Pigment concentrations (C) were subsequently calculated and expressed as mg g^{-1} fresh mass (FM) using the following equations:

Chl a: Ca = 12.21 × A663 – 2.81 × A646 × V/1,000 × W

Chl b: Cb = 20.13 × A646 – 5.03 × A663 × V/1,000 × W

Car: Cx+c = [(1,000 × A470) – (1.9 Ca) – (63.14 Cb)]/ 214 × V/1,000 × W

where A is absorbance, V is the volume of acetone (ml), and W is the fresh mass (g).

Methods S3. Proline content

Proline content was determined following the ninhydrin-based method described by Bates et al. (4), using fresh leaf tissues collected at the grain-filling stage. Leaf samples (0.2 g) were homogenized in 10 ml of 3% aqueous sulfosalicylic acid and centrifuged at 10,000 rpm for 10 minutes. The resulting supernatant was mixed with 2 ml of acid ninhydrin reagent and 2 ml of glacial acetic acid, and the mixture was incubated in a boiling water bath at 96 °C for 1 hour. The reaction was then terminated by placing the tubes in an ice bath, after which the chromophore was extracted with 4 ml of toluene. The absorbance of the pink-red toluene phase was measured at 520 nm using a UV–visible spectrophotometer (U-1800, Hitachi, Japan) against a toluene blank. Proline concentration was calculated from a standard curve and expressed as mg g^{-1} fresh mass (FM).

Methods S4. Abscisic acid extraction and quantification

Abscisic acid (ABA) was extracted by adding 10 mL of 80% methanol and 0.1 g of polyvinylpyrrolidone (PVP) to 1 g of fresh plant tissue, followed by homogenization at 4 °C. The homogenate was centrifuged at 4000 g for 15 minutes, and the resulting supernatant was collected and adjusted to pH 8. Methanol was removed under vacuum, and the remaining residue was dissolved in 5 mL of deionized water. This extraction step was repeated twice. In the final purification stage, ethyl acetate was added to the extract, and the solvent was again evaporated. The remaining precipitate was dissolved in 1 mL of a solution containing 3% methanol and 0.1 M acetic acid (5). The solution was then filtered through a 0.45 μm membrane filter and injected into a reversed-phase HPLC column (Diamonsil C18, 5 μm, 25 cm × 4.6 mm) using an HPLC system (Unicam-Crystal-200, UK). A gradient mobile phase of methanol and acetic acid (3–97%) was applied at a flow rate of 4 mL/min, and detection was performed using a diode-array detector. A high-purity ABA standard (99.97%, Sigma) was used to calibrate retention time and peak area, and ABA quantification in samples was based on the corresponding calibration curve.

Extraction and quantification of IAA and GB

Extraction

A total of 2 g of plant tissue was ground in cold 80% (v/v) aqueous methanol and thoroughly homogenized. The homogenate was extracted with 30 mL of cold 80% methanol under dark conditions at 4 °C. The extract was centrifuged at 5000 rpm for 15 minutes at 4 °C, and the resulting supernatant was carefully collected (6). The supernatant was then filtered through filter paper to remove any remaining particulates. The retained solids were sequentially washed with methanol–acetic acid (100:1, v/v), methanol–water–acetic acid (50:50:1, v/v/v), methanol–water–acetic acid (30:70:1, v/v/v), and finally distilled water.

A 100-mL portion of the combined filtrate was subsequently passed through ten C18 solid-phase extraction (SPE) columns (J.T. Baker, Phillipsburg, NJ, USA; 500 mg, 3 mL). The columns were preconditioned and rinsed with 10 mL of acidified water (pH 3, adjusted with acetic acid), after which phytohormones were eluted using 5 mL of an ethanol–water–acetic acid mixture (80:20:1, v/v/v). The eluate was evaporated to dryness under vacuum at room temperature, and the resulting residue was re-dissolved

in 1 mL of methanol. The final solution was filtered through a 0.45 µm Whatman glass microfiber filter prior to HPLC analysis.

HPLC Analysis

Phytohormone analysis was conducted according to the procedures outlined by Ma et al. (7) and Ge et al. (8) using a high-performance liquid chromatography (HPLC) system (Unicam Crystal 200, UK) equipped with a photodiode array (PDA) detector. A 10 µL aliquot of each prepared extract was injected onto a reversed-phase C18 column (Zorbax SB-C18, 100 Å, 3.5 µm, 150 × 2.1 mm), with the column temperature maintained at 25 °C.

Chromatographic separation began with an isocratic elution of methanol–formic acid buffer (10:90, v/v) for 5 minutes, followed by a linear gradient to methanol–formic acid buffer (30:70, v/v) over 5 minutes. This composition was held isocratically for 10 minutes before applying a second gradient to methanol–formic acid buffer (45:55, v/v) over 35 minutes. The final ratio (45:55, v/v) was maintained isocratically for an additional 15 minutes. Under these conditions, complete separation of all target phytohormones was achieved within 45 minutes.

Following each analytical run, the column was flushed with methanol–formic acid buffer (95:5, v/v) for 5 minutes and subsequently re-equilibrated with formic acid buffer–methanol (90:10, v/v) for 30 minutes. Quantification was performed based on the peak areas of authenticated standards analyzed under identical chromatographic conditions.

Methods S5. Antioxidant defense parameters

Enzymatic antioxidants (unit mg-1 protein)

Protein extraction and quantification

Total leaves protein content was determined by extracting proteins from plant tissues maintained on ice at 0–4 °C. For extraction, 1 g of frozen leaf tissue was ground in a pre-chilled mortar with 3 mL of 50 mM phosphate buffer (pH 7.2) containing 1 mM EDTA, 1 mM PMSF, and 1% PVP. The homogenate was centrifuged at 4000 g for 15 minutes at 4 °C, and the resulting supernatant was collected for protein analysis. Aliquots of the extracts were stored at −80 °C until further use.

Protein concentration was quantified using the Bradford (9) assay. In brief, 0.1 mL of protein extract was mixed with 5 mL of Bradford reagent, vortexed immediately, and the absorbance was recorded at 595 nm after 2 minutes (and within one hour) using a spectrophotometer. Protein concentration was determined using a standard curve prepared with bovine serum albumin (BSA) across a range of known concentrations. Bradford reagent was prepared by dissolving 0.1 g of Coomassie Brilliant Blue G-250 in 50 mL of 95% ethanol with continuous stirring for one hour. Subsequently, 100 mL of 85% phosphoric acid was added dropwise, and the final volume was brought to one liter with distilled water. The solution was then filtered through Whatman No. 1 filter paper and stored at 4 °C until use.

Enzyme extraction

Fresh leaf samples (0.2 g per treatment) were homogenized with a mortar and pestle to obtain the soluble extract required for enzyme activity assays. The homogenized tissue was then mixed with 2 mL of sodium phosphate buffer (10 mM, pH 7.0) supplemented with 50 mM potassium phosphate, 1 mM EDTA, 2% polyvinylpyrrolidone (PVP), 2 mM α-dithiothreitol (DTT), 50 mM Tris-HCl, and 0.2% Triton X-100. The mixture was centrifuged at 12,000 × g for 30 minutes, and the resulting supernatant was collected for antioxidant enzyme activity measurements. All enzyme assays were conducted in duplicate.

Calculation of enzyme activities and normalization based on protein content

To ensure biologically meaningful comparisons of enzyme activity, the measured activity was normalized to the protein content of each sample rather than reported solely as absolute activity. Young tissues, which contain a higher proportion of meristematic cells, typically possess greater amounts of soluble proteins and enzymes compared with mature tissues that accumulate more structural components such as lignin and insoluble proteins (10). Therefore, specific enzyme activity was expressed as units per milligram of protein (U/mg protein) (11) by dividing the volumetric enzyme activity by the protein concentration of the extract.
The enzyme activity was calculated using the following formula:

$$\text{Enzyme Activity (U)} = \frac{\Delta A \times TV \times D}{\varepsilon \times EV}$$

$$\text{Volumetric Activity (U/mL)} = \frac{\text{Enzyme Activity (U)}}{\text{Volume of extract used (mL)}}$$

$$\text{Specific Activity (U/mg protein)} = \frac{\text{Volumetric Activity (U/mL)}}{\text{Protein concentration (mg/mL)}}$$

ΔA: Change in absorbance per minute at specific wave length (nm)

TV: Total volume of the reaction mixture (mL)

D: Dilution factor

ε: Extinction coefficient for each enzyme

EV: Volume of enzyme extract used (mL)

For instance, one unit (U) of catalase activity was defined as the amount of enzyme required to decompose one μmol of hydrogen peroxide per minute at 25 °C under the specified assay conditions.

Guaiacol peroxidase activity

Guaiacol peroxidase (POX) activity was determined following the method of **Hemeda and Klein (12).** The reaction mixture contained 30 μL of enzyme extract, 2.77 mL of 50 mM phosphate buffer (pH 7.0), 100 μL of 1% hydrogen peroxide (H_2O_2), and 100 μL of 4% guaiacol. The reaction was initiated by the addition of H_2O_2, and the oxidation of guaiacol to tetraguaiacol was monitored spectrophotometrically at 470 nm after one minute. Enzyme activity was calculated using the extinction coefficient of tetraguaiacol (25.5 $mM^{-1} \cdot cm^{-1}$) and expressed as units per milligram of protein.

Ascorbate peroxidase activity

Ascorbate peroxidase (APX) activity was determined following the procedure described by **Nakano and Asada (13).** The reaction mixture consisted of 50 mM potassium phosphate buffer (pH 7.0), 0.1 mM

hydrogen peroxide (H_2O_2), 0.5 mM ascorbic acid, and 150 μL of enzyme extract. The decrease in absorbance at 290 nm, corresponding to the oxidation of ascorbic acid, was recorded to monitor the reaction. APX activity was calculated using an extinction coefficient of 2.8 $mM^{-1}\cdot cm^{-1}$. One unit (U) of APX activity was defined as the amount of enzyme required to oxidize 1 μmol of ascorbate per minute. Enzyme activity was expressed as units per milligram of protein.

Catalase activity

Catalase (CAT) activity was measured following the procedure described by Aebi (14). The reaction mixture contained 50 mM potassium phosphate buffer (pH 7.0), 15 mM hydrogen peroxide (H_2O_2), and 100 μL of enzyme extract. The breakdown of H_2O_2 was monitored by recording the decline in absorbance at 240 nm at 30-second intervals for up to 2 minutes using a spectrophotometer. CAT activity was calculated using an extinction coefficient (ε) of 40 $mM^{-1}\cdot cm^{-1}$. One unit (U) of catalase activity was defined as the amount of enzyme that decomposes 1 μmol of H_2O_2 per minute. Enzyme activity was expressed as units per milligram of protein.

Methods S6. Non-enzymatic antioxidants

Total phenolic content

Total phenolic content (TPC) was determined using the Folin–Ciocalteu method as described by Kiani et al. (15). For extraction, 100 mg of powdered barley tissue was mixed with 10 mL of 80% methanol and shaken gently at 150 rpm for 24 hours at 25 °C. Following filtration, 0.5 mL of the methanolic extract was combined with 2.5 mL of diluted Folin–Ciocalteu reagent (1:10 with distilled water) and 2 mL of 7.5% sodium carbonate solution. The reaction mixture was incubated at 45 °C for 15 minutes, after which absorbance was recorded at 765 nm using a spectrophotometer. Tannic acid was used as the standard for calibration, and TPC was expressed as milligrams of tannic acid equivalents (TAE) per gram of extract on a dry-weight basis.

Total flavonoid content

Total flavonoid content (TFC) was determined using the aluminum chloride colorimetric method described by **Kiani et al. (15).** Briefly, 125 μL of the diluted extract was mixed with 300 μL of 5% $NaNO_2$ solution and allowed to stand for 5 minutes. Then, 600 μL of 10% $AlCl_3$ was added to the mixture. Finally, 2 mL of 1 M NaOH and 2 mL of distilled water were added to bring the reaction to its final volume. The absorbance of the resulting solution was measured at 510 nm using a spectrophotometer. TFC was calculated and expressed as milligrams of quercetin equivalents (QUE) per gram of extract.

DPPH radical scavenging assay

The antioxidant activity of the samples was assessed using the DPPH (2,2-diphenyl-1- picrylhydrazyl) free radical scavenging assay, following the procedure of Brand-Williams et al. (16). In brief, 0.1 mL of each

plant extract—prepared at concentrations of 50, 100, and 300 ppm—was mixed with 5 mL of 0.1 mM DPPH solution in methanol. The mixture was thoroughly shaken and incubated at room temperature for 30 minutes. Absorbance was then recorded at 517 nm using a UV–visible spectrophotometer, with 80% methanol used as the blank.

A negative control containing only methanol and DPPH solution was included for comparison. Butylated hydroxytoluene (BHT) at different concentrations served as the positive standard. For the BHT standard curve, 1 mL of 0.2 mM DPPH solution in methanol was prepared, and its absorbance was measured at 517 nm against the blank.

The percentage of DPPH radical scavenging activity was calculated using the following equation:

$$\text{DPPH radical scavenging activity (\%)} = \frac{A_B - A_A}{A_B} \times 100$$

where A_A and A_B represent the absorbance values of the DPPH solution in the presence of the plant extract and the control sample, respectively.

Methods S7. Oxidative stress indicators

TBA-reactive substances (TBARS)

Lipid peroxidation products (TBARS), expressed as MDA equivalents,, was quantified using the thiobarbituric acid (TBA) reactive substances assay following the method of **Taulavuori et al. (17).** Because the TBA assay detects a mixture of aldehydic lipid peroxidation products rather than MDA specifically, the values are reported as MDA equivalents in accordance with Johnston et al. [18]. In brief, 0.5 g of powdered leaf tissue was homogenized in 5 mL of 50 mM potassium phosphate buffer (pH 7). The homogenate was centrifuged at 15,000 × g for 10 minutes at 4 °C, after which 0.5 mL of the resulting supernatant was mixed with 1.5 mL of TBA solution (0.5% TBA in 20% w/v trichloroacetic acid). The mixture was heated at 95 °C for 30 minutes and then rapidly cooled on ice for 30 minutes to stop the reaction. Absorbance was recorded at 532 and 600 nm to correct for nonspecific turbidity. MDA concentration was expressed as nmol per gram of sample.

Hydrogen peroxide

The hydrogen peroxide (H_2O_2) content was determined according to the method described by Velikova et al. (19). Briefly, 0.1 g of leaf tissue was homogenized in 3 mL of 0.5% (w/v) TCA and subsequently centrifuged. Then, 500 µL of the supernatant was mixed with 500 µL of 10 mM phosphate buffer (pH 7.5) and 2 mL of 1 M potassium iodide (KI). The reaction mixture was kept in the dark at room temperature for 1 hour. Absorbance was recorded at 410 nm, and the H_2O_2 concentration was expressed as nmol per gram of tissue.

Methods S8. Elemental analysis using ICP-MS

Elemental analysis of plant tissues was performed using Inductively Coupled Plasma Mass Spectrometry (ICP-MS) following the standard operating procedures of ZarAzma Analytical Laboratory (Tehran, Iran) (20). Dried and finely ground samples (0.20 g) were digested in a closed-vessel microwave digestion system using a mixture of concentrated nitric acid (HNO_3, 65%, Merck, Suprapur®) and

hydrogen peroxide (H_2O_2, 30%) at a ratio of 6:2 (v/v). The digestion program consisted of a 10-min ramp to 120 °C, a 10-min ramp to 160 °C, and a 20-min hold at 180 °C, with a maximum pressure of 40 bar. After cooling, digests were diluted to a final volume of 25 mL with Milli-Q water (18.2 MΩ). Elemental quantification was carried out using an ICP-MS equipped with a collision/reaction cell under ZarAzma's standard operating conditions, including an RF power of 1550 W, nebulizer gas flow of 0.95–1.00 L min^{-1}, auxiliary gas flow of 0.80 L min^{-1}, sample uptake rate of 0.3 mL min^{-1}, and dwell times of 20–50 ms depending on the analyte; helium collision mode was applied for Fe, Zn, Cu, and Mn, while macro-elements were measured in standard mode. Calibration curves were generated using Merck Certipur® multi-element standards at 0, 10, 50, 100, and 500 µg L^{-1}. Quality assurance and control procedures included reagent and procedural blanks, duplicate samples, certified reference material (NIST 1573a Tomato Leaves), and spike recovery tests within the acceptable range of 90–110%. Limits of detection (LOD) and quantification (LOQ) were calculated as three and ten times the standard deviation of reagent blanks, respectively. To ensure comparability across samples, elemental concentrations were normalized to both the initial dry mass used for digestion (0.20 g of each sample) and the final digest volume (25 mL). This normalization accounts for differences in sample mass and dilution volume, allowing the ICP-MS output (µg L^{-1} in solution) to be converted to dry-weight concentrations. All elemental concentrations were expressed on a dry weight basis (mg kg^{-1} DW), following the standard reporting format of ZarAzma Analytical Laboratory.

References

1. Sairam, R.K. Effect of moisture-stress on physiological activities of two contrasting wheat genotypes. *Biol. Plant.* **1994**, 36, 117–122
2. Barrs, H.D.; Weatherley, P.E. A re-examination of the relative turgidity technique for estimating water deficits in leaves. *Australian J. Biol. Sci.* **1962**, 15, 413–428. doi: 10.1071/BI9620413
3. Lichtenthaler H.K.; Wellburn A.R. Determinations of total carotenoids and chlorophylls a and b of leaf extracts in different solvents. *Biochem. Soc. T.* **1983**, 11, 591–592. https://doi.org/10.1042/bst0110591
4. Bates L.S.; Waldren R.P.; Teare I.D. Rapid determination of free proline for water-stress studies. *Plant Soil* **1973**, 39, 205–207. https://doi.org/10.1007/BF00018060
5. Li, X.-J.; Yang, M.-F.; Chen, H.; Qu, L.-Q.; Chen, F.; Shen, S.-H. Abscisic acid pretreatment enhances salt tolerance of rice seedlings proteomic evidence. Biochimica et Biophysica Acta (BBA). *Proteins Proteomics* **2010**, 1804, 929–940. 10.1016/j.bbapap.2010.01.004
6. Shindy, W. W.; Smith, O. E. Identification of plant hormones from cotton ovules. *Plant Physiol.* **1975**, 55, 550–554. https://doi.org/10.1104/pp.55.3.550
7. Ma, Z.; Ge, L.; Lee, A.S.; Yong, J.W.H.; Tan, S.N.; Ong, E. S. Simultaneous analysis of different classes of phytohormones in coconut (*Cocos nucifera* L.) water using high-performance liquid chromatography and liquid chromatography–tandem mass spectrometry after solid-phase extraction. *Analytica Chimica Acta* **2008**, 610, 274–281. https://doi.org/10.1016/j.aca.2008.01.045
8. Ge, L.; Yong, J.W.H.; Tan, S.N.; Yang, X. H.; Ong, E. S. Analysis of some cytokinins in coconut (*Cocos nucifera* L.) water by micellar electrokinetic capillary chromatography after solid-phase extraction. *J. Chromatogr.* **2004**, 1048, 119–126. 10.1016/j.chroma.2004.07.031

9. Bradford, M. M. A. 1976. rapid and sensitive method for the quantitation of microgram quantities of protein utilizing the principle of protein-dye binding. *Anal. Biochem*. **1976**, 72, 248–254. 10.1016/0003-2697(76)90527-3

10. Yousefzadeh Najafabadi, M.; Ehsanzadeh, P. Photosynthetic and antioxidative upregulation in drought-stressed sesame (*Sesamum indicum* L.) subjected to foliar-applied salicylic acid. *Photosynthetica* **2017**, 55, 611–622. https://doi.org/10.1007/s11099-017-0673-8

11. Kermani, S. G.; Saeidi, G.; Sabzalian, M.R.; Gianinetti, A. Drought stress influenced sesamin and sesamolin content and polyphenolic components in sesame (*Sesamum indicum* L.) populations with contrasting seed coat colors. *Food Chem*. **2019**, 289, 360–368. 10.1016/j.foodchem.2019.03.004

12. Hemeda, H.M.; Klein, B. P. Effects of naturally occurring antioxidants on peroxidase activity of vegetable extracts. *J. Food Sci*. **1990**, 55, 184–185. https://doi.org/10.1111/j.1365-2621.1990.tb06048.x

13. Nakano, Y.; Asada, K. Hydrogen peroxide is scavenged by ascorbate-specific peroxidase in spinach chloroplasts. *Plant Cell Physiol*. **1981**, 22, 867–880. https://doi.org/10.1093/oxfordjournals.pcp.a076232

14. Abi, H. Catalase in vitro. Methods in Enzymology, **1984,** 105, 121–126. https://doi.org/10.1016/S0076-6879(84)05016-3

15. Kiani, R.; Arzani, A.; Mirmohammady Maibody, S.A.M. Polyphenols, flavonoids, and antioxidant activity involved in salt tolerance in wheat, aegilops cylindrica and their amphidiploids. *Front. Plant Sci*. **2021**, 12, 646221–646234. https://doi.org/10.3389/fpls.2021.646221

16. Brand-Williams, W.; Cuvelier, M.E.; Berset, C. Use of free radical method to evaluate antioxidant activity. *LWT-Food Sci. Technol*. **1995**, 28, 25–30. 10.1016/S0023-6438(95)80008-5

17. Taulavuori, E.; Hellstrom, E.; Taulavuori, K.; Laine, K. Comparison of two methods used to analyse lipid peroxidation from *Vaccinium myrtillus* (L.) during snow removal, reacclimation and cold acclimation. *J. Exp. Bot*. **2001**, 52, 2375-2380. https://doi.org/10.1093/jexbot/52.365.2375

18. Johnston, J.W.; Horne, S.; Harding, K.; Benson, E.E. Evaluation of the 1-methyl-2-phenylindole colorimetric assay for aldehydic lipid peroxidation products in plants: Malondialdehyde and 4-hydroxynonenal. *Plant Physiol. Biochem*. **2007**, *45*, 108–112. https://doi.org/10.1016/j.plaphy.2007.01.011

19. Velikova, V.; Yordanov, I.; Edreva, A.J.P.S. 2000. Oxidative stress and some antioxidant systems in acid rain-treated bean plants: protective role of exogenous polyamines. *Plant Sci*. **2000**, 151, 59–66. https://doi.org/10.1016/S0168-9452(99)00197-1

20. Montaser, A. Ed. *Inductively coupled plasma mass spectrometry*; John Wiley & Sons. New York, NY, USA, **1998**

Supplementary Figures

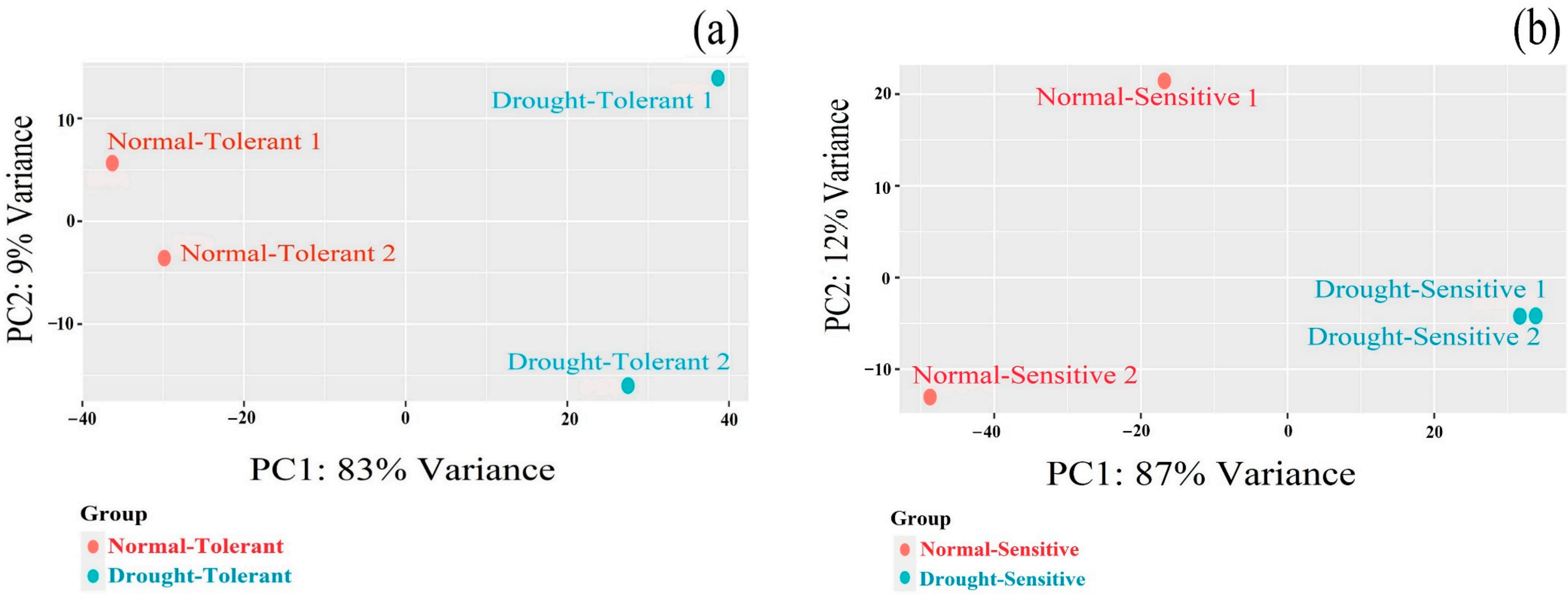

Figure S1. Principal component analysis (PCA) plot of DEseq2- processed samples for (**a**) the drought-tolerant wild genotype and (**b**) the drought-sensitive cultivar 'Mona'.

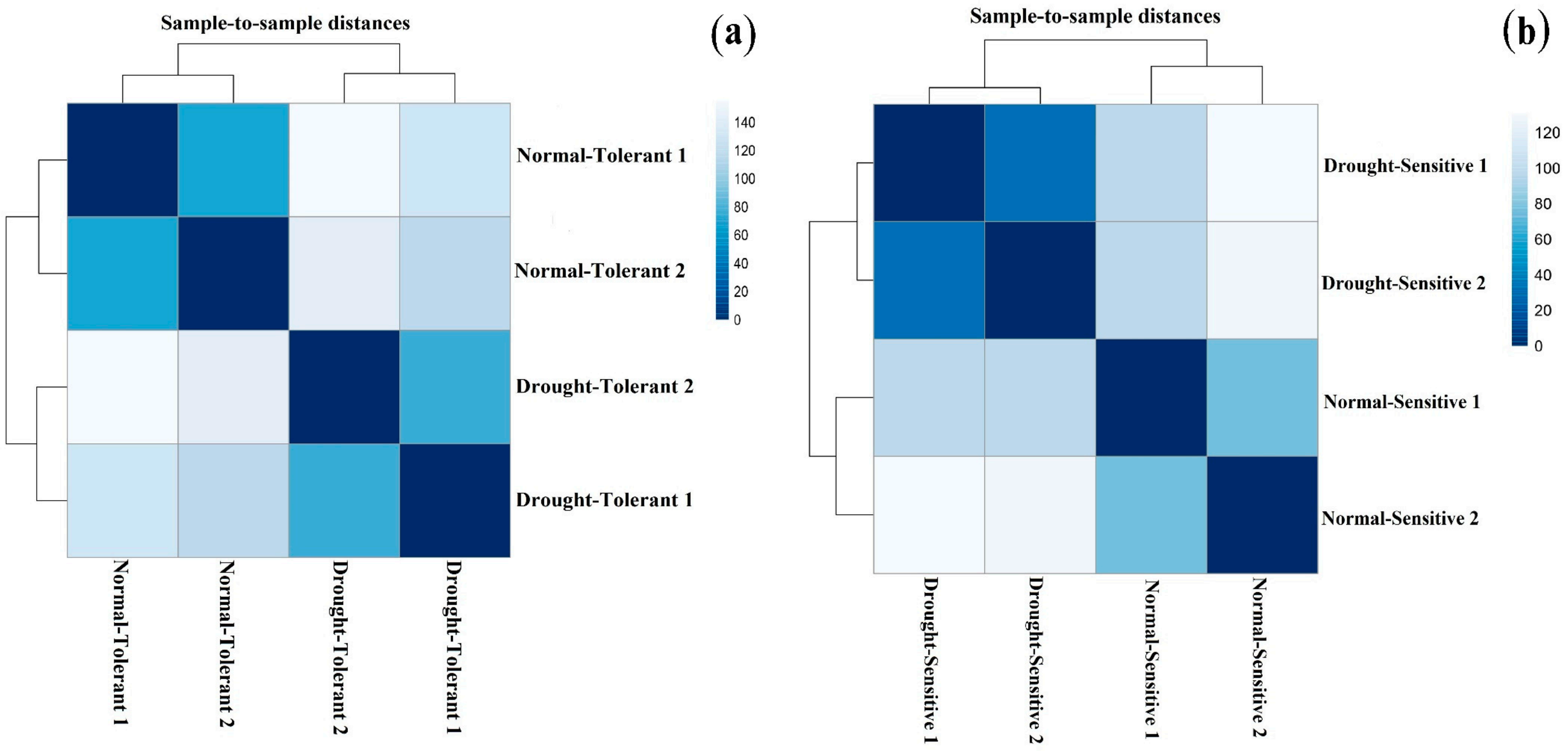

Figure S2. Heatmap of the sample-to-sample distances generated from the DEseq2 output for (**a**) drought-tolerant wild genotype and (**b**) drought-sensitive cultivar 'Mona'.

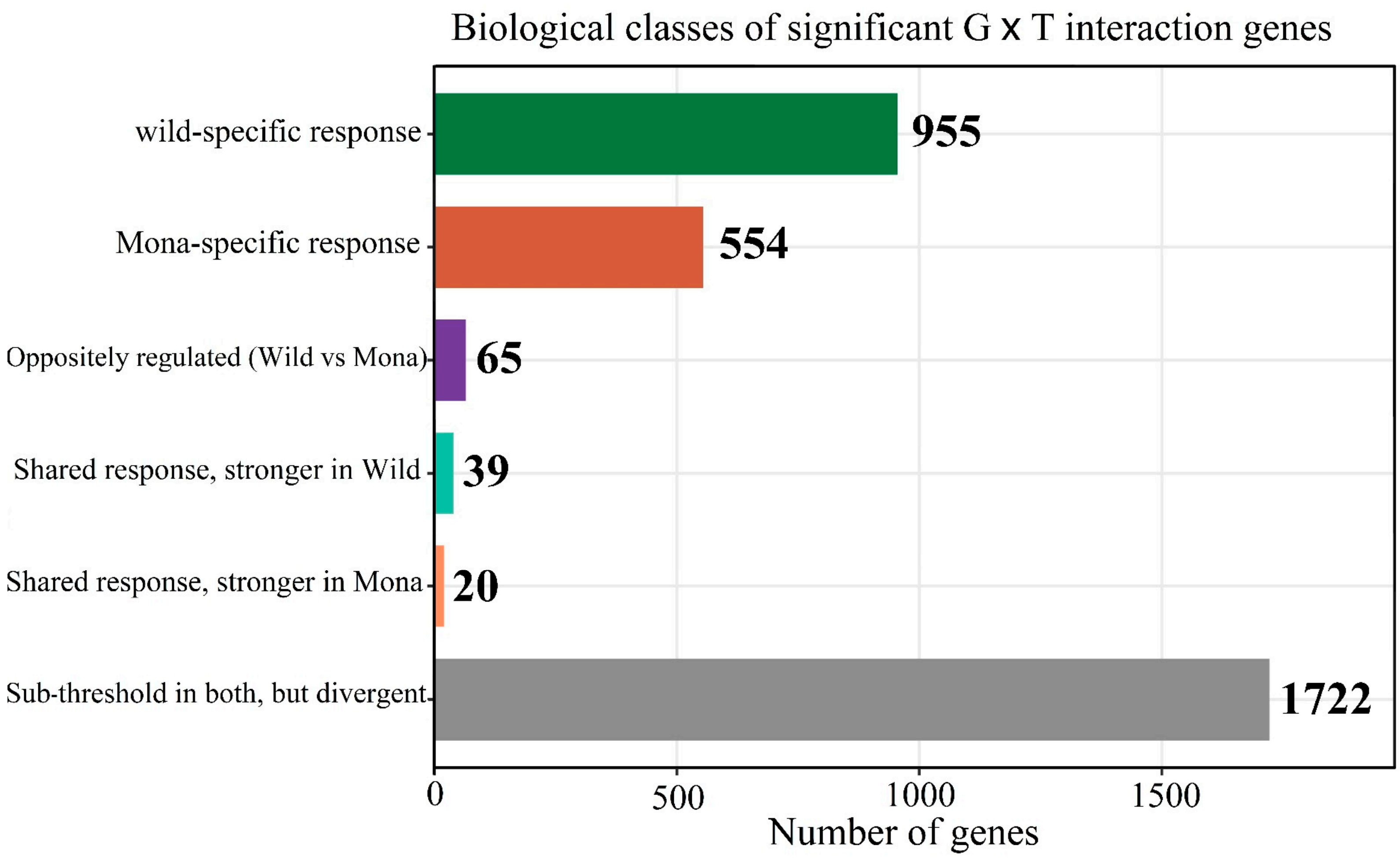

Figure S3. Classification of significant genotype × treatment interaction genes into biological response categories.

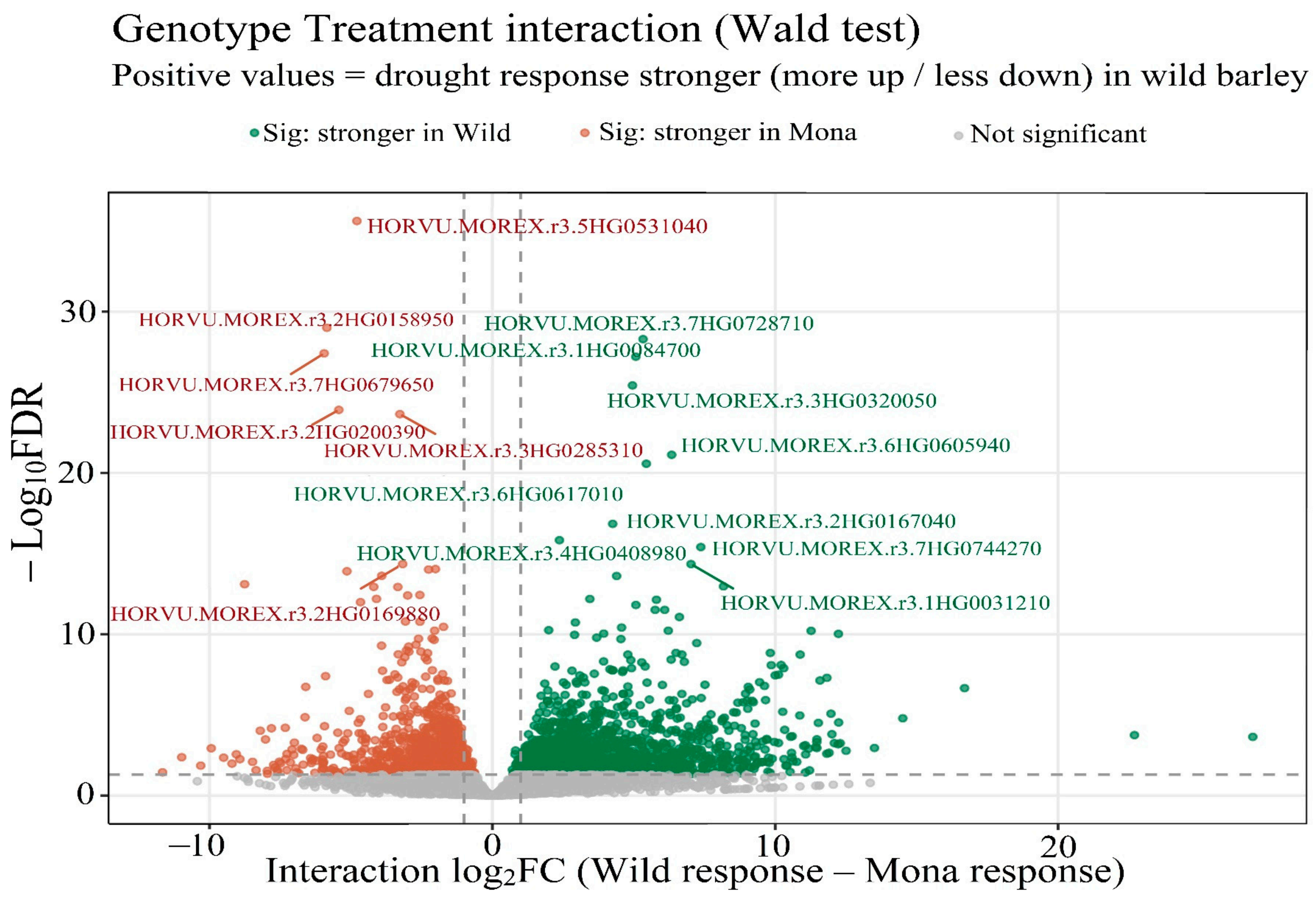

Figure S4. Volcano plot of genotype × treatment interaction effects. Interaction $\log_2$FC (wild minus Mona) is shown on the X-axis and $-\log_{10}$ (FDR) on the Y-axis. Green and orange points indicate genes with significant interaction effects (FDR ≤ 0.05), reflecting stronger drought responses in the wild genotype or in cv. Mona, respectively; grey points represent non-significant genes. Selected genes with large interaction coefficients are labeled.

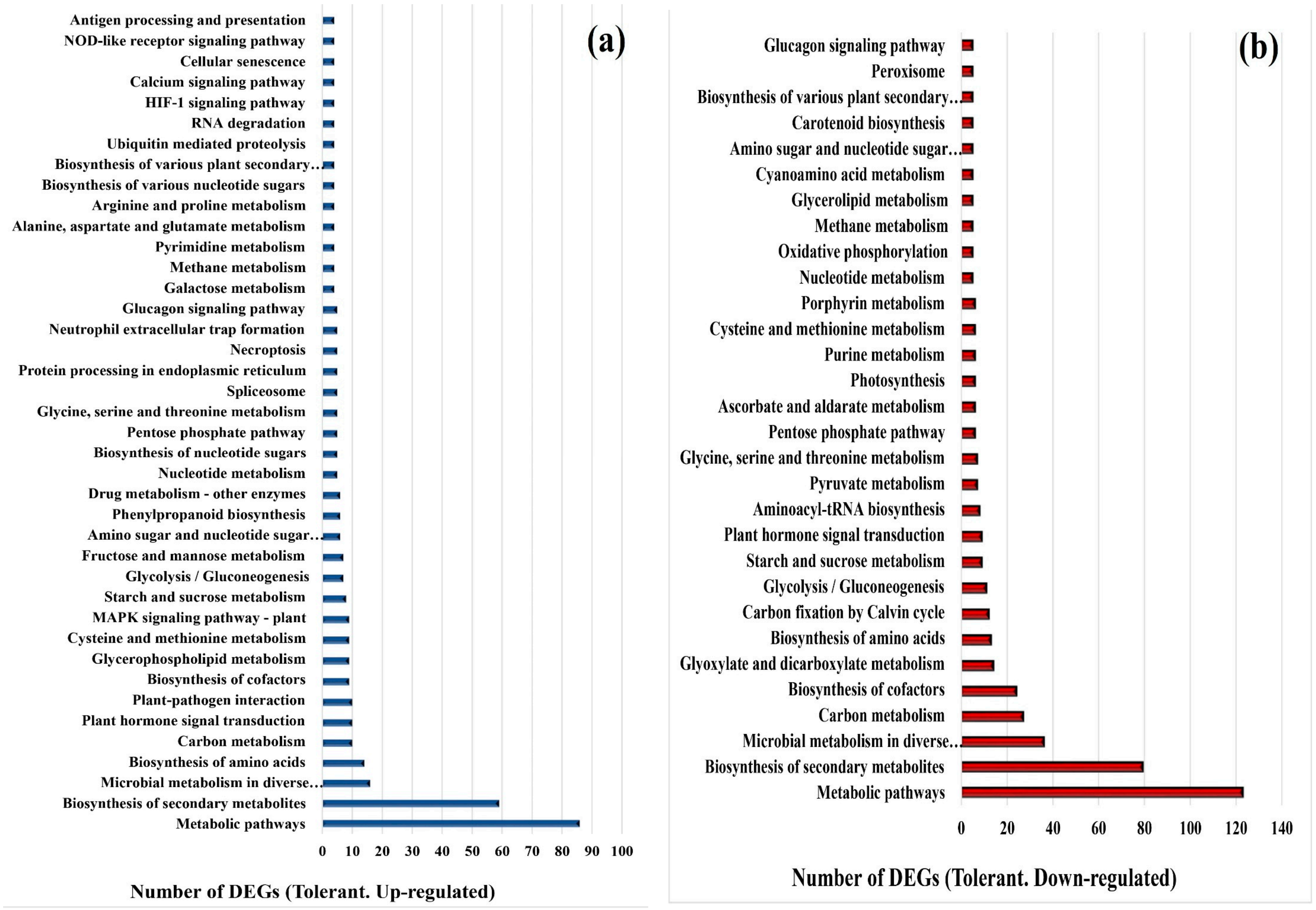
(a)
Antigen processing and presentation
NOD-like receptor signaling pathway
Cellular senescence
Calcium signaling pathway
HIF-1 signaling pathway
RNA degradation
Ubiquitin mediated proteolysis
Biosynthesis of various plant secondary…
Biosynthesis of various nucleotide sugars
Arginine and proline metabolism
Alanine, aspartate and glutamate metabolism
Pyrimidine metabolism
Methane metabolism
Galactose metabolism
Glucagon signaling pathway
Neutrophil extracellular trap formation
Necroptosis
Protein processing in endoplasmic reticulum
Spliceosome
Glycine, serine and threonine metabolism
Pentose phosphate pathway
Biosynthesis of nucleotide sugars
Nucleotide metabolism
Drug metabolism - other enzymes
Phenylpropanoid biosynthesis
Amino sugar and nucleotide sugar…
Fructose and mannose metabolism
Glycolysis / Gluconeogenesis
Starch and sucrose metabolism
MAPK signaling pathway - plant
Cysteine and methionine metabolism
Glycerophospholipid metabolism
Biosynthesis of cofactors
Plant-pathogen interaction
Plant hormone signal transduction
Carbon metabolism
Biosynthesis of amino acids
Microbial metabolism in diverse…
Biosynthesis of secondary metabolites
Metabolic pathways
0 10 20 30 40 50 60 70 80 90 100
Number of DEGs (Tolerant. Up-regulated)
(b)
Glucagon signaling pathway
Peroxisome
Biosynthesis of various plant secondary…
Carotenoid biosynthesis
Amino sugar and nucleotide sugar…
Cyanoamino acid metabolism
Glycerolipid metabolism
Methane metabolism
Oxidative phosphorylation
Nucleotide metabolism
Porphyrin metabolism
Cysteine and methionine metabolism
Purine metabolism
Photosynthesis
Ascorbate and aldarate metabolism
Pentose phosphate pathway
Glycine, serine and threonine metabolism
Pyruvate metabolism
Aminoacyl-tRNA biosynthesis
Plant hormone signal transduction
Starch and sucrose metabolism
Glycolysis / Gluconeogenesis
Carbon fixation by Calvin cycle
Biosynthesis of amino acids
Glyoxylate and dicarboxylate metabolism
Biosynthesis of cofactors
Carbon metabolism
Microbial metabolism in diverse…
Biosynthesis of secondary metabolites
Metabolic pathways
0 20 40 60 80 100 120 140
Number of DEGs (Tolerant. Down-regulated)

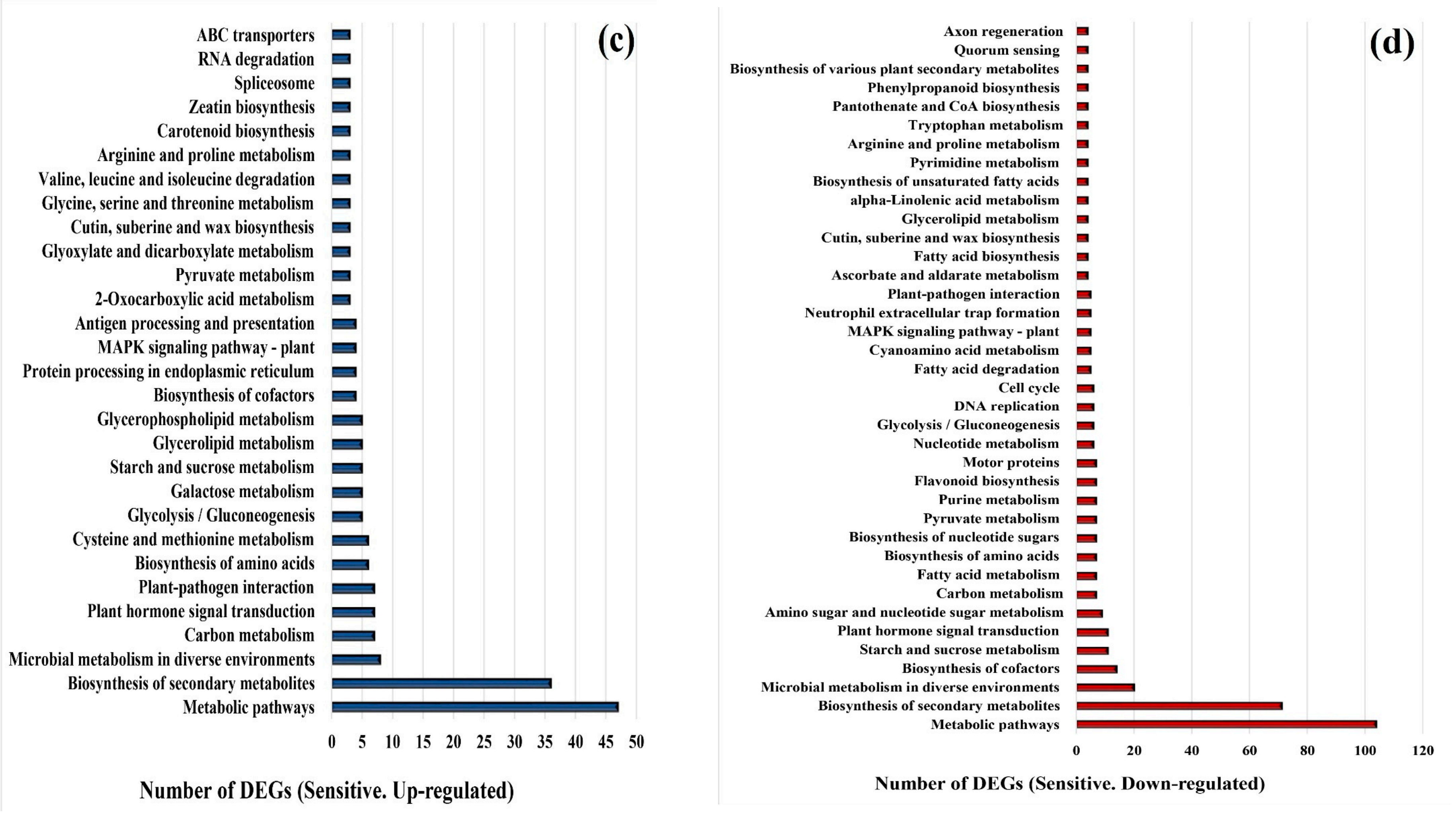

Figure S5. KEGG pathway classification of the differentially expressed genes (DEGs) **(a)** up-regulated and **(b)** down-regulated genes in the drought-tolerant genotype; as well as **(c)** up-regulated and **(d)** down-regulated genes in the sensitive barley cultivar grown under normal and drought stress conditions.

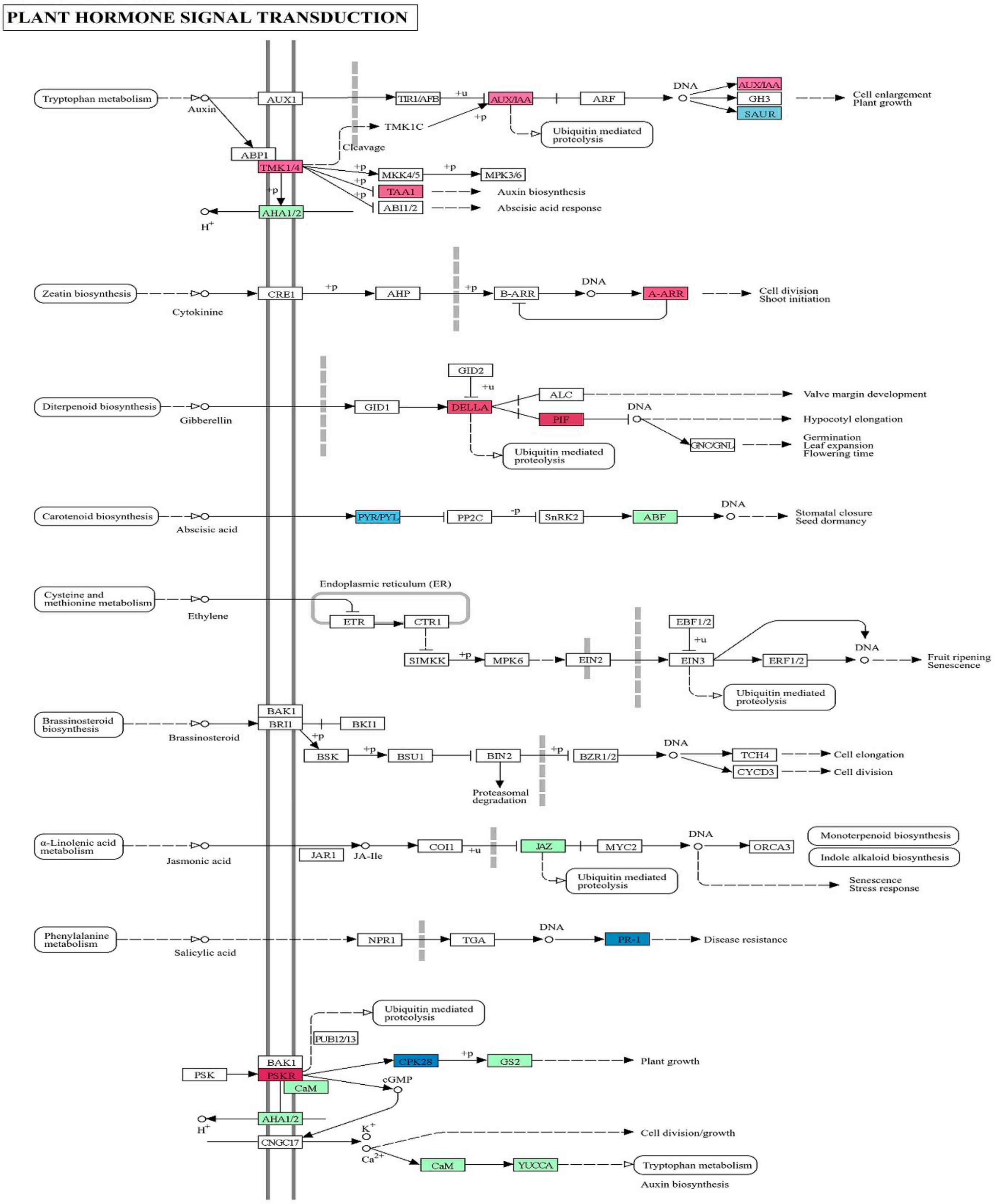

Figure S6. KEGG plant hormone signal transduction pathway under drought stress in barley. Green boxes represent up-regulated DEGs in the drought-tolerant genotype, while pink boxes indicate down-regulated DEGs in the drought-sensitive genotype and Blue boxes denote genes showing opposite regulation patterns between the two genotypes (up-regulated in the tolerant and down-regulated in the sensitive genotype). EC numbers of the corresponding enzymes are provided in each box.

(a)

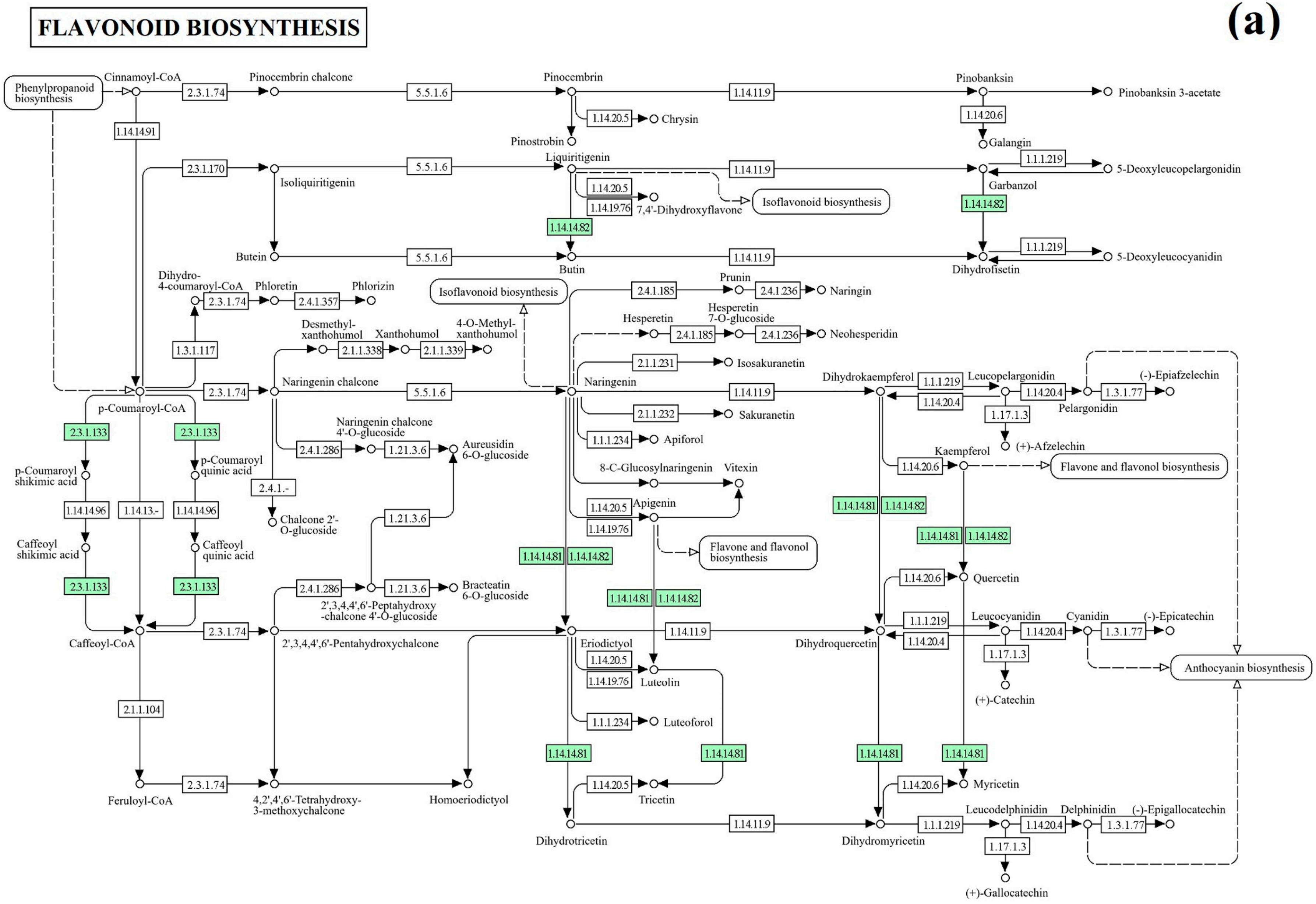
FLAVONOID BIOSYNTHESIS
Phenylpropanoid biosynthesis
Cinnamoyl-CoA
2.3.1.74
Pinocembrin chalcone
5.5.1.6
Pinocembrin
1.14.11.9
Pinobanksin
Pinobanksin 3-acetate
1.14.14.91
1.14.20.5
Chrysin
Pinostrobin
1.14.20.6
Galangin
2.3.1.170
Liquiritigenin
1.1.1.219
5-Deoxyleucopelargonidin
Isoliquiritigenin
1.14.19.76
7,4'-Dihydroxyflavone
Isoflavonoid biosynthesis
Garbanzol
1.14.14.82
Butein
Butin
Dihydrofisetin
5-Deoxyleucocyanidin
Dihydro-4-coumaroyl-CoA
Phloretin
2.4.1.357
Phlorizin
Prunin
2.4.1.185
2.4.1.236
Naringin
Hesperetin
Hesperetin 7-O-glucoside
Neohesperidin
1.3.1.117
Desmethyl-xanthohumol
2.1.1.338
Xanthohumol
2.1.1.339
4-O-Methyl-xanthohumol
2.1.1.231
Isosakuranetin
Naringenin chalcone
Naringenin
Dihydrokaempferol
Leucopelargonidin
(-)-Epiafzelechin
1.14.20.4
1.3.1.77
Pelargonidin
p-Coumaroyl-CoA
2.3.1.133
2.1.1.232
Sakuranetin
1.17.1.3
Naringenin chalcone 4'-O-glucoside
(+)-Afzelechin
2.4.1.286
1.21.3.6
Aureusidin 6-O-glucoside
1.1.1.234
Apiforol
Kaempferol
p-Coumaroyl shikimic acid
p-Coumaroyl quinic acid
2.4.1.-
8-C-Glucosylnaringenin
Vitexin
Flavone and flavonol biosynthesis
1.14.14.96
1.14.13.-
Chalcone 2'-O-glucoside
Apigenin
1.14.14.81
Caffeoyl shikimic acid
Caffeoyl quinic acid
Quercetin
Bracteatin 6-O-glucoside
2',3,4,4',6'-Peptahydroxy-chalcone 4'-O-glucoside
Leucocyanidin
Cyanidin
(-)-Epicatechin
Caffeoyl-CoA
2',3,4,4',6'-Pentahydroxychalcone
Eriodictyol
Dihydroquercetin
Anthocyanin biosynthesis
Luteolin
(+)-Catechin
2.1.1.104
Luteoforol
Myricetin
Feruloyl-CoA
4,2',4',6'-Tetrahydroxy-3-methoxychalcone
Homoeriodictyol
Tricetin
Leucodelphinidin
Delphinidin
(-)-Epigallocatechin
Dihydrotricetin
Dihydromyricetin
(+)-Gallocatechin

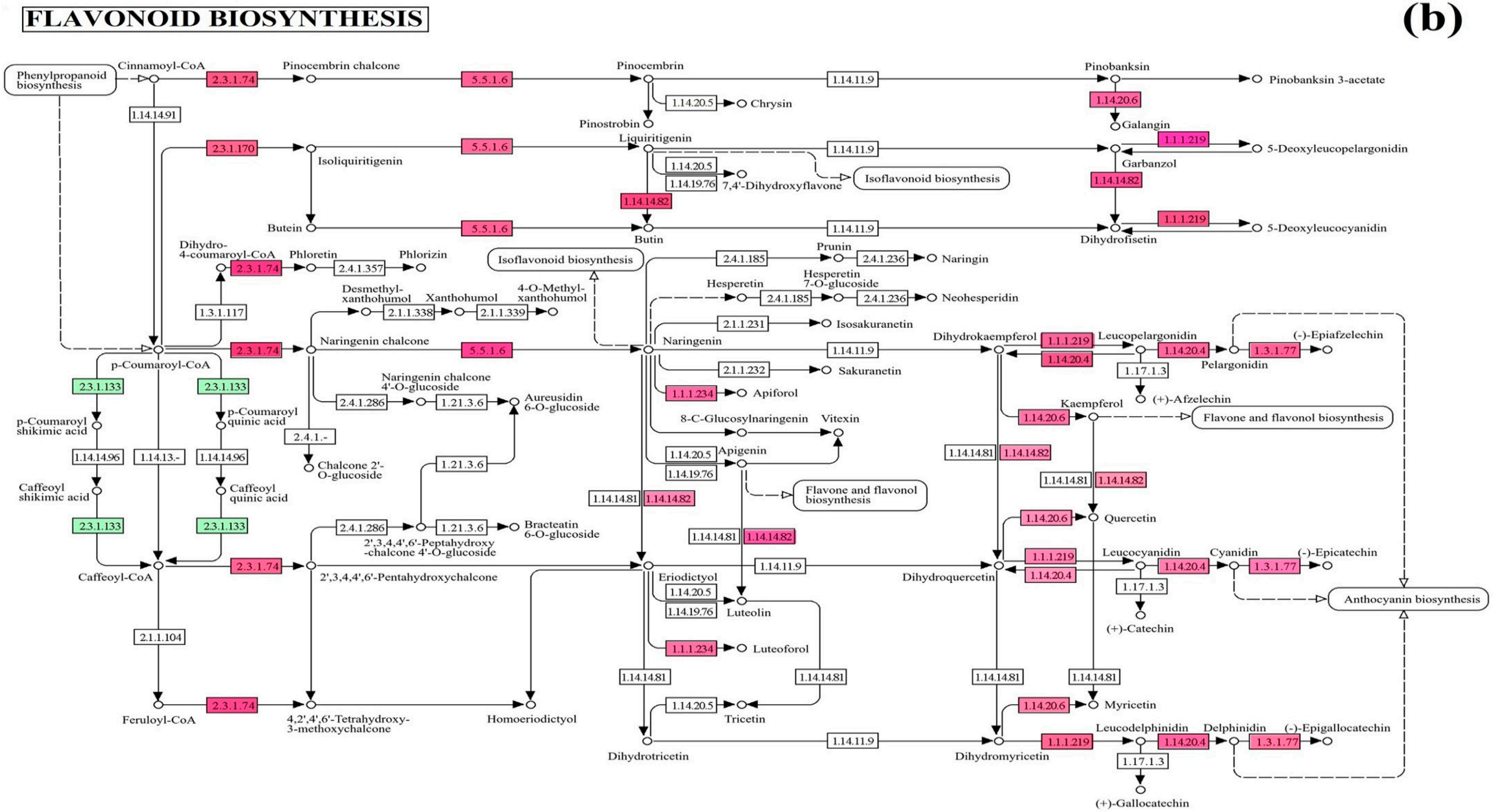

Figure S7. (**a**) KEGG flavonoid biosynthesis pathway in the drought-tolerant barley genotype. Green boxes represent up-regulated DEGs in the tolerant genotype within the flavonoid biosynthesis pathway. **(b)** KEGG flavonoid biosynthesis pathway in the drought-sensitive genotype. Green boxes indicate up-regulated DEGs, whereas pink boxes represent down-regulated DEGs in the sensitive genotype. EC numbers of the corresponding enzymes are shown in each box.

Supplementary Tables

Table S1. RNA-seq data of two barley genotypes (wild and cultivated) under control and drought stress conditions

Genotype	Treatment	Raw Paired-end read	GC(%)	Q30(%)	Clean Paired-end read	Mapping rate (%)
Wild	Normal I	45542689	54.47	93.71	45540568	90.61
	Normal II	40757646	55.14	93.59	40756912	90.30
	Drought stress I	49197598	55.22	94.65	49194174	91.35
	Drought stress II	71139228	56.15	93.87	71136488	83.23
Cultivated	Normal I	39930670	54.60	93.93	39930160	82.64
	Normal II	41917536	54.35	93.86	41816988	83.84
	Drought stress I	50621112	57.01	94.52	50618684	91.10
	Drought stress II	52299234	56.37	93.86	52297144	89.90

Table S2. Genes with significant genotype × treatment interaction effects identified from the volcano plot between wild and cv. Mona, selected based on large |interaction log_2FC| values and FDR ≤ 0.05.

**Gene ID**	**Tolerant (Log2FC)**	**Tolerant P-adj**	**Sensitive (Log2FC)**	**Tolerant P-adj**	**Gene function**
HORVU.MOREX.r3.7HG0728710	0.507	0.369	-4.850	$3.71E^{-51}$	Xyloglucan endotransglucosylase_hydrolase
HORVU.MOREX.r3.1HG0084700	6.251	$1.50E^{-30}$	1.600	$3.85E^{-12}$	Uncharacterized protein
HORVU.MOREX.r3.3HG0320050	3.484	$2.65E^{-08}$	-1.479	0.0001	EF-hand domain-containing protein
HORVU.MOREX.r3.6HG0605940	1.439	0.062	-4.855	$2.33E^{-39}$	Xyloglucan endotransglucosylase_hydrolase
HORVU.MOREX.r3.6HG0617010	6.613	0.004	-5.635	0.349	-
HORVU.MOREX.r3.2HG0167040	4.851	$8.08E^{-14}$	0.902	0.026	Phi-1
HORVU.MOREX.r3.4HG0408980	1.775	$7.32E^{-05}$	-0.534	0.055	Methyltransferase
HORVU.MOREX.r3.7HG0744270	6.1407	$7.09E^{-19}$	-1.179	0.382	-
HORVU.MOREX.r3.1HG0031210	4.345	$4.76E^{-08}$	-2.699	$1.09E^{-18}$	Late embryogenesis abundant protein LEA-2 subgroup domain-containing protein
HORVU.MOREX.r3.5HG0531040	-4.242	$7.96E^{-19}$	0.593	0.238	-
HORVU.MOREX.r3.2HG0158950	-4.509	$1.64E^{-19}$	1.152	0.101	Transcription factor
HORVU.MOREX.r3.7HG0679650	-8.527	$1.09E^{-61}$	-2.550	$5.36E^{-17}$	JmjC domain-containing protein
HORVU.MOREX.r3.2HG0200390	-5.311	$3.77E^{-29}$	0.168	0.757	Beta-fructofuranosidase
HORVU.MOREX.r3.3HG0285310	-0.890	0.0609	2.382	$2.85E^{-25}$	ubiquitin-protein ligase
HORVU.MOREX.r3.2HG0169880	-1.679	0.0003	1.496	$3.65E^{-08}$	T-complex protein 11

Table S3. Functional annotation of some of the differentially expressed (up- and down-regulated) genes involved in Abscisic Acid (ABA) Signaling in wild (tolerant) and cultivated (sensitive) genotypes of barley grown under control and drought stress conditions.

**Gene ID**	**Gene function**	**Gene symbol**	**Tolerant (Log2FC)**	**Sensitive (Log2FC)**
HORVU.MOREX.r3.7HG0665280	Annexin	*HvAnn1*	3.402	0.378
HORVU.MOREX.r3.2HG0181160	Caleosin-related family Y protein		5.586	-
HORVU.MOREX.r3.7HG0669590	Caleosin-related family Y protein		2.466	-
HORVU.MOREX.r3.3HG0320050	Calcium-binding EF-hand family protein	*HvCML10*	3.484	-1.479
HORVU.MOREX.r3.3HG0270160	Calcium-binding EF-hand family protein	*HvCML12*	5.080	-
HORVU.MOREX.r3.1HG0059290	Calcium-binding EF-hand family protein	*HvCML15*	3.829	3.895
HORVU.MOREX.r3.5HG0502280	Calcium-binding EF-hand family protein	*HvCML21*	5.153	-
HORVU.MOREX.r3.2HG0177660	Calcium-binding EF-hand family protein	*HvCML22*	3.058	-
HORVU.MOREX.r3.3HG0226060	Calcium-binding EF-hand family protein	*HvCML27*	4.202	3.123
HORVU.MOREX.r3.5HG0486640	Calcium-binding EF-hand family protein	*CML30*		
HORVU.MOREX.r3.3HG0322130	Calcium-binding EF-hand family protein	*HvCML31*	7.725	-
HORVU.MOREX.r3.3HG0322140	Calcium-binding EF-hand family protein	*HvCML31*	7.004	-
HORVU.MOREX.r3.3HG0322070	Calcium-binding EF-hand family protein	*HvCML31*	7.227	-
HORVU.MOREX.r3.2HG0190500	Calcium-binding EF-hand family protein		3.075	1.638
HORVU.MOREX.r3.1HG0020310	Calcium-binding EF-hand family protein		2.911	1.418
HORVU.MOREX.r3.5HG0507280	Calcium-binding Copine C-terminal domain-containing protein		9.469	-
HORVU.MOREX.r3.3HG0322150	Calcium-binding EF-hand family protein		-	5.303
HORVU.MOREX.r3.5HG0522160	Calcium-binding EF-hand family protein		-	2.499
HORVU.MOREX.r3.5HG0509810	Calmodulin -like protein 10	*HvCaM10*	2.090	-
HORVU.MOREX.r3.5HG0484690	Calmodulin -like protein 3	*HvCaM3*	2.056	-
HORVU.MOREX.r3.5HG0433130	Calmodulin -like protein 11	*HvCaM11*	2.902	-
HORVU.MOREX.r3.1HG0077790	Calcium- transporting ATPase 13		5.907	0.879
HORVU.MOREX.r3.5HG0457980	Calcineurin B-like protein 6			
HORVU.MOREX.r3.1HG0057470	Calcineurin B-like protein, Calcium sensor protein, Reguration of potassium uptake by CBL1-CIPK23 comple	*(Os10g0564 800)*		

Table S4. Functional annotation of some of the differentially expressed (up- and down-regulated) protein kinases genes in wild (tolerant) and cultivated (sensitive) genotypes of barley grown under control and drought stress conditions

Gene ID	Gene function	Gene symbol	Tolerant (Log2FC)	Sensitive (Log2FC)
HORVU.MOREX.r3.3HG0249430	AGC_RSK-2		6.033	-
HORVU.MOREX.r3.5HG0517140	CAMK_CDPK		3.719	-3.426
HORVU.MOREX.r3.6HG0627610	CAMK_CDPK		6.247	-
HORVU.MOREX.r3.1HG0084710	CMGC_CDKL-Os		5.966	-
HORVU.MOREX.r3.4HG0331780	RLK-Pelle_CrRLK1L-1		6.386	-
HORVU.MOREX.r3.1HG0075260	RLK-Pelle_DLSV		7.523	-
HORVU.MOREX.r3.2HG0097540	RLK-Pelle_DLSV		6.539	-
HORVU.MOREX.r3.2HG0102660	RLK-Pelle_DLSV		6.344	-
HORVU.MOREX.r3.7HG0738650	RLK-Pelle_DLSV		7.153	-
HORVU.MOREX.r3.3HG0295950	RLK-Pelle_LRR-I-1		6.617	-
HORVU.MOREX.r3.3HG0225880	RLK-Pelle_LRR-III		6.634	-
HORVU.MOREX.r3.4HG0331920	RLK-Pelle_LRR-XI-1		5.022	-
HORVU.MOREX.r3.4HG0331930	RLK-Pelle_LRR-XI-1		8.242	-
HORVU.MOREX.r3.5HG0465610	RLK-Pelle_LRR-XI-1		5.784	-
HORVU.MOREX.r3.7HG0635600	RLK-Pelle_LRR-XI-1		6.552	-
HORVU.MOREX.r3.2HG0104580	RLK-Pelle_LRR-XII-1		5.956	-
HORVU.MOREX.r3.5HG0535240	RLK-Pelle_LRR-XII-1		3.697	-2.961
HORVU.MOREX.r3.6HG0546920	RLK-Pelle_RLCK-VIIa-2		6.725	-
HORVU.MOREX.r3.2HG0203170	RLK-Pelle_SD-2b		5.628	5.628
HORVU.MOREX.r3.5HG0419600	RLK-Pelle_SD-2b		6.429	-
HORVU.MOREX.r3.2HG0214050	RLK-Pelle_WAK		8.146	-
HORVU.MOREX.r3.6HG0632750	RLK-Pelle_WAK		5.975	-4.418
HORVU.MOREX.r3.1HG0079430	STE_STE11		7.381	-
HORVU.MOREX.r3.2HG0191490	TKL_CTR1-DRK-2		2.322	-
HORVU.MOREX.r3.3HG0273160	TKL_Gdt		3.129	-
HORVU.MOREX.r3.7HG0681170	RLK-Pelle_LRR-III		-	4.313
HORVU.MOREX.r3.7HG0711760	RLK-Pelle_LRR-XII-1		-	7.472
HORVU.MOREX.r3.2HG0192100	RLK-Pelle_SD-2b		-	6.669
HORVU.MOREX.r3.7HG0722360	RLK-Pelle_LRR-V		-2.977	-6.593
HORVU.MOREX.r3.7HG0739520	RLK-Pelle_LRR-V		-4.513	-3.718
HORVU.MOREX.r3.1HG0065070	RLK-Pelle_RKF3		-7.755	-7.255
HORVU.MOREX.r3.4HG0339400	RLK-Pelle_SD-2b		-9.977	-10.209

Table S5. Functional annotation of some of the differentially expressed (up- and down-regulated) genes involved in Phytohormone Biosynthesis in wild (tolerant) and cultivated (sensitive) genotypes of barley grown under control and drought stress conditions.

Gene ID	Gene function	Gene symbol	Tolerant (Log2FC)	Sensitive (Log2FC)
HORVU.MOREX.r3.3HG0247520	Abscisic acid receptor PYR/PYL family	PYL	5/879	-
HORVU.MOREX.r3.4HG0383820	Abscisic acid receptor PYR/PYL family	PYL	-	2/934-
HORVU.MOREX.r3.5HG0430270	Phytochrome-interacting factor	PIF , PIL	-	2/553-
HORVU.MOREX.r3.3HG0275650	DELLA protein	DELLA	-	5/148-
HORVU.MOREX.r3.1HG0057490	Serine/threonine-protein kinase SRK2	SNRK2	-	2/473
HORVU.MOREX.r3.2HG0201420	Serine/threonine-protein kinase SRK2	SNRK2	-	2/675
HORVU.MOREX.r3.1HG0080890.	Protein phosphatase 2C	PP2C	-	3/066
HORVU.MOREX.r3.2HG0095500	Protein phosphatase 2C	PP2C	-	9/252
HORVU.MOREX.r3.1HG0090670	Protein phosphatase 2C	PP2C	-	8/724
HORVU.MOREX.r3.3HG0267730	Protein phosphatase 2C	PP2C	-	2/725
HORVU.MOREX.r3.3HG0276740	Protein phosphatase 2C	PP2C	-	4/396
HORVU.MOREX.r3.3HG0284740	Protein phosphatase 2C	PP2C	-	3/478
HORVU.MOREX.r3.5HG0473680	Protein phosphatase 2C(PP2C37)	PP2C	-	6/021
HORVU.MOREX.r3.3HG0302180	Protein phosphatase 2C	PP2C	-	3/619
HORVU.MOREX.r3.4HG0389000	Protein phosphatase 2C(PP2C30)	PP2C	-	5/131
HORVU.MOREX.r3.2HG0190250	SAUR family protein	SAUR	4/294	-
HORVU.MOREX.r3.5HG0480850	SAUR family protein	SAUR	4/210	-
HORVU.MOREX.r3.6HG0598930	SAUR family protein	SAUR	3/454	2/668
HORVU.MOREX.r3.6HG0567190	SAUR family protein	SAUR	4/009	-
HORVU.MOREX.r3.2HG0184550	SAUR family protein	SAUR	2/458	2/580
HORVU.MOREX.r3.3HG0308590	SAUR family protein	SAUR	2/520	-
HORVU.MOREX.r3.6HG0619010	SAUR family protein	SAUR	2/924	-
HORVU.MOREX.r3.1HG0049910	SAUR family protein	SAUR	2/250	2/269
HORVU.MOREX.r3.6HG0558410	SAUR family protein	SAUR	2/062	-
HORVU.MOREX.r3.2HG0111000	ABA responsive element binding factor	ABF	3/744	2/547
HORVU.MOREX.r3.3HG0299650	ABA responsive element binding factor	ABF	3/281	3/685
HORVU.MOREX.r3.4HG0380200	ABA responsive element binding factor	ABF	2/129	-
HORVU.MOREX.r3.7HG0669350	Jasmonate ZIM domain-containing protein	JAZ	5/518	-
HORVU.MOREX.r3.7HG0669330	Jasmonate ZIM domain-containing protein	JAZ	5/855	-
HORVU.MOREX.r3.7HG0669300	Jasmonate ZIM domain-containing protein	JAZ	5/275	3/497
HORVU.MOREX.r3.7HG0669360	Jasmonate ZIM domain-containing protein	JAZ	5/506	-
HORVU.MOREX.r3.4HG0405240	Jasmonate ZIM domain-containing protein	JAZ	3/964	-
HORVU.MOREX.r3.7HG0669340	Jasmonate ZIM domain-containing protein	JAZ	4/482	-
HORVU.MOREX.r3.7HG0669320	Jasmonate ZIM domain-containing protein	JAZ	2/605	-
HORVU.MOREX.r3.3HG0236780	Auxin-responsive protein IAA	IAA	-	-3/368
HORVU.MOREX.r3.7HG0706260	Auxin-responsive protein IAA	IAA	-	-2/666
HORVU.MOREX.r3.5HG0509730	Auxin-responsive protein IAA	IAA	-	-6/212
HORVU.MOREX.r3.5HG0509120	A member of Aux/IAA family protein			

Table S6. Functional annotation of some of the differentially expressed (up- and down-regulated) Transcription Factors genes in wild (tolerant) and cultivated (sensitive) genotypes of barley grown under control and drought stress conditions.

Gene ID	Gene function	Gene symbol	Tolerant (Log2FC)	Sensitive (Log2FC)
HORVU.MOREX.r3.2HG0187000	AP2/ERF-ERF		4.307	-6.919

HORVU.MOREX.r3.4HG0405650	AP2/ERF-ERF	9.781	-
HORVU.MOREX.r3.7HG0665380	AP2/ERF-ERF	2.736	-
HORVU.MOREX.r3.6HG0619310	AP2/ERF-ERF	4.657	-5.221
HORVU.MOREX.r3.6HG0603700	AP2/ERF-ERF	-	-6.132
HORVU.MOREX.r3.6HG0575580	MYB	2.236	-3.768
HORVU.MOREX.r3.3HG0330150	MYB	3.062	1.468
HORVU.MOREX.r3.6HG0575580	MYB	2.236	-3.768
HORVU.MOREX.r3.7HG0750020	bHLH	6.275	-
HORVU.MOREX.r3.2HG0159040	bHLH	1.510	4.130
HORVU.MOREX.r3.5HG0486320	bHLH	4.634	-
HORVU.MOREX.r3.2HG0111000	bZIP	3.744	2.547
HORVU.MOREX.r3.7HG0746990	bZIP	4.507	-
HORVU.MOREX.r3.2HG0175130	NAC	8.029	-
HORVU.MOREX.r3.3HG0300280	NAC	6.290	-
HORVU.MOREX.r3.6HG0556820	NAC	4.505	3.804
HORVU.MOREX.r3.1HG0022790	NAC	3.869	8.722
HORVU.MOREX.r3.7HG0743280	WRKY	3.415	-
HORVU.MOREX.r3.2HG0096750	WRKY	2.974	-
HORVU.MOREX.r3.2HG0187550	HSF	4.476	-
HORVU.MOREX.r3.5HG0486440	HSF	2.791	-

Table S7. Functional annotation of some of the differentially expressed (up- and down-regulated) ion transporters and channels genes in wild (tolerant) and cultivated (sensitive) genotypes of barley grown under control and drought stress conditions

**Gene ID**	**Gene function**	**Gene symbol**	**Tolerant (Log2FC)**	**Sensitive (Log2FC)**
HORVU.MOREX.r3.3HG0282640	ATP -binding cassette (ABC) transporter	MDR-like ABC transporter	4.215	2.832
HORVU.MOREX.r3.3HG0302160	ATP -binding cassette (ABC) transporter	ABC transporter C family member 3	2.171	2.268
HORVU.MOREX.r3.7HG0728950	ATP -binding cassette (ABC) transporter	ABC transporter I family member 17	3.090	1.777
HORVU.MOREX.r3.7HG0700460	ATP -binding cassette (ABC) transporter	MDR-like ABC transporter	6.175	4.018

HORVU.MOREX.r3.3HG0270800	ATP -binding cassette (ABC) transporter	ABC transporter G family member 18	2.084	2.328
HORVU.MOREX.r3.7HG0722560	ATP -binding cassette (ABC) transporter	ABC transporter G family member 44	4.205	2.266
HORVU.MOREX.r3.6HG0558970	ATP -binding cassette (ABC) transporter	MDR-like ABC transporter	7.247	-
HORVU.MOREX.r3.3HG0288800	ATP -binding cassette (ABC) transporter	ABC transporter G family member 32	3.502	-
HORVU.MOREX.r3.2HG0205960	ATP -binding cassette (ABC) transporter	ABC transporter B family member 19	-	-3.886
HORVU.MOREX.r3.3HG0303310	ATP -binding cassette (ABC) transporter	Predicted protein	-	-4.982
HORVU.MOREX.r3.3HG0240190	ATP -binding cassette (ABC) transporter	Uncharacterized protein	-	-3.396
HORVU.MOREX.r3.4HG0386330	ATP -binding cassette (ABC) transporter	ABC transporter G family member 16	-	-3.090
HORVU.MOREX.r3.6HG0599090	Vacuolar iron transporter homolog 1		6.739	2.092
HORVU.MOREX.r3.5HG0523050	H(+)_Pi cotransporter		6.534	-
HORVU.MOREX.r3.2HG0189010	Major facilitator superfamily (MFS) profile domain-containing protein	Glycerol-3-phosphate transporter 1- related	3.559	2.650
HORVU.MOREX.r3.4HG0416390	Major facilitator superfamily (MFS) profile domain-containing protein	Carrier protein HEX6	2.097	1.486
HORVU.MOREX.r3.7HG0675560	Major facilitator superfamily (MFS) profile domain-containing protein	Inorganic phosphate transporter 1-9- related	5.132	-
HORVU.MOREX.r3.5HG0529600	H(+)_Pi cotransporter	Inorganic phosphate transporter 1-9- related	6.582	3.613
HORVU.MOREX.r3.5HG0527310	Major facilitator superfamily (MFS) profile domain-containing protein	Uncharacterized protein	3.241	-
HORVU.MOREX.r3.1HG0092680	Major facilitator superfamily (MFS) profile domain-containing protein		5.298	-
HORVU.MOREX.r3.2HG0131170	Major facilitator superfamily (MFS) profile domain-containing protein		2.474	-

Table S7 continued.

Gene ID	Gene function	Gene symbol	Tolerant (Log2FC)	Sensitive (Log2FC)
HORVU.MOREX.r3.4HG0399620	Major facilitator superfamily (MFS) profile domain-containing protein	Polyol transporter 5-Like	3.848	1.863
HORVU.MOREX.r3.7HG0648440	Major facilitator superfamily (MFS) profile domain-containing protein	Sugar transport protein 3	6.659	-
HORVU.MOREX.r3.2HG0135310	Major facilitator superfamily (MFS) profile domain-containing protein	Sugar transport protein MST6	2.832	1.756
HORVU.MOREX.r3.2HG0183740	Major facilitator superfamily (MFS) profile domain-containing protein	Sugar transporter	3.783	-
HORVU.MOREX.r3.1HG0031940	Bidirectional sugar transporter SWEET		4.128	-
HORVU.MOREX.r3.7HG0648840	Sulfate transporter 3.4-related	HvSPDT	3.610	1.297
HORVU.MOREX.r3.1HG0077790	Calcium-transporting ATPase		5.907	-
HORVU.MOREX.r3.7HG0669150	Potassium channel SKOR		2.379	2.688
HORVU.MOREX.r3.4HG0407500	Potassium transporter		5.761	-
HORVU.MOREX.r3.6HG0612700	Potassium transporter 16-related		2.840	1.612
HORVU.MOREX.r3.4HG0333170	Sodium_calcium exchanger membrane region domain-containing protein		3.359	3.682
HORVU.MOREX.r3.5HG0424860	Sodium_calcium exchanger membrane region domain-containing protein		4.017	-
HORVU.MOREX.r3.5HG0424600	Sodium_calcium exchanger membrane region domain-containing protein		10.349	-
HORVU.MOREX.r3.2HG0173350	Arsenical pump-driving ATPase	HvPHT1;4	7.106	-
HORVU.MOREX.r3.5HG0493870	Auxin efflux carrier component 5B-related		3.321	-
HORVU.MOREX.r3.6HG0609530	protein NRT1_ PTR family 7.1		7.845	-
HORVU.MOREX.r3.4HG0344730	protein NRT1_ PTR family 4.4		3.913	1.325
HORVU.MOREX.r3.1HG0065040	Plasma membrane ATPase		3.242	-
HORVU.MOREX.r3.5HG0439960	Dirigent protein		2.104	-3.583
HORVU.MOREX.r3.5HG0440000	Dirigent protein		2.194	-
HORVU.MOREX.r3.4HG0348030	Dirigent protein		2.276	-
HORVU.MOREX.r3.4HG0404410	Dirigent protein		3.503	-3.401

Table S8. Functional annotation of the differentially expressed (up- and down-regulated) genes encoding reactive oxygen species scavenging proteins in wild (tolerant) and cultivated (sensitive) genotypes of barley grown under control and drought stress conditions

Gene ID	Gene function	Gene symbol	Tolerant (Log2FC)	Sensitive (Log2FC)
HORVU.MOREX.r3.1HG0017720	Glutathione S-transferase	*GST6*	2.269	1.466
HORVU.MOREX.r3.1HG0051860	Glutathione S-transferase		2.292	5.805
HORVU.MOREX.r3.1HG0051890	Glutathione S-transferase		2.435	3.052
HORVU.MOREX.r3.1HG0051880	Glutathione S-transferase		2.045	2.588
HORVU.MOREX.r3.1HG0051910	Glutathione S-transferase		2.823	1.417
HORVU.MOREX.r3.2HG0139310	Glutathione S-transferase		4.233	1.546
HORVU.MOREX.r3.2HG0214680	Glutathione S-transferase		4.541	2.526
HORVU.MOREX.r3.3HG0281500	Glutathione S-transferase		4.009	2.017
HORVU.MOREX.r3.7HG0648120	Glutathione S-transferase		3.989	2.480
HORVU.MOREX.r3.7HG0737960	Glutathione S-transferase		2.601	-
HORVU.MOREX.r3.3HG0299990	Glutathione S-transferase		2.693	-
HORVU.MOREX.r3.5HG0516710	Glutathione S-transferase		4.427	5.153
HORVU.MOREX.r3.3HG0311650	Glutathione transferase		6.712	6.810
HORVU.MOREX.r3.3HG0221140	Glutathione S-transferase		5.551	-
HORVU.MOREX.r3.1HG0051740	Glutathione transferase		3.646	2.701
HORVU.MOREX.r3.3HG0320270	Glutathione transferase		3.484	1.751
HORVU.MOREX.r3.7HG0746410	Glutathione transferase		5.524	2.604
HORVU.MOREX.r3.1HG0054280	Peroxidase	*HvPOD*	6.401	-
HORVU.MOREX.r3.2HG0178700	Peroxidase 6	*HvPOD*	6.512	-
HORVU.MOREX.r3.2HG0099970	Chalcone synthase	(EC:2.3.1.74) CHS	-	-5.458
HORVU.MOREX.r3.2HG0099060	Chalcone synthase	(EC:2.3.1.74) CHS	-	-2.646
HORVU.MOREX.r3.2HG0207200	Chalcone synthase	(EC:2.3.1.74) CHS	-	-3.294
HORVU.MOREX.r3.7HG0649670	Chalcone synthase		-	-13.188
HORVU.MOREX.r3.2HG0099600	Chalcone synthase		-	-9.232
HORVU.MOREX.r3.5HG0525800	Chalcone-flavonone isomerase 1-related	(E5.5.1.6)	-	-2.064
HORVU.MOREX.r3.5HG0464380	Chalcone-flavonone isomerase 3-related	(E5.5.1.6)	-	-3.142
HORVU.MOREX.r3.7HG0726310	Flavonoid 3'-monooxygenase	CYP75B1	2.583	-
HORVU.MOREX.r3.6HG0549900	NPH3 domain-containing protein	Shikimate O-hydroxycinnamoyl transferase	4.377	-
HORVU.MOREX.r3.7HG0640130	Flavonoid 3'- monooxygenase	CYP75A	3.929	-
HORVU.MOREX.r3.6HG0540900	Flavonoid 3'- monooxygenase	CYP75B4 (EC:1.14.14.82)	-	-7.558

Table S8 continued.

**Gene ID**	**Gene function**	**Gene symbol**	**Tolerant (Log2FC)**	**Sensitive (Log2FC)**
HORVU.MOREX.r3.1HG0094920	Flavonoid 3'- monooxygenase	CYP75B3 (EC:1.14.14.82)	-	-4.333
HORVU.MOREX.r3.3HG0330120	Tricetin 3',4',5'-O-trimethyl transferase	Flavonoid O-methyl transferase	-	-4.882
HORVU.MOREX.r3.2HG0107650	UDP-Glycosyl transferase 79A6	Flavonol-3-o-glucoside L-rhamnosyl transferase (FG2)	2.487	-
HORVU.MOREX.r3.1HG0076150	Isoflavone reductase homolog IRL		3.404	-0.84
HORVU.MOREX.r3.6HG0619410	Fe2OG dioxygenase domain-containing protein	flavonol synthase (EC:1.14.20.6)	3.553	-4.933
HORVU.MOREX.r3.5HG0509790	2-Oxoglutarate (2OG) and FE(II)-depndent oxygenase superfamily Y protein-related	Fe2OG dioxygenase domain-containing protein anthocyanidin synthase (EC:1.14.20.4) ANS	-	-23.980
HORVU.MOREX.r3.2HG0199330	NAD-dependent epimerase_dehydratase domain-containing	Anthocyanidin reductase (EC:1.3.1.77) ANR	-	-2.723
HORVU.MOREX.r3.3HG0274170	Sterol-4-alpha-carboxylate 3-dehydrogenase, decarboxylating	bifunctional dihydroflavonol 4-reductase/flavanone 4-reductase (EC:1.1.1.219 1.1.1.234)	-	-3.670
HORVU.MOREX.r3.3HG0300590	Ferredoxin-3	*HvFdx3*	5.150	-
HORVU.MOREX.r3.5HG0530190	Ferredoxin-3	*HvFdx3*	3.479	-
HORVU.MOREX.r3.1HG0005420	Glycosyl transferase		4.103	-
HORVU.MOREX.r3.4HG0382670	Glycosyl transferase 4-related		5.931	-
HORVU.MOREX.r3.3HG0240570	Glycosyl transferase		5.926	-
HORVU.MOREX.r3.2HG0182690	Glycosyl transferase		6.288	-
HORVU.MOREX.r3.4HG0392480	Glycosyl transferase		3.334	-
HORVU.MOREX.r3.7HG0666740	Glycosyl transferase		2.634	2.712
HORVU.MOREX.r3.7HG0671400	Glycosyl transferase		3.565	-
HORVU.MOREX.r3.1HG0027330	Glycosyl transferase		5.338	3.417
HORVU.MOREX.r3.5HG0501870	Glycosyl transferase		2.841	2.094
HORVU.MOREX.r3.7HG0714870	Glycosyl transferase		2.230	-
HORVU.MOREX.r3.5HG0523930	Glycosyl transferase		2.213	-
HORVU.MOREX.r3.5HG0523250	Glycosyl transferase		3.261	-

Table S9. Functional annotation of the differentially expressed (up- and down-regulated) genes involved in osmotic homeostasis and accumulation of compatible solutes in wild (tolerant) and cultivated (sensitive) genotypes of barley grown under control and drought stress conditions.

Gene ID	Gene function	Gene symbol	Tolerant (Log2FC)	Sensitive (Log2FC)
HORVU.MOREX.r3.1HG0080020	Late embryogenesis abundant protein (LEA)	*LEA3*	5.362	-
HORVU.MOREX.r3.1HG0046460	Late embryogenesis abundant (LEA) hydroxyproline-rich glycoprotein	NDR1_HIN1-like protein 6	4.823	2.421
HORVU.MOREX.r3.1HG0031210	Late embryogenesis abundant (LEA) hydroxyproline-rich glycoprotein family	*LEA*	4.345	-2.699
HORVU.MOREX.r3.1HG0038750	Late embryogenesis abundant (LEA) hydroxyproline-rich glycoprotein family	*LEA*	-	-3.861
HORVU.MOREX.r3.2HG0135210	Late embryogenesis abundant (LEA) hydroxyproline-rich glycoprotein family	*LEA*	-	-7.933
HORVU.MOREX.r3.2HG0140470	Late embryogenesis abundant (LEA) hydroxyproline-rich glycoprotein family	*LEA*	6.567	-
HORVU.MOREX.r3.2HG0167300	Late embryogenesis abundant (LEA) hydroxyproline-rich glycoprotein family	*LEA*	-	-4.999
HORVU.MOREX.r3.2HG0209950	Late embryogenesis abundant (LEA) hydroxyproline-rich glycoprotein	Harpin induced protein 4	5.397	5.199
HORVU.MOREX.r3.5HG0527260	Late embryogenesis abundant (LEA) hydroxyproline-rich glycoprotein family	*LEA*	-	-6.113
HORVU.MOREX.r3.7HG0655170	Late embryogenesis abundant protein LEA-2	*LEA2*	-	-2.918
HORVU.MOREX.r3.7HG0725560	Late embryogenesis abundant protein LEA-2	*LEA2*	-	-4.497
HORVU.MOREX.r3.2HG0139280	Late embryogenesis abundant protein LEA-2	*LEA2*	2.311	-4.448
HORVU.MOREX.r3.6HG0622790	Dehydrin7	*HvDhn7*	-	8.705
HORVU.MOREX.r3.5HG0516830	Dehydrin9	*HvDhn9*	-	5.127
HORVU.MOREX.r3.4HG0339950	Dehydrin6	*HvDhn6*	-	5.868
HORVU.MOREX.r3.6HG0622760	Dehydrin4	*HvDhn4*	-	7.792
HORVU.MOREX.r3.5HG0507390	Dehydrin10	*HvDhn10*	7.653	-
HORVU.MOREX.r3.5HG0507410	Dehydrin1	*HvDhn1*	9.473	5.591
HORVU.MOREX.r3.3HG0305460	Dehydrin7	*HvDhn7*	8.348	-
HORVU.MOREX.r3.3HG0302060	Delta-1-pyrroline-5-carboxylate synthase	*HvP5CSB*	4.518	5.171
HORVU.MOREX.r3.3HG0319810	Pyrroline-5-carboxylate reductase	*HvP5cr3*	2.065	2.528
HORVU.MOREX.r3.3HG0237680	Heat-shock protein	Shsp	9.633	7.870
HORVU.MOREX.r3.3HG0237670	Heat-shock protein, putative	Shsp	8.446	7.718
HORVU.MOREX.r3.7HG0669480	Heat-shock protein, putative-sHSP	15.7 kDa heat shock protein, peroxisomal	6.743	3.585
HORVU.MOREX.r3.3HG0237690	SHSP domain-containing protein	Shsp	5.690	6.651
HORVU.MOREX.r3.3HG0237700	SHSP domain-containing protein	Shsp	7.972	8.308

Table S9 continued.

Gene ID	Gene function	Gene symbol	Tolerant (Log2FC)	Sensitive (Log2FC)
HORVU.MOREX.r3.4HG0389430	17.9 KDA class I heat shock protein	Shsp	3.554	2.513
HORVU.MOREX.r3.3HG0237710	SHSP domain-containing protein	Shsp	7.730	7.647
HORVU.MOREX.r3.4HG0389340	SHSP domain-containing protein	Shsp	2.207	3.082
HORVU.MOREX.r3.3HG0226110	6.9 KDA class I heat shock protein 1	Shsp	-	2.282*
HORVU.MOREX.r3.2HG0099870	Heat-shock protein 83	*HSP90*	8.326	4.322
HORVU.MOREX.r3.7HG0678460	Heat-shock protein 81-3	*HSP90*	2.870	2.951
HORVU.MOREX.r3.1HG0028320	70 kDa heat shock protein	*HvHsp110-4*	2.110	2.364
HORVU.MOREX.r3.1HG0073350	70 kDa heat shock protein	*HvHspc70-5a*	4.956	5.480
HORVU.MOREX.r3.4HG0387610	70 kDa heat shock protein	*HvHspc70-4*	7.124	7.345
HORVU.MOREX.r3.4HG0416940	Chaperone DnaK	*HvHsp70Mt70-2*	2.639	2.628
HORVU.MOREX.r3.4HG0387870	70 kDa heat shock protein	*HvHspc70-2*	4.754	5.033
HORVU.MOREX.r3.3HG0302810	70 kDa heat shock protein	*HvHspc70-5b*	8.805	9.759
HORVU.MOREX.r3.4HG0396100	70 kDa heat shock protein 8		4.548	4.584
HORVU.MOREX.r3.6HG0621200	Heat-shock protein, putative	17.4 kDa class III heat shock protein	5.900	9.859
HORVU.MOREX.r3.6HG0616950	Heat-shock protein, putative	*HvHsp20*	10.099	8.080
HORVU.MOREX.r3.7HG0664840	Heat-shock protein, putative	*HvHsp70*	6.453	-
HORVU.MOREX.r3.1HG0083950	Heat shock transcription factor	*HsfAc2*	2.704	4.906
HORVU.MOREX.r3.5HG0486440	Heat shock transcription factor		2.791	-
HORVU.MOREX.r3.5HG0497990	Heat shock transcription factor	*B-2c*	4.026	5.567
HORVU.MOREX.r3.7HG0686980	Heat shock transcription factor		5.250	6.536
HORVU.MOREX.r3.1HG0082040	Heat shock transcription factor		3.606	-
HORVU.MOREX.r3.2HG0187550	Heat shock transcription factor		4.476	-
HORVU.MOREX.r3.7HG0672710	Aquaporin NIP1-1-related		-	-4.433
HORVU.MOREX.r3.5HG0500800	Aquaporin PIP2-7- related		-	-2.191
HORVU.MOREX.r3.7HG0711310	Aquaporin -8	*HvPIP2;5*	-	-2.399
HORVU.MOREX.r3.5HG0537740	Aquaporin	*HvTIP1;1*	-	-9.813
HORVU.MOREX.r3.1HG0049580	Aquaporin NIP5-1- related		-	-2.382
HORVU.MOREX.r3.1HG0014220	Peptidase S59 domain-containing protein		8.754	2.496
HORVU.MOREX.r3.3HG0231600	Protein Detoxification		5.875	-
HORVU.MOREX.r3.2HG0187050	Protein Detoxification		3.200	-
HORVU.MOREX.r3.1HG0025110	Protein Detoxification		7.427	-
HORVU.MOREX.r3.2HG0200920	Agmatine coumaroyltransferase-2		4.063	-
HORVU.MOREX.r3.5HG0501580	Agmatine coumaroyltransferase-2		5.635	-
